# Meta-analysis To Define a Core Microbiota in the Swine Gut

**DOI:** 10.1128/mSystems.00004-17

**Published:** 2017-05-23

**Authors:** Devin B. Holman, Brian W. Brunelle, Julian Trachsel, Heather K. Allen

**Affiliations:** a Food Safety and Enteric Pathogens Research Unit, National Animal Disease Center, Agricultural Research Service, United States Department of Agriculture, Ames, Iowa, USA; b Interdepartmental Microbiology Graduate Program, Iowa State University, Ames, Iowa, USA; University of California, Riverside

**Keywords:** 16S rRNA gene, bacteria, gut microbiome, gut microbiota, livestock, meta-analysis, microbial ecology, swine

## Abstract

The swine gut microbiota encompasses a large and diverse population of bacteria that play a significant role in pig health. As such, a number of recent studies have utilized high-throughput sequencing of the 16S rRNA gene to characterize the composition and structure of the swine gut microbiota, often in response to dietary feed additives. It is important to determine which factors shape the composition of the gut microbiota among multiple studies and if certain bacteria are always present in the gut microbiota of swine, independently of study variables such as country of origin and experimental design. Therefore, we performed a meta-analysis using 20 publically available data sets from high-throughput 16S rRNA gene sequence studies of the swine gut microbiota. Next to the “study” itself, the gastrointestinal (GI) tract section that was sampled had the greatest effect on the composition and structure of the swine gut microbiota (*P* = 0.0001). Technical variation among studies, particularly the 16S rRNA gene hypervariable region sequenced, also significantly affected the composition of the swine gut microbiota (*P* = 0.0001). Despite this, numerous commonalities were discovered. Among fecal samples, the genera *Prevotella*, *Clostridium*, *Alloprevotella*, and *Ruminococcus* and the RC9 gut group were found in 99% of all fecal samples. Additionally, *Clostridium*, *Blautia*, *Lactobacillus*, *Prevotella*, *Ruminococcus*, *Roseburia*, the RC9 gut group, and *Subdoligranulum* were shared by >90% of all GI samples, suggesting a so-called “core” microbiota for commercial swine worldwide.

**IMPORTANCE** The results of this meta-analysis demonstrate that “study” and GI sample location are the most significant factors in shaping the swine gut microbiota. However, in comparisons of results from different studies, some biological factors may be obscured by technical variation among studies. Nonetheless, there are some bacterial taxa that appear to form a core microbiota within the swine GI tract regardless of country of origin, diet, age, or breed. Thus, these results provide the framework for future studies to manipulate the swine gut microbiota for potential health benefits.

## Abstract (precis)

The results of this meta-analysis demonstrate that “study” and GI sample location are the most significant factors in shaping the swine gut microbiota. However, in comparisons of results from different studies, some biological factors may be obscured by technical variation among studies. Nonetheless, there are some bacterial taxa that appear to form a core microbiota within the swine GI tract regardless of country of origin, diet, age, or breed. Thus, these results provide the framework for future studies to manipulate the swine gut microbiota for potential health benefits.

## INTRODUCTION

The mammalian gut microbiota is populated by an estimated 100 trillion bacterial cells that provide health benefits to the host through the production of short-chain fatty acids and vitamins from otherwise nondigestible food components, inhibition and prevention of colonization by pathogens, and development and maintenance of the host immune system (1–3). These properties also make the gut microbiota an attractive target for dietary interventions to improve the health and production of food-producing animals, including swine. Therefore, the characterization of the gut microbiota of swine has become an active area of research in recent years as the adaptation and availability of high-throughput sequencing methods have continued to expand.

The majority of swine gut microbiota studies to date have focused on sequencing specific hypervariable regions within the 16S rRNA gene. The myriad of studies that have sequenced the 16S rRNA gene can be readily aggregated and compared in a meta-analysis that may reveal patterns among individual studies working on similar sample types. Recent meta-analyses of the avian gut microbiota (4) and the indoor microbiota (5) have reported that study-level variables, compared to biological factors such as sample type, have the greatest effect on the microbiota. In swine, researchers have examined the effect of a number of different feed additives on the swine gut microbiota, including antimicrobial agents (6–12), amylose/amylopectin (13), calcium-phosphorus (14), distillers’ dried grains with solubles (15), and resistant starch (16). The effect of challenge with *Escherichia coli* (17) and *Salmonella* (18) on the swine gut microbiota has also been investigated. All of those studies found various degrees of change in the swine gut microbiota as a result of these treatments. Aggregating and reanalyzing the data could reveal whether alterations observed in individual studies are still detectable when combined with data from similar studies or whether they would be lost due to technical variation between studies.

Our objectives were to characterize the swine gut microbiota using 16S rRNA gene high-throughput sequencing data from gastrointestinal (GI) samples from 20 publically available studies and to determine if a core gut microbiota, defined here as being present in ≥90% of samples, exists in swine. We evaluated how technical variables, such as study design, country of origin, and hypervariable region sequenced, affect the observed swine gut microbiota.

## RESULTS AND DISCUSSION

### Meta-analysis characteristics.

**FIG 1:**
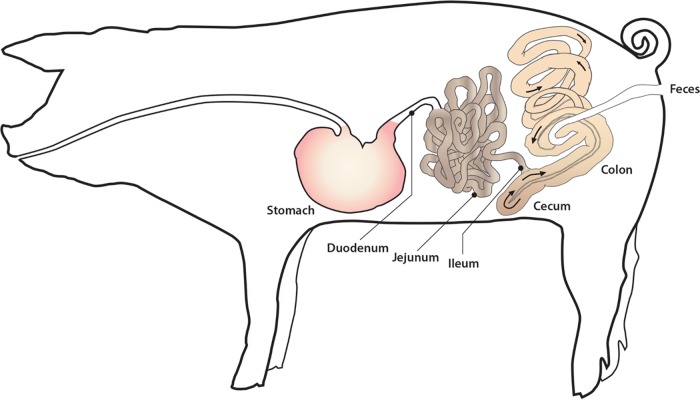
Diagram of the swine gastrointestinal tract with major sections indicated as well as direction of movement of digesta in the colon. Original collection sites are labeled on the drawing.

A total of 25,157,796 quality-filtered 16S rRNA gene sequences from 939 swine gastrointestinal samples were available for meta-analysis (Table 1). Overall, 18,743,484 (74.5%) of these sequences were at least 97% similar to a sequence in the SILVA database. These sequences represented 25,182 bacterial operational taxonomic units (OTUs), 35 phyla, and 887 genera prior to random subsampling. It was necessary to randomly subsample to a level of 1,000 sequences per sample to retain as many samples as possible; however, as few as 100 sequences per sample have been shown to be sufficient when large differences between sample groups are present (19). Not surprisingly, the majority of sequences available for meta-analysis were derived from swine feces, as these samples are the easiest to obtain from the GI tract and allow repeated sampling over time. Other regions of the swine GI tract, such as the ileal and colonic mucosa and digesta, have also been well represented among publically available sequences (Fig. 1). Conversely, the number of samples from the duodenum and jejunum (digesta and mucosa) was relatively small (*n* ≤ 5 samples) and belonged to a single study. Therefore, these samples were not included in the linear discriminant analysis (LDA) effect size (LEfSe) described below. Sequences were also available from three different continents and 10 countries, although approximately half of these were from studies conducted in the United States. The age of the pigs used in each study ranged from~3 weeks (preweaning piglets) to ~24 weeks (slaughter age).

**TABLE 1:** Details of studies included in the meta-analysis

Reference	No. of samples	Hypervariable region(s)	Sequencing platform	Avg % assigned sequences per sample	DNA extraction method	Sample type(s)	Study observation(s)	Age of pigs at sampling	Country(ies) of origin	Data availability (accession no.)
J. Trachsel, B. E. Bass, and H. K. Allen, unpublished	52	V1 to V3	Illumina MiSeq	76.2%	PowerSoil-htp 96-well soil DNA isolation kit	Ileum digesta, fecal	Subset of samples taken before heat stress treatment	7 wks	United States	PRJNA335425
17	24	V1 to V2	Illumina MiSeq	88.9%	NA^b^	Ileum digesta	Pigs challenged with *E. coli* F4+ and fed IgG had a lower relative abundance of *Enterococcaceae* and *Streptococcaceae* and increased ileal diversity	6 wks	Denmark	PRJNA302730
15^a^	18	V4	Illumina MiSeq	75.4%	QIAamp DNA stool minikit	Colon digesta	Pigs fed DDGS^c^ had a different microbial community structure	9 wks	United States	PRJEB11368
45^a^	40	V3 to V4	454 FLX	74.1%	QIAamp DNA stool minikit	Colon digesta, fecal	No differences in the gut microbiota between conventionally and organically raised pigs	24 wks	Denmark, France, Italy, Sweden	PRJNA266576
66	39	V4	Illumina MiSeq	85.2%	PowerFecal DNA isolation kit (MoBio)	Fecal	Difference between the fecal microbiota of black and white breed pigs	3–12 wks	South Korea	PRJNA252973
67	53	V1 to V3	454 FLX	83.3%	QIAamp DNA stool minikit	Colon, ileal digesta	Sow-reared and formula-fed piglets had a different gut microbiota	3 wks	United States	PRJNA266806
13	20	V4	Illumina MiSeq	75.8%	Custom	Ileum, cecum digesta	No effect of high or low amylose/amylopectin ratio diets on the gut microbiota	21 wks	China	PRJNA281781
68	19	V4	Illumina MiSeq	81.3%	PowerFecal DNA isolation kit (MoBio)	Fecal	Differences in fecal microbiota by production phase	Piglets, growers, finishers, sows	South Korea	PRJNA267233
16	14	V3 to V5	Illumina MiSeq	73.2%	PowerSoil DNA isolation kit (MoBio)	Cecum digesta	Minor differences in the cecal microbiota of pigs fed 70% enzymatically modified cornstarch or control starch	14 wks	Austria	PRJEB8696
11	62	V4	Illumina MiSeq	80.6%	PowerFecal DNA isolation kit (MoBio)	Fecal	No effect of diet with 0.2% chlortetracycline, sulfathiazole, and penicillin on the fecal microbiota	3–12 wks	South Korea	PRJNA257009
10	94	V4	Illumina MiSeq	51.9%	ZR fecal DNA MiniPrep (Zymo Research)	Fecal	Subtherapeutic tylosin, but not chlortetracycline, altered the fecal microbiota	3–19 wks	Canada	PRJNA244928
12	44	V1 to V3	454 FLX	87.6%	PowerSoil DNA isolation kit (MoBio)	Ileal mucosa	No effect of in-feed chlortetracycline or high-complexity diets on the ileal mucosa microbiota	6–11 wks	Canada	PRJNA257705
7	80	V1 to V3	454 FLX	62.8%	Ultra-Clean soil DNA isolation kit (MoBio)	Gastric mucosa, duodenum, jejunum, ileum cecum, colon digesta and mucosa, fecal	Reduced diversity in the ileum versus colon, ASP250-altered gut microbiota	15 wks	United States	PRJNA72355
8	101	V1 to V3	454 FLX	54.1%	PowerSoil DNA isolation kit	Fecal	Carbadox resulted in early but not late alterations of the fecal microbiota	6–15 wks	United States	PRJNA237795; SAMN02645017 to SAMN02645066
69	23	V1 to V3	454 FLX	47.3%	Ultra-Clean Fecal DNA isolation kit (MoBio)	Fecal	The fecal microbiota of Landrace pigs differed that of from pigs of the Duroc breed	15 wks	South Korea	PRJNA263439
14	92	V1 to V2	454 FLX	83.2%	PowerSoil DNA isolation kit (MoBio)	Gastric, ileum, colon mucosa	Gut microbiota affected by Ca-P content and diet type	7 wks	Austria	PRJEB1576
70	10	V3	Illumina HiSeq 2000	88.8%	QIAamp DNA stool minikit (Qiagen)	Cecum digesta	Fostering of piglets changes cecal microbiota	3 wks	China	PRJNA214736
18	59	V1 to V3	454 FLX	80.7%	PowerSoil DNA isolation kit (MoBio)	Fecal	Fecal microbiota differed between high- and low-*Salmonella*-shedder pigs	8–11 wks	United States	PRJNA169497
9	12	V3	454 FLX	67.5%	Powermax soil DNA isolation kit (MoBio)	Fecal	In-feed ASP250 altered fecal microbiota	18–20 wks	United States	PRJNA72355; SRP004660
6	83	V1 to V3	454 FLX	44.4%	Custom	Fecal	In-feed ASP250 altered fecal microbiota	3–13 wks	United States	PRJNA72355; SRP008126

a Data are from studies that describe using commercial pigs.

b NA, not available.

c DDGS, distillers’ dried grains with solubles.

### Identifying the core microbiota of the swine gastrointestinal tract.

**FIG 2:**
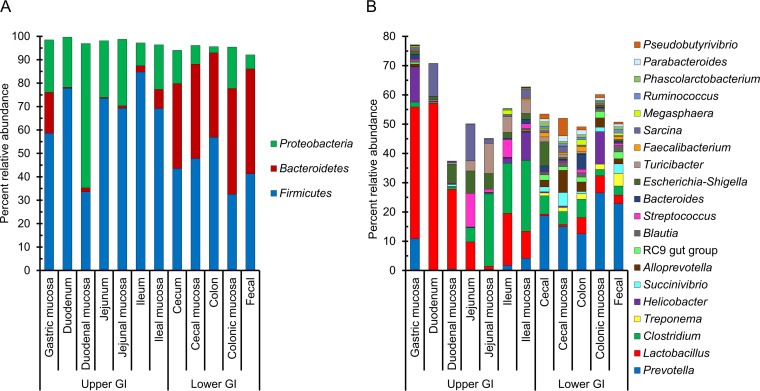
Percent relative abundances of the three most abundant phyla (A) and 20 most abundant genera (B) by gastrointestinal tract sample type.

The idea of defining a core microbiota in the pig gut is intriguing as it may identify potential targets for dietary or therapeutic interventions in the swine production environment. Although a core microbiota may not exist in swine according to a strict definition that requires a particular taxon to be present in each sample, several phyla and genera were found in more than 90% of all GI samples. In the current meta-analysis, the *Firmicutes* and *Bacteroidetes* phyla accounted for nearly 85% of the total 16S rRNA gene sequences among all gastrointestinal locations, with *Proteobacteria* being the only other phylum common to all GI samples (Fig. 2A). No OTUs or genera were shared among all samples analyzed, but the genera *Clostridium*, *Blautia*, *Lactobacillus*, *Prevotella*, *Ruminococcus*, and *Roseburia*, the RC9 gut group, and *Subdoligranulum* were found in more than 90% of samples. *Prevotella* was also the most abundant genus among all genera that were identified (Fig. 2B; see also Table S1 in the supplemental material). In addition, a single OTU classified as an uncultured member of the *Prevotella* was present in at least 75% of all GI samples.

10.1128/mSystems.00004-17.4 TABLE S1  The percentage of samples from each specific gastrointestinal location that had at least one 16S rRNA gene sequence from each of the individual genera identified. Genera are listed in descending order of overall relative abundance. Download TABLE S1, PDF file, 1.1 MB. Copyright © 2017 Holman et al. 2017 Holman et al. This content is distributed under the terms of the Creative Commons Attribution 4.0 International license.

Gut locations and feces yielded discrete core microbiotas. In the fecal microbiota, more than 99% of fecal samples (12 studies) contained sequences from *Prevotella*, *Clostridium*, *Alloprevotella*, *Ruminococcus*, and the RC9 gut group. Certain genera were present in all samples from a particular GI tract location (*n* = >50 samples). These included *Prevotella* and *Blautia* in samples of colonic digesta (*n =* 91; 5 studies); *Anaerovibrio*, *Clostridium*, *Phascolarctobacterium*, *Ruminococcus*, *Sarcina*, and *Streptococcus* in cecal digesta (*n =* 52; 4 studies); and *Clostridium* in ileal mucosal samples (*n =* 91; 3 studies). *Clostridium* spp. were also present in all but one sample of ileal digesta (*n =* 81; 5 studies).

In commercial swine production, pigs are typically fed a cereal grain-based diet that is relatively high in carbohydrate content (20). Meanwhile, *Prevotella* spp. have been reported to be associated with dietary carbohydrates in humans (21, 22), producing polysaccharidases such as glucanase, mannanase, and xylanase (23). Members of *Prevotella* are also relatively abundant in the rumen and/or gut microbiota of other animals such as cattle (24), chimpanzees (25), goats (26), and sheep (27), attesting to the ubiquity of this genus in the mammalian gut. *Clostridium*, *Blautia*, and *Ruminococcus* are all members of the *Clostridiales* order, and, similarly to *Prevotella*, are widely found in the mammalian gut (28). These genera can produce butyrate, a short-chain fatty acid (SCFA), most often from acetate (also an SCFA) via the butyryl-coenzyme A (CoA):acetate CoA-transferase pathway (29). *Prevotella* spp., which produce acetate in the gut, are thereby able to provide a source of energy for butyrate-producing bacteria (8). Importantly, butyrate decreases inflammation in the gut of the host, and cells in the intestinal epithelium can use it as an energy source (30).

The RC9 gut group, which belongs to the *Rikenellaceae* family, was identified in 90.5% of all GI samples and was among the most abundant genera in the lower GI tract (Fig. 2B; Table S1). This finding is noteworthy as its presence in the swine gut microbiota was reported only recently (31, 32). The *Rikenellaceae* family itself is a relatively new taxonomic classification within the *Bacteroidales* order, with only three genera currently described (33). Similarly to *Prevotella*, also a member of the *Bacteroidales*, some bacteria in the *Rikenellaceae* family are acetate producers (34), suggesting a high level of functional redundancy for this metabolite in the swine gut microbiome.

### Factors affecting the swine gut microbiota.

**FIG 3:**
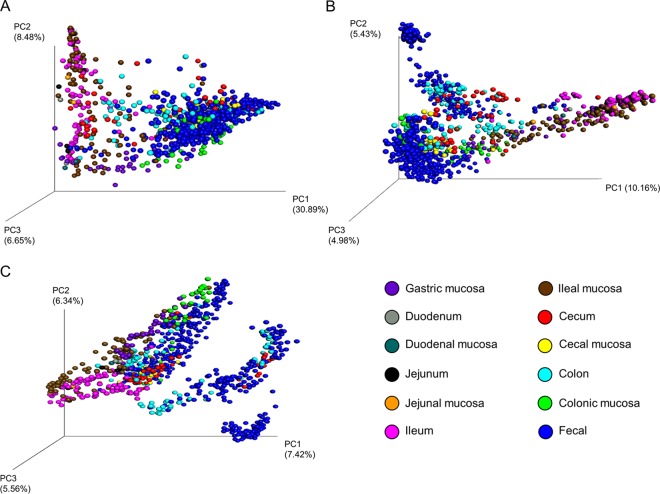
Principal-coordinate analysis plots of weighted UniFrac distances (A), unweighted UniFrac distances (B), and Bray-Curtis dissimilarities (C) classified by gastrointestinal tract sample type. The percentages of variation explained by the principal coordinates are indicated on the axes.

We determined which variables most strongly affected the structure of the swine gut microbiota using a permutational multivariate analysis of variance (PERMANOVA) test of the unweighted and weighted UniFrac distances and Bray-Curtis dissimilarities (Table 2). Among all distance metrics, each metadata category tested was significantly associated with the microbial community structure. However, this finding is likely a result of the heterogeneity of dispersion within each group in each metadata category, as shown by the permutational analysis of multivariate dispersions (PERMDISP; *P* < 0.05). Overall, “study” was the most important factor affecting the swine gut microbiota, as it explained the greatest variation in the data set (Fig. 3; also see Fig. S1 in the supplemental material). The GI location that was sampled was also strongly associated with the structure of the microbiota as assessed using weighted UniFrac distances (Fig. 3A); however, this finding was not replicated in the results obtained with respect to the unweighted UniFrac distances and Bray-Curtis dissimilarities (Fig. 3B and C). Therefore, researchers could reasonably expect that the taxa that are relatively abundant in their swine gut samples are similar to those in samples taken from the same GI location of different pigs compared with those found in samples obtained from different GI locations.

10.1128/mSystems.00004-17.1 FIG S1  Principal-coordinate analysis plots of Bray-Curtis dissimilarities by study. The numbers in parentheses in the figure legend refer to the number of samples included for each study. The percentages of variation explained by the principal coordinates are indicated on the axes. Download FIG S1, TIF file, 2.1 MB. Copyright © 2017 Holman et al. 2017 Holman et al. This content is distributed under the terms of the Creative Commons Attribution 4.0 International license.

**TABLE 2:** Factors associated with the community structure of the swine gut microbiota as measured using PERMANOVA with the adonis function (9,999 permutations) of the weighted and unweighted UniFrac distances and Bray-Curtis dissimilarities^a^

Parameter	Value								
	Weighted UniFrac			Unweighted UniFrac			Bray-Curtis		
	Pseudo-F ratio	*R*^2^	*P* value	Pseudo-F ratio	*R*^2^	*P* value	Pseudo-F ratio	*R*^2^	*P* value
Study	28.2	0.37	0.0001	18.6	0.28	0.0001	24.4	0.34	0.0001
GI sampling location	35.4	0.29	0.0001	12.9	0.13	0.0001	12.1	0.13	0.0001
Age	13.9	0.21	0.0001	8.5	0.14	0.0001	10.6	0.16	0.0001
Country of origin	25.4	0.18	0.0001	15.6	0.12	0.0001	20.4	0.15	0.0001
Hypervariable region sequenced	34.5	0.16	0.0001	22.7	0.11	0.0001	18.9	0.13	0.0001
Sequencing platform	20.4	0.04	0.0001	19.0	0.04	0.0001	25.6	0.05	0.0001

a For the age category, one study was excluded due to a lack of information about the age of the pigs used. PERMANOVA, permutational multivariate analysis of variance.

We evaluated the effect of age on the swine gut microbiota using samples from the 19 studies (of 20) that provided specific information for pig age (*n =* 920; Table 2; Fig. S2). Although the effect of the age of the pigs sampled was not as large as the effects of study and GI sampling location, it did explain more variation in the data set than the remaining three metadata categories for the weighted UniFrac distances. It should be noted that pig age, like several of the other metadata categories, is also associated with “study,” as many studies sampled from pigs at only one particular time point. In addition, the 3-week-old category included both preweaned and postweaned pigs, and weaning is known to result in significant changes to the swine gut microbiota (10, 35).

10.1128/mSystems.00004-17.2 FIG S2  Principal-coordinate analysis plot of the weighted UniFrac distances for all samples with associated metadata for the age of the pigs used in the study. Samples are colored by pig age in weeks. The numbers in parentheses for the figure legend refer to the number of samples at each week. The percentages of variation explained by the principal coordinates are indicated on the axes. Download FIG S2, TIF file, 2.5 MB. Copyright © 2017 Holman et al. 2017 Holman et al. This content is distributed under the terms of the Creative Commons Attribution 4.0 International license.

**FIG 4:**
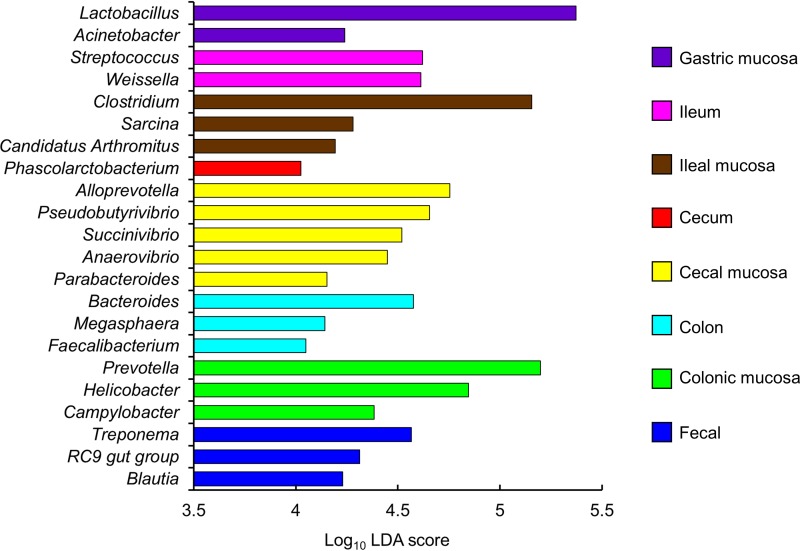
Differentially abundant genera in each gastrointestinal tract sample type as assessed using linear discriminant analysis (LDA) with effect size (LEfSe) measurements. Only those genera with an LDA score (log_10_) of >4.0 are displayed. Samples of duodenum and jejunum mucosa and digesta were excluded from analysis as there were fewer than five samples for each.

Genera that were enriched in specific GI samples were identified using linear discriminant analysis (LDA) effect size (LEfSe) (Fig. 4). Among the more notable genera, *Lactobacillus* was more abundant in the gastric mucosa, *Prevotella*, *Helicobacter*, and *Campylobacter* in the colonic mucosa, *Clostridium* in the ileal mucosa, *Alloprevotella* in the cecal mucosa, *Bacteroides* in the colon, and *Treponema* in fecal samples. These differences in the relative abundances of certain genera along the gastrointestinal tract likely reflect the physiological conditions at each section. For example, the pH is significantly lower in the stomach than at any other GI location and *Lactobacillus* spp. are relatively acid tolerant, possibly explaining their enrichment in this location (36). The lactobacilli are also able to attach to the epithelial and mucosal layers, where they can form biofilm-like communities, thereby aiding their persistence (37). *Lactobacillus* spp. were present among all types of GI samples; therefore, the population of lactobacilli in the stomach may serve as a source of these bacteria for other gut locations (38).

Comparisons of alpha-diversity measures also revealed significant differences among samples from different GI locations, including feces (Table 3). In particular, there was a clear demarcation between upper and lower GI samples, with significantly higher diversity, richness, and evenness in the lower GI samples (*P* < 0.05). Within the lower GI tract, fecal and cecal mucosa samples were the most rich in terms of the number of OTUs and phylogenetic diversity (PD). These observations are similar to those reported in the longitudinal swine gut microbiota study conducted by Looft et al. (7). The only significant difference among upper GI tract sections was observed between the samples of ileal digesta and mucosa, where the mucosal samples had greater phylogenetic diversity as determined using PD whole tree. These results are expected, given that transit is faster in the upper GI tract than in the lower GI tract, thereby limiting adhesion and colonization of bacteria in the upper GI tract (39). Also, competition for nutrients between the host and bacteria is greater in the upper GI tract (40).

**TABLE 3:** Alpha-diversity measures for each gastrointestinal location^a^

GI sample type	Values				
	No. of OTUs	Phylogenetic diversity	Shannon index	Simpson’s reciprocal index	Equitability
Gastric mucosa (*n* = 36)	135 ± 66c	26.9 ± 12.8c	3.04 ± 1.02d	12 ± 10.2b	0.62 ± 0.15cd
Duodenum (*n* = 3)	92 ± 13c	14 ± 1.7d	1.87 ± 0.98d	3.7 ± 3.7b	0.41 ± 0.21d
Duodenal mucosa (*n* = 2)	120 ± 0c	23.2 ± 2.7cd	2.85 ± 0.02d	6.4 ± 0.3b	0.59 ± 0.00cd
Jejunum (*n* = 4)	118 ± 16c	18.2 ± 4.2cd	3.06 ± 0.40cd	9.4 ± 4.3b	0.64 ± 0.07bcd
Jejunal mucosa (*n* = 3)	126 ± 25c	21.4 ± 7.2cd	3.22 ± 0.27bcd	11.4 ± 2.7b	0.67 ± 0.03abc
Ileum (*n* = 81)	94 ± 42c	15.8 ± 6.3d	2.72 ± 0.89d	10.2 ± 8.3b	0.60 ± 0.14cd
Ileal mucosa (*n* = 91)	128 ± 54c	24.1 ± 9.6c	3.02 ± 0.86d	11.5 ± 9.8b	0.62 ± 0.14cd
Cecum (*n* = 52)	213 ± 81b	35.7 ± 10.3b	4.02 ± 0.83bc	30.3 ± 24.5a	0.75 ± 0.11b
Cecal mucosa (*n* = 18)	282 ± 51a	42.9 ± 7.3ab	4.61 ± 0.43a	42.8 ± 18.5a	0.82 ± 0.05a
Colon (*n* = 91)	221 ± 66b	37.7 ± 9b	4.23 ± 0.53ab	33.2 ± 22.6a	0.79 ± 0.06ab
Colonic mucosa (*n* = 48)	210 ± 90b	37.2 ± 12b	4.05 ± 0.74abc	30.9 ± 26.9a	0.76 ± 0.09ab
Fecal (*n* = 510)	255 ± 73a	44.1 ± 11.7a	4.41 ± 0.65a	40.6 ± 29.2a	0.80 ± 0.08a

a Different lowercase letters in each column indicate significant differences (*P* < 0.05). Data represent means ± standard deviations.

Although it is reassuring that GI sampling location is a strong predictor of the swine gut microbial community structure, study-level effects are also significant, as evidenced above. The importance of technical variation among studies has been documented in recent meta-analyses of the avian gut microbiota (4) and the indoor microbiota (5), where study was the most important driver of the microbiota. In the present meta-analysis, a wide range of ages were included for the pigs, as well as different diets, pig breeds, and administered treatments (Table 1). In addition, at least seven different DNA extraction methods were used and six separate 16S rRNA gene hypervariable regions were sequenced on three different sequencing platforms. The use of standard or uniform DNA extraction, 16S rRNA gene library preparation, and sequencing protocols would likely help reduce some of this study-to-study variation. With that said, eliminating all sources of technical variation in a single study may prove difficult as even technical replicates from different sequencing centers can differ significantly (41).

### Effect of 16S rRNA gene hypervariable region used for sequencing.

**FIG 5:**
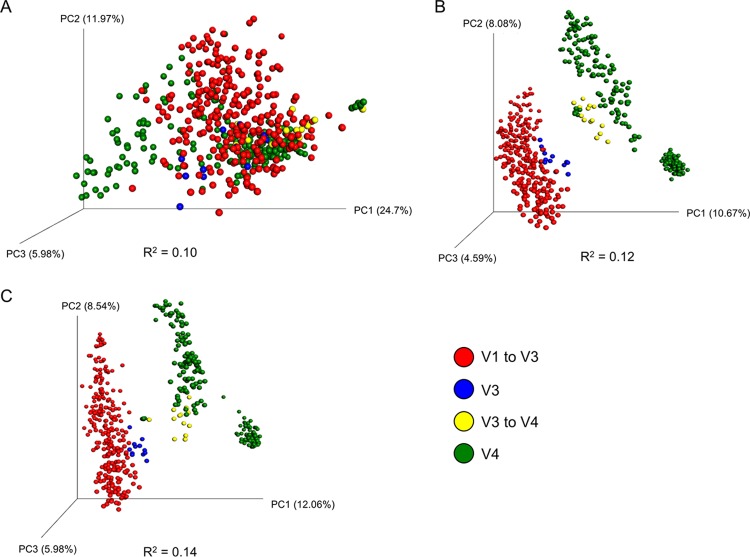
Principal-coordinate analysis plots of weighted UniFrac distances (A), unweighted UniFrac distances (B), and Bray-Curtis dissimilarities (C) classified by hypervariable region sequenced for fecal samples only. Percentages of variation explained by the principal coordinates are indicated on the axes, and the *R* values on each plot indicate the dissimilarities between the hypervariable region groups.

The choice of which 16S rRNA gene hypervariable region(s) to sequence is an important consideration for any 16S-based microbial community study as even so-called “universal primers” differ greatly in their amplification of certain bacterial groups (42). To determine the effect of different 16S hypervariable region sequences on the characterization of the swine gut microbiota, we analyzed only fecal samples to eliminate one of the largest sources of variability among samples (i.e., the GI sample type). When unweighted and weighted UniFrac distances and Bray-Curtis dissimilarities were plotted using PCoA, samples clustered by which hypervariable region was sequenced, although the amount of variation explained was smaller for the weighted UniFrac distances and there was more overlap between samples sequenced with different hypervariable regions (Fig. 5). In the fecal samples, the study effect was still stronger than the effect of the hypervariable region sequenced with PERMANOVA (adonis *R*^2^ values of 0.31, 0.28, and 0.36 for weighted and unweighted UniFrac distances and Bray-Curtis dissimilarities, respectively). However, this is to be expected given that each study sequenced only one specific 16S rRNA gene hypervariable region. Fecal samples that were sequenced using primers that targeted the V4 region did have more phylogenetic diversity (PD whole tree) and a higher Simpson’s reciprocal value than those that were sequenced using primers that targeted the other 16S hypervariable regions analyzed, although the richness and the Shannon index data were not significantly different (Table 4).

**TABLE 4:** Alpha-diversity measures by hypervariable region sequenced for fecal samples only^a^

Hypervariable region(s)	Value(s)				
	No. of OTUs	Phylogenetic diversity	Shannon index	Simpson’s reciprocal index	Equitability
V1 to V3 (*n* = 270)	253 ± 49	42.6 ± 6.9a	4.46 ± 0.44	37.6 ± 23a	0.81 ± 0.06a
V3 (*n* = 12)	232 ± 35	42.1 ± 5.7a	4.29 ± 0.4	29 ± 12.5a	0.79 ± 0.06b
V3 and V4 (*n* = 14)	236 ± 50	39.6 ± 7.1a	4.26 ± 0.69	30.3 ± 17.5a	0.78 ± 0.1b
V4 (*n* = 214)	259 ± 97	46.3 ± 15.9b	4.35 ± 0.85	45.7 ± 36.1b	0.79 ± 0.1b

a Different lowercase letters in each column indicate significant differences. Data represent means ± standard deviations.

Comparing the region V1 to V3 and region V4 sequences from fecal samples using LEfSe, the region V1 to V3 sequences contained a higher proportion of the *Firmicutes* phylum and *Blautia* genus (LDA score [log_10_] = >4.0). Sequences classified at the phylum level as *Spirochaetae* and at the genus level as *Bacteroides*, *Lactobacillus*, and *Treponema* were more abundant in fecal samples that were sequenced using the V4 region. Another notable difference at the genus level among the different hypervariable regions was the nearly complete absence of *Bifidobacterium* in fecal samples from studies using the region V1 to V3 and region V3 16S primer sets (1 sample of 288; Table S2). In contrast, 74.3% of fecal samples that were sequenced using the V4 hypervariable region and 93% of fecal samples using region V3 to V4 had at least one *Bifidobacterium* 16S rRNA gene sequence. The generic 27F primer sequence (5′-AGAGTTTGATCCTGGCTCAG-3′) which is often used to amplify regions V1 to V3 has been previously reported to underestimate the relative abundance of bifidobacteria unless modifications are made to the primer sequence (43, 44). Although the relative abundance of *Bifidobacterium* in fecal samples remained low (<0.35%) in the present meta-analysis, this may be an important consideration for researchers interested in this particular genus.

10.1128/mSystems.00004-17.5 TABLE S2  The percentage of fecal samples grouped by specific 16S rRNA gene hypervariable region sequenced that had at least one 16S rRNA gene sequence from each of the individual genera identified. Genera are listed in descending order of overall relative abundance. Download TABLE S2, PDF file, 0.5 MB. Copyright © 2017 Holman et al. 2017 Holman et al. This content is distributed under the terms of the Creative Commons Attribution 4.0 International license.

Primers targeting the V4 region, however, poorly amplify sequences from *Spirochaeta* and *Desulfitibacter*, as these genera were identified in only 12.1% and 0.5% of fecal samples that were sequenced with V4 primers, compared to 91.5% and 77.8% of fecal samples analyzed with region V1 to V3 primers, respectively (Table S2). Although only 14 fecal samples from a single study were available for analysis of region V3 to V4 (45), *Treponema* spp. were completely absent from these samples, an unexpected finding given the ubiquity of this genus among fecal samples sequenced using other 16S primers; therefore, those results may reflect the primer set used rather than the hypervariable region sequenced.

### Studies including antimicrobial administration.

Antimicrobials are frequently used in swine production for the purpose of growth promotion, disease prevention, or disease treatment (46). However, their usage in pigs may help maintain a reservoir of antimicrobial-resistant bacteria and resistance determinants (47, 48). The development of alternatives to antimicrobials in swine requires a better understanding of how the microbiota is affected by these agents (49, 50). As 16S rRNA gene sequences were available from six separate studies that investigated the effect of antimicrobial use on the swine fecal microbiota, we combined these studies to determine if any pattern would emerge that was associated with the use of any type of antimicrobial agent in swine (6–11). Samples once again clustered by study rather than by antimicrobial use, as nonantimicrobial control samples were not separated from antimicrobial treatment samples within each study (Fig. S3). Also, no genera were significantly different between pigs that received antimicrobials and those that did not (LDA [log_10_] score, <4.0). This result is perhaps not surprising given that there is a strong study effect and that antimicrobials with different mechanisms of action and dosages were used in each study. Each study also reported findings that are not necessarily in agreement with each other (51). For example, the *Bacteroidetes* phylum has been reported to be relatively less abundant at specific time points in pigs fed tylosin (10) and ASP250 (49). However, carbadox treatment has been shown to actually increase the proportion of *Bacteroidetes* during the early phase of administration (8).

10.1128/mSystems.00004-17.3 FIG S3  Principal-coordinate analysis plots of weighted UniFrac distances (A), unweighted UniFrac distances (B), and Bray-Curtis dissimilarities (C) for studies investigating the effect of an antimicrobial on the swine fecal microbiota. Samples are colored by study and antimicrobial treatment. Download FIG S3, TIF file, 1.7 MB. Copyright © 2017 Holman et al. 2017 Holman et al. This content is distributed under the terms of the Creative Commons Attribution 4.0 International license.

These findings emphasize the need for additional research into the effects of antimicrobials on the swine gut microbiota to better predict the response of the microbiota to these agents and to aid in the development of alternatives. The use of larger numbers of animals, longitudinal sampling, and the reporting of pig mass and other production attributes in each trial would all improve our understanding of the effect that antimicrobial agents have on gut microbiota of swine. It is also possible that any beneficial health or production effects observed in the pig are a result of changes in areas of the GI tract that preclude repeated sampling such as the ileum or cecum or that directly impact the host animal rather than the microbiota.

### Conclusions.

The results from our meta-analysis demonstrate that after “study” effects, GI tract location is the strongest predictor of the swine gut microbiota composition. In particular, the relatively abundant taxa are similar among samples from the same GI location, independently of other experimental variables. However, technical variation between individual studies may mask other biological differences among swine gut samples. For example, it was not possible to identify any consistent patterns related to antimicrobial use when six studies that included an antimicrobial treatment were analyzed together. The specificity of 16S rRNA gene primers and the hypervariable region that is sequenced also have a large effect on the analysis of the microbiota, as demonstrated in fecal samples from studies using different 16S regions. Nonetheless, a portion of the gut microbiota is shared among at least 90% of GI samples regardless of experimental variables, and this includes *Clostridium*, *Blautia*, *Lactobacillus*, *Prevotella*, *Ruminococcus*, *Roseburia*, the RC9 gut group, and *Subdoligranulum* at the genus level. These genera represent bacteria that are well adapted to the swine gut and may serve as potential markers of a typical swine gut microbiota. The inclusion of comprehensive metadata to accompany sequencing data sets would enhance future meta-analyses and allow researchers to compare their results directly with those from similar studies.

## MATERIALS AND METHODS

### Data acquisition and study inclusion criteria.

A total of 20 studies were included in the meta-analysis and are described in Table 1. Studies were identified through a literature search of the Short Read Archive (SRA; http://www.ncbi.nlm.nih.gov/sra), NCBI PubMed (http://www.ncbi.nlm.nih.gov/pubmed), and Google Scholar (http://scholar.google.com). To be included in the meta-analysis, all studies were required (i) to have been sampled from the swine GI tract, (ii) to have used high-throughput sequencing of the 16S rRNA gene, (iii) to have associated metadata and quality score files, and (iv) to have provided data that were publically available before 31 March 2016. Sequence files from each study were downloaded from the SRA or European Nucleotide Archive (ENA; http://www.ebi.ac.uk/ena).

### Processing of 16S rRNA gene sequences.

All downloaded 16S rRNA gene sequences were processed using the QIIME software package (v. 1.9.1) (52). When paired-end sequence files were provided from Illumina MiSeq or HiSeq 2000-based studies, reads were joined using fastq-join (53) with an allowed maximum difference of 15% and a minimum overlap of 35 bp. Sequences derived from the 454 FLX platform were converted from the fastq file format to fasta and qual files, and sequences were subsequently quality filtered using the *split_libraries.py* command with a Phred score cutoff of 25, a minimum read length of 200 bp, and a maximum homopolymer run of 6 bp. Illumina MiSeq and HiSeq 2000 reads were quality filtered using the *split_libraries_fastq.py* command with a Phred score cutoff of 25, reads were truncated following three consecutive base calls with a Phred score of <25, and reads with <75% of the original read length following truncation were discarded. The maximum length of each read was set based on the expected read length, as reads spanning regions V1 to V3, for example, are significantly longer than those corresponding to the V4 region.

The quality-filtered 16S rRNA gene sequences were clustered into operational taxonomic units (OTUs) at 97% similarity using a closed-reference approach, the SILVA release 119 database (54), and USEARCH v. 6.1.544 (55), with reverse strand matching enabled. In this method, reads that failed to match one of the 16S rRNA gene sequences in the SILVA database at 97% similarity were discarded. Depending upon the hypervariable region targeted, some 16S rRNA gene primers also amplify archaeal 16S rRNA gene sequences. Therefore, all sequences classified as *Archaea*, as well as chloroplast, mitochondrial, and eukaryotic sequences, were removed prior to downstream analysis. All samples were randomly subsampled to a level of 1,000 sequences per sample to account for uneven sequencing depth among samples and studies.

Samples were categorized based on sample type, treatment, diet, country of origin, age, sequencing platform, and 16S rRNA hypervariable region sequenced, where available. Only information about sample type, country of origin, sequencing platform, and hypervariable region sequenced was available for each sample. Linear discriminant analysis effect size (LEfSe) was used to determine which genera were significantly enriched in each sample type. Genera that were relatively more abundant in a particular sample group were identified by LEfSe using the Kruskal-Wallis test (*P* < 0.05), and the effect size of each of these genera was estimated using linear discriminant analysis (56). An LDA score (log_10_) of 4.0 was used as the cutoff for identifying differentially abundant genera.

The between-sample (beta) diversity was assessed using the unweighted and weighted UniFrac (57) distances and Bray-Curtis (58) dissimilarities. Principal-coordinate analysis (PCoA) was used to visualize these distances using Emperor (59). Permutational multivariate analysis of variance (PERMANOVA) using the adonis function (60) with 9,999 permutations was implemented in QIIME to analyze the unweighted and weighted UniFrac distances and the Bray-Curtis dissimilarities for each gastrointestinal location, country of origin, hypervariable region, sequencing platform used, and study. Pig age was also assessed but with one study removed due to a lack of metadata. Permutational analysis of multivariate dispersions (PERMDISP) was used to test the homogeneity of dispersion for each metadata category as implemented in QIIME using the betadisper function of vegan (60). The within-sample (alpha) diversity, richness, and evenness were calculated within QIIME using the Shannon index (61), phylogenetic diversity (PD whole tree) (62), Simpson reciprocal index (63), and equitability (evenness) index. These metrics, as well as the metric of hypervariable region sequenced for fecal samples only, were compared among gastrointestinal sample types using a two-way ANOVA in R (v. 3.2.5) (64) with hypervariable region and sample type as the independent factors, followed by Tukey’s honestly significant difference (HSD) *post hoc* pairwise comparison test (agricolae package [65]). All results were considered significant at *P* values of <0.05.
