# Supplementary material for: Meta-analysis To Define a Core Microbiota in the Swine Gut
Source: mSystems. 2017 May 23;2(3):e00004-17. doi: 10.1128/mSystems.00004-17 (PMC5443231; doi:10.1128/mSystems.00004-17)
Supplement: TABLE S1 [file sys003172103st4.pdf]

TABLE S1. The percentage of samples from each specific gastrointestinal location that had at least one 16S rRNA gene sequence from each of the individual genera identified. Genera are listed in descending order by overall relative abundance. The number of GI samples from each sample type is indicated in parentheses. The overall relative abundance is listed as the percent mean ± standard deviation among all samples.

Genus	Gastric mucosa (n = 36; 2 studies)	Duodenum (n = 3; 1 study)	Duodenum mucosa (n = 2; 1 study)	Jejunum (n = 4; 1 study)	Jejunal mucosa (n = 3; 1 study)	Ileum (n = 81; 6 studies)	Ileal mucosa (n = 91; 4 studies)	Cecum (n = 52; 5 studies)	Cecal mucosa (n = 18; 2 studies)	Colon (n = 91; 5 studies)	Colonic mucosa (n = 48; 3 studies)	Fecal (n = 510; 12 studies)	Overall percent relative abundance
Prevotella	97.2	100.0	100.0	25.0	100.0	59.3	97.8	78.8	100.0	100.0	100.0	99.6	17.284 ± 17.494
Lactobacillus	100.0	100.0	100.0	100.0	66.7	96.3	97.8	94.2	83.3	96.7	95.8	94.3	6.922 ± 16.002
Clostridium	88.9	100.0	100.0	100.0	100.0	98.8	100.0	100.0	100.0	97.8	91.7	99.2	6.812 ± 12.789
Treponema	77.8	66.7	50.0	50.0	33.3	11.1	63.7	75.0	100.0	42.9	97.9	93.9	2.729 ± 6.303
Helicobacter	72.2	33.3	50.0	0.0	100.0	30.9	52.7	65.4	88.9	40.7	100.0	56.9	2.156 ± 10.066
Succinivibrio	55.6	0.0	100.0	0.0	66.7	14.8	37.4	86.5	100.0	53.8	77.1	94.7	2.064 ± 4.904
Alloprevotella	86.1	0.0	50.0	0.0	66.7	32.1	82.4	86.5	100.0	97.8	100.0	99.2	1.773 ± 2.531
RC9 gut group	88.9	66.7	100.0	50.0	66.7	44.4	80.2	98.1	100.0	86.8	100.0	99.0	1.728 ± 2.055
Blautia	94.4	33.3	50.0	75.0	100.0	87.7	95.6	96.2	100.0	100.0	97.9	98.8	1.522 ± 1.712
Streptococcus	100.0	100.0	100.0	100.0	100.0	95.1	94.5	100.0	94.4	93.4	52.1	83.3	1.346 ± 4.059
Bacteroides	63.9	33.3	0.0	25.0	33.3	35.8	60.4	84.6	94.4	86.8	93.8	85.9	1.264 ± 4.49
Escherichia-Shigella	69.4	100.0	100.0	100.0	100.0	91.4	92.3	90.4	66.7	61.5	60.4	63.3	1.228 ± 5.099
Turicibacter	50.0	66.7	100.0	100.0	100.0	87.7	75.8	90.4	100.0	68.1	41.7	53.7	1.219 ± 4.391
Faecalibacterium	72.2	0.0	50.0	0.0	33.3	40.7	81.3	96.2	100.0	97.8	97.9	96.1	0.892 ± 2.089
Sarcina	52.8	100.0	100.0	100.0	100.0	75.3	84.6	100.0	100.0	87.9	85.4	93.3	0.844 ± 3.404
Megasphaera	80.6	0.0	50.0	0.0	0.0	43.2	78.0	67.3	44.4	75.8	75.0	81.8	0.84 ± 2.434
Ruminococcus	80.6	0.0	0.0	25.0	33.3	71.6	85.7	100.0	100.0	98.9	97.9	99.0	0.829 ± 0.861
Phascolarctobacterium	77.8	33.3	50.0	0.0	66.7	46.9	69.2	100.0	100.0	95.6	75.0	97.5	0.801 ± 1.065
Parabacteroides	63.9	0.0	0.0	0.0	33.3	25.9	51.6	82.7	100.0	93.4	97.9	96.7	0.784 ± 1.361
Pseudobutyrivibrio	72.2	0.0	0.0	25.0	66.7	19.8	83.5	90.4	100.0	91.2	97.9	97.8	0.782 ± 1.588
Weissella	27.8	100.0	100.0	100.0	33.3	43.2	34.1	44.2	44.4	31.9	20.8	9.0	0.728 ± 5.685
Roseburia	77.8	0.0	50.0	25.0	33.3	54.3	83.5	98.1	100.0	96.7	100.0	98.0	0.71 ± 1.299
Anaerovibrio	63.9	0.0	50.0	0.0	33.3	18.5	65.9	100.0	100.0	73.6	91.7	94.1	0.635 ± 1.244
Campylobacter	55.6	33.3	50.0	25.0	0.0	27.2	72.5	69.2	50.0	49.5	81.3	71.6	0.573 ± 2.404
Coprococcus	61.1	0.0	50.0	25.0	66.7	32.1	79.1	98.1	100.0	95.6	95.8	97.5	0.422 ± 0.627
Acinetobacter	88.9	100.0	100.0	100.0	100.0	32.1	48.4	38.5	38.9	8.8	10.4	20.4	0.415 ± 3.198
Subdoligranulum	61.1	0.0	0.0	50.0	66.7	65.4	83.5	98.1	100.0	94.5	87.5	97.5	0.39 ± 0.567
Catenibacterium	61.1	0.0	0.0	0.0	0.0	27.2	58.2	51.9	33.3	26.4	58.3	70.0	0.372 ± 1.18
Oscillibacter	50.0	0.0	50.0	0.0	0.0	17.3	26.4	96.2	88.9	91.2	83.3	96.9	0.358 ± 0.537
Actinobacillus	88.9	66.7	50.0	100.0	66.7	67.9	72.5	67.3	22.2	25.3	16.7	17.8	0.343 ± 2.054
Dialister	77.8	0.0	0.0	0.0	0.0	50.6	70.3	84.6	88.9	81.3	97.9	66.7	0.331 ± 1.011
Spirochaeta	44.4	0.0	50.0	0.0	33.3	3.7	33.0	96.2	88.9	39.6	91.7	55.9	0.311 ± 0.827
Anaerotruncus	30.6	0.0	50.0	0.0	33.3	9.9	50.5	98.1	100.0	97.8	77.1	94.3	0.297 ± 0.65
Desulfitibacter	58.3	33.3	50.0	100.0	100.0	55.6	80.2	36.5	77.8	31.9	66.7	42.5	0.273 ± 0.714
Selenomonas	58.3	0.0	50.0	0.0	0.0	29.6	57.1	53.8	27.8	53.8	54.2	77.3	0.27 ± 1.2
Candidatus Arthromitus	0.0	0.0	0.0	0.0	0.0	6.2	42.9	0.0	0.0	3.3	0.0	1.2	0.227 ± 2.403
Pseudomonas	83.3	100.0	50.0	75.0	66.7	58.0	87.9	71.2	66.7	33.0	62.5	44.1	0.208 ± 0.835
Oribacterium	52.8	0.0	0.0	25.0	0.0	2.5	44.0	92.3	100.0	63.7	89.6	79.6	0.206 ± 0.71
Pantoea	61.1	66.7	50.0	75.0	66.7	55.6	45.1	44.2	16.7	13.2	22.9	3.3	0.205 ± 1.704
Mitsuokella	69.4	0.0	0.0	0.0	0.0	44.4	58.2	38.5	33.3	51.6	52.1	53.1	0.205 ± 1.065
Arthrobacter	36.1	0.0	0.0	75.0	100.0	91.4	76.9	55.8	61.1	28.6	29.2	29.6	0.205 ± 1.256
Acetitomaculum	16.7	0.0	0.0	50.0	0.0	1.2	19.8	78.8	100.0	39.6	56.3	74.5	0.204 ± 0.726
Incertae Sedis	55.6	0.0	0.0	25.0	66.7	38.3	64.8	61.5	61.1	63.7	79.2	78.2	0.191 ± 0.964
Desulfovibrio	27.8	33.3	50.0	50.0	33.3	17.3	27.5	69.2	16.7	67.0	60.4	69.2	0.187 ± 0.601
dgA-11 gut group	38.9	0.0	50.0	0.0	0.0	0.0	25.3	75.0	61.1	67.0	81.3	77.8	0.186 ± 0.388
Solobacterium	66.7	0.0	50.0	50.0	33.3	46.9	54.9	61.5	100.0	56.0	68.8	89.6	0.177 ± 0.348
Oscillospira	27.8	0.0	0.0	0.0	0.0	1.2	14.3	80.8	88.9	86.8	60.4	79.2	0.169 ± 0.398
Marvinbryantia	61.1	0.0	100.0	50.0	0.0	2.5	58.2	75.0	100.0	76.9	93.8	94.1	0.166 ± 0.238
Fusobacterium	52.8	33.3	0.0	50.0	0.0	16.0	51.6	61.5	83.3	49.5	68.8	43.9	0.154 ± 0.717
Rhodococcus	50.0	100.0	100.0	100.0	100.0	53.1	72.5	55.8	44.4	17.6	33.3	18.4	0.142 ± 1.82
Sutterella	50.0	0.0	0.0	0.0	33.3	18.5	34.1	96.2	100.0	61.5	81.3	87.1	0.14 ± 0.278
Enterobacter	63.9	100.0	100.0	100.0	100.0	55.6	74.7	76.9	44.4	26.4	39.6	38.2	0.139 ± 0.625
Haemophilus	63.9	66.7	50.0	75.0	33.3	46.9	51.6	59.6	0.0	15.4	22.9	8.2	0.133 ± 0.8
Anaerostipes	61.1	0.0	0.0	0.0	0.0	11.1	51.6	82.7	94.4	53.8	77.1	78.8	0.113 ± 0.237
Candidatus Saccharimonas	19.4	0.0	0.0	25.0	33.3	3.7	7.7	48.1	72.2	41.8	64.6	35.3	0.108 ± 0.719
Fibrobacter	13.9	0.0	0.0	0.0	33.3	23.5	27.5	50.0	44.4	35.2	27.1	52.7	0.103 ± 0.295
p-1088-a5 gut group	2.8	0.0	0.0	0.0	0.0	2.5	7.7	30.8	38.9	36.3	31.3	52.7	0.097 ± 0.299
Veillonella	75.0	100.0	50.0	100.0	33.3	65.4	60.4	50.0	0.0	28.6	0.0	11.8	0.094 ± 1.054
Psychrobacter	5.6	0.0	0.0	0.0	0.0	1.2	8.8	3.8	0.0	0.0	2.1	2.7	0.088 ± 2.509
Asteroleplasma	44.4	0.0	0.0	0.0	0.0	7.4	27.5	21.2	22.2	8.8	45.8	19.4	0.085 ± 0.485
Butyricimonas	2.8	0.0	0.0	0.0	0.0	6.2	1.1	21.2	0.0	33.0	0.0	23.3	0.079 ± 0.366
Collinsella	38.9	0.0	0.0	25.0	0.0	38.3	56.0	82.7	50.0	68.1	37.5	87.5	0.078 ± 0.181
Enterococcus	33.3	66.7	0.0	75.0	0.0	34.6	27.5	48.1	0.0	11.0	14.6	27.8	0.075 ± 0.627
Bifidobacterium	8.3	0.0	0.0	0.0	0.0	21.0	2.2	38.5	0.0	36.3	0.0	33.9	0.073 ± 0.329
Alistipes	11.1	66.7	50.0	50.0	0.0	7.4	8.8	48.1	22.2	48.4	12.5	38.6	0.071 ± 0.281
Lachnospira	8.3	0.0	0.0	25.0	0.0	12.3	26.4	59.6	77.8	67.0	37.5	61.4	0.068 ± 0.176
Anaeroplasma	0.0	0.0	0.0	0.0	0.0	0.0	7.7	44.2	61.1	18.7	20.8	39.0	0.065 ± 0.512

Atopobium	22.2	0.0	0.0	0.0	0.0	38.3	7.7	17.3	0.0	12.1	2.1	12.7	0.065 ± 0.679
Elusimicrobium	0.0	0.0	0.0	0.0	0.0	1.2	1.1	28.8	0.0	9.9	2.1	27.8	0.064 ± 0.285
Acidaminococcus	63.9	0.0	0.0	25.0	0.0	32.1	35.2	28.8	0.0	30.8	37.5	31.4	0.061 ± 0.221
Mogibacterium	41.7	0.0	0.0	50.0	0.0	18.5	24.2	38.5	27.8	80.2	47.9	72.4	0.061 ± 0.153
Novosphingobium	52.8	0.0	0.0	75.0	0.0	12.3	26.4	9.6	0.0	0.0	6.3	1.2	0.06 ± 0.761
Thalassospira	2.8	0.0	0.0	0.0	0.0	0.0	12.1	51.9	44.4	39.6	18.8	41.0	0.056 ± 0.228
Halomonas	0.0	0.0	0.0	0.0	0.0	12.3	0.0	19.2	0.0	0.0	0.0	0.6	0.055 ± 0.6
Dorea	41.7	0.0	0.0	25.0	0.0	1.2	25.3	78.8	61.1	74.7	70.8	74.9	0.054 ± 0.112
Leuconostoc	33.3	100.0	100.0	100.0	0.0	32.1	9.9	19.2	0.0	9.9	0.0	4.1	0.051 ± 0.888
Akkermansia	2.8	0.0	0.0	25.0	33.3	19.8	20.9	44.2	50.0	15.4	14.6	31.6	0.051 ± 0.289
Ruminobacter	0.0	0.0	0.0	0.0	0.0	0.0	4.4	19.2	44.4	11.0	12.5	32.7	0.045 ± 0.276
Leeia	25.0	33.3	0.0	0.0	33.3	0.0	8.8	44.2	27.8	28.6	27.1	22.5	0.041 ± 0.332
Citrobacter	50.0	100.0	50.0	100.0	33.3	25.9	40.7	48.1	16.7	12.1	10.4	2.9	0.04 ± 0.451
Butyrivibrio	25.0	0.0	0.0	0.0	0.0	19.8	12.1	65.4	55.6	51.6	43.8	60.6	0.04 ± 0.113
Hydrogenophilus	0.0	0.0	0.0	0.0	0.0	0.0	2.2	0.0	0.0	15.4	0.0	2.5	0.039 ± 0.759
Proteus	30.6	0.0	0.0	0.0	0.0	0.0	16.5	3.8	0.0	4.4	16.7	1.8	0.036 ± 0.266
Lactococcus	80.6	100.0	50.0	100.0	100.0	61.7	47.3	51.9	44.4	18.7	16.7	7.8	0.035 ± 0.173
Haliangium	0.0	0.0	0.0	0.0	0.0	11.1	0.0	1.9	0.0	0.0	0.0	1.2	0.035 ± 0.874
Flavonifractor	16.7	0.0	0.0	25.0	0.0	3.7	8.8	44.2	11.1	54.9	27.1	34.5	0.034 ± 0.311
Nannocystis	0.0	0.0	0.0	0.0	0.0	1.2	0.0	0.0	0.0	0.0	0.0	1.6	0.032 ± 0.694
Enterorhabdus	38.9	0.0	0.0	0.0	0.0	6.2	14.3	30.8	16.7	42.9	33.3	59.6	0.031 ± 0.118
Cloacibacillus	0.0	0.0	0.0	0.0	0.0	1.2	0.0	40.4	0.0	14.3	2.1	23.1	0.03 ± 0.206
Anaerosporobacter	0.0	0.0	0.0	0.0	0.0	7.4	9.9	65.4	72.2	48.4	33.3	46.1	0.028 ± 0.074
Cellulosilyticum	0.0	0.0	0.0	75.0	100.0	33.3	12.1	42.3	50.0	23.1	14.6	26.1	0.028 ± 0.172
Sharpea	25.0	0.0	0.0	25.0	0.0	35.8	14.3	26.9	0.0	14.3	8.3	8.2	0.028 ± 0.253
Peptococcus	5.6	0.0	0.0	0.0	0.0	0.0	8.8	53.8	38.9	54.9	27.1	57.1	0.025 ± 0.065
Bradyrhizobium	0.0	33.3	100.0	25.0	66.7	14.8	57.1	17.3	5.6	1.1	0.0	1.6	0.024 ± 0.225
Shuttleworthia	11.1	0.0	0.0	0.0	33.3	0.0	16.5	55.8	66.7	39.6	45.8	52.5	0.024 ± 0.072
Mycoplasma	8.3	0.0	0.0	0.0	0.0	12.3	6.6	11.5	0.0	2.2	0.0	0.6	0.023 ± 0.681
Victivallis	0.0	0.0	0.0	0.0	0.0	0.0	3.3	46.2	11.1	19.8	18.8	45.3	0.022 ± 0.071
Pyramidobacter	5.6	0.0	0.0	0.0	0.0	1.2	5.5	71.2	77.8	34.1	43.8	50.8	0.021 ± 0.038
Thermobacillus	13.9	0.0	0.0	0.0	0.0	30.9	2.2	0.0	0.0	1.1	18.8	0.0	0.02 ± 0.146
Massilia	47.2	33.3	50.0	100.0	0.0	12.3	62.6	69.2	88.9	17.6	29.2	19.8	0.019 ± 0.076
Intestinimonas	0.0	0.0	0.0	0.0	0.0	2.5	1.1	67.3	5.6	42.9	0.0	33.5	0.018 ± 0.069
Bilophila	0.0	0.0	0.0	0.0	0.0	0.0	0.0	44.2	0.0	3.3	0.0	21.8	0.017 ± 0.105
Oxalobacter	2.8	0.0	0.0	0.0	0.0	1.2	5.5	48.1	11.1	41.8	39.6	48.0	0.017 ± 0.042
Paraprevotella	2.8	0.0	0.0	0.0	0.0	0.0	1.1	11.5	0.0	11.0	10.4	18.0	0.017 ± 0.204
Syntrophococcus	0.0	0.0	0.0	0.0	0.0	12.3	0.0	40.4	11.1	15.4	6.3	16.1	0.017 ± 0.147
Acidovorax	41.7	0.0	0.0	0.0	0.0	12.3	25.3	17.3	0.0	0.0	2.1	2.4	0.017 ± 0.183
Thermomonas	0.0	0.0	0.0	0.0	0.0	6.2	0.0	5.8	0.0	0.0	2.1	1.2	0.015 ± 0.35
Hydrotalea	0.0	0.0	0.0	0.0	0.0	1.2	35.2	0.0	0.0	1.1	0.0	0.6	0.015 ± 0.176
Cronobacter	2.8	33.3	50.0	25.0	0.0	21.0	1.1	46.2	0.0	2.2	0.0	2.4	0.015 ± 0.234
Epulopiscium	11.1	0.0	0.0	0.0	66.7	59.3	37.4	42.3	22.2	13.2	10.4	6.5	0.014 ± 0.122
Staphylococcus	58.3	66.7	50.0	100.0	66.7	21.0	58.2	28.8	5.6	11.0	4.2	8.8	0.014 ± 0.087
Reyranella	5.6	0.0	0.0	0.0	0.0	11.1	1.1	5.8	0.0	0.0	0.0	1.2	0.014 ± 0.209
Pasteurella	22.2	33.3	50.0	100.0	66.7	28.4	29.7	48.1	0.0	7.7	4.2	2.7	0.013 ± 0.093
Succiniclasticum	2.8	33.3	0.0	25.0	0.0	8.6	30.8	36.5	5.6	7.7	0.0	2.2	0.013 ± 0.249
Proteiniphilum	0.0	0.0	0.0	0.0	0.0	12.3	0.0	9.6	0.0	0.0	0.0	24.5	0.012 ± 0.155
Anderseniella	72.2	100.0	100.0	25.0	33.3	1.2	15.4	0.0	0.0	0.0	0.0	0.0	0.012 ± 0.157
Odoribacter	5.6	0.0	0.0	0.0	0.0	1.2	1.1	19.2	0.0	20.9	0.0	15.9	0.012 ± 0.083
Planomicrobium	19.4	33.3	0.0	75.0	0.0	40.7	24.2	28.8	0.0	9.9	6.3	23.7	0.012 ± 0.087
Allisonella	25.0	0.0	0.0	0.0	0.0	25.9	11.0	34.6	0.0	40.7	20.8	17.6	0.011 ± 0.074
Paludibacter	13.9	0.0	0.0	0.0	0.0	1.2	6.6	19.2	11.1	14.3	33.3	15.7	0.011 ± 0.054
Aeromonas	38.9	33.3	0.0	0.0	0.0	6.2	37.4	25.0	0.0	6.6	0.0	2.9	0.011 ± 0.098
Propionibacterium	47.2	33.3	100.0	50.0	33.3	18.5	64.8	17.3	5.6	2.2	6.3	0.2	0.011 ± 0.063
Sphingomonas	22.2	100.0	50.0	100.0	33.3	38.3	59.3	38.5	0.0	2.2	0.0	5.5	0.011 ± 0.052
Corynebacterium	16.7	33.3	50.0	100.0	33.3	27.2	39.6	48.1	5.6	11.0	0.0	23.7	0.01 ± 0.044
Stenotrophomonas	36.1	33.3	0.0	50.0	33.3	21.0	64.8	7.7	0.0	4.4	2.1	4.1	0.01 ± 0.065
Cloacibacterium	55.6	0.0	0.0	0.0	0.0	0.0	29.7	0.0	0.0	2.2	6.3	0.0	0.008 ± 0.074
Bacillus	36.1	33.3	0.0	75.0	0.0	60.5	26.4	13.5	0.0	2.2	6.3	16.9	0.008 ± 0.054
Moraxella	25.0	100.0	50.0	50.0	0.0	12.3	7.7	17.3	0.0	0.0	0.0	2.0	0.008 ± 0.147
Planctomyces	0.0	0.0	0.0	0.0	0.0	1.2	0.0	0.0	0.0	0.0	0.0	1.6	0.008 ± 0.149
Klebsiella	25.0	66.7	50.0	25.0	33.3	16.0	15.4	44.2	0.0	2.2	2.1	0.4	0.008 ± 0.119
Caldicoprobacter	2.8	0.0	0.0	0.0	0.0	0.0	3.3	3.8	16.7	11.0	20.8	25.7	0.008 ± 0.035
Luteimonas	11.1	0.0	0.0	0.0	0.0	9.9	16.5	7.7	38.9	0.0	18.8	2.0	0.008 ± 0.127
Gardnerella	30.6	0.0	0.0	0.0	0.0	6.2	5.5	9.6	0.0	3.3	18.8	8.2	0.008 ± 0.054
Devosia	0.0	0.0	0.0	0.0	0.0	2.5	6.6	1.9	0.0	0.0	0.0	1.8	0.008 ± 0.169
Gemmata	0.0	0.0	0.0	0.0	0.0	1.2	3.3	0.0	0.0	0.0	0.0	1.2	0.008 ± 0.155
Actinomyces	5.6	0.0	50.0	75.0	0.0	29.6	42.9	32.7	0.0	2.2	0.0	14.7	0.007 ± 0.038
Trichococcus	5.6	0.0	0.0	50.0	0.0	0.0	24.2	0.0	5.6	0.0	0.0	8.0	0.007 ± 0.059
Quinella	0.0	0.0	0.0	0.0	0.0	22.2	12.1	25.0	22.2	7.7	10.4	21.8	0.007 ± 0.023
Ignavigranum	0.0	0.0	0.0	0.0	0.0	24.7	20.9	1.9	0.0	0.0	0.0	6.1	0.007 ± 0.065

Shewanella	8.3	0.0	0.0	25.0	0.0	12.3	7.7	19.2	0.0	0.0	2.1	0.6	0.006 ± 0.063
Papillibacter	0.0	0.0	0.0	0.0	0.0	0.0	1.1	28.8	11.1	19.8	22.9	24.5	0.006 ± 0.021
Ferruginibacter	0.0	0.0	0.0	0.0	0.0	2.5	0.0	0.0	0.0	0.0	0.0	1.2	0.006 ± 0.109
Erwinia	8.3	66.7	50.0	75.0	33.3	11.1	3.3	15.4	0.0	0.0	0.0	0.0	0.006 ± 0.13
Variovorax	38.9	33.3	50.0	0.0	33.3	12.3	22.0	13.5	0.0	0.0	4.2	2.7	0.006 ± 0.061
Hydrogenoanaerobacterium	0.0	0.0	0.0	0.0	0.0	0.0	0.0	44.2	0.0	13.2	0.0	18.8	0.005 ± 0.026
Basfia	38.9	66.7	50.0	75.0	66.7	11.1	19.8	9.6	0.0	0.0	6.3	0.4	0.005 ± 0.057
Kurthia	11.1	0.0	0.0	0.0	0.0	7.4	25.3	0.0	0.0	2.2	0.0	6.5	0.005 ± 0.047
Tumebacillus	22.2	0.0	0.0	0.0	0.0	2.5	7.7	0.0	0.0	0.0	0.0	0.0	0.005 ± 0.094
Chryseobacterium	44.4	33.3	50.0	0.0	0.0	4.9	17.6	13.5	0.0	1.1	0.0	2.0	0.005 ± 0.061
Geothrix	0.0	0.0	0.0	0.0	0.0	1.2	0.0	1.9	0.0	0.0	0.0	0.4	0.005 ± 0.145
Geobacter	0.0	0.0	0.0	0.0	0.0	0.0	0.0	3.8	0.0	0.0	0.0	0.2	0.005 ± 0.15
Megamonas	0.0	0.0	0.0	0.0	0.0	1.2	0.0	21.2	5.6	2.2	0.0	12.9	0.005 ± 0.07
Comamonas	27.8	0.0	100.0	0.0	0.0	11.1	26.4	3.8	0.0	1.1	2.1	3.3	0.005 ± 0.034
Pectobacterium	36.1	0.0	0.0	0.0	0.0	3.7	19.8	17.3	0.0	0.0	2.1	0.0	0.005 ± 0.04
Serratia	2.8	0.0	0.0	0.0	0.0	3.7	9.9	0.0	0.0	0.0	0.0	8.6	0.005 ± 0.088
Butyricicoccus	0.0	0.0	0.0	0.0	0.0	0.0	3.3	23.1	27.8	16.5	10.4	12.7	0.005 ± 0.02
Raoultella	5.6	66.7	50.0	0.0	0.0	17.3	4.4	21.2	0.0	6.6	2.1	1.8	0.005 ± 0.045
Cetobacterium	0.0	0.0	0.0	25.0	0.0	22.2	3.3	15.4	0.0	0.0	0.0	1.6	0.005 ± 0.093
Parasutterella	2.8	0.0	0.0	0.0	0.0	11.1	4.4	36.5	0.0	8.8	0.0	12.4	0.004 ± 0.023
Ignatzschineria	0.0	0.0	0.0	0.0	0.0	4.9	8.8	0.0	0.0	0.0	0.0	3.7	0.004 ± 0.075
Perlucidibaca	0.0	0.0	0.0	0.0	0.0	0.0	0.0	0.0	0.0	0.0	0.0	0.4	0.004 ± 0.12
Prosthecomicrobium	0.0	0.0	0.0	0.0	0.0	0.0	0.0	0.0	0.0	0.0	0.0	0.6	0.004 ± 0.101
Polynucleobacter	0.0	0.0	0.0	0.0	0.0	4.9	0.0	0.0	0.0	0.0	0.0	1.6	0.004 ± 0.096
Phyllobacterium	5.6	0.0	0.0	25.0	66.7	19.8	19.8	32.7	0.0	5.5	2.1	4.3	0.004 ± 0.045
Sphingobium	8.3	33.3	100.0	25.0	66.7	4.9	9.9	0.0	0.0	0.0	0.0	1.2	0.004 ± 0.057
Desulfocapsa	0.0	0.0	0.0	0.0	0.0	0.0	0.0	0.0	0.0	0.0	0.0	0.4	0.004 ± 0.116
Opitutus	0.0	0.0	0.0	0.0	0.0	2.5	2.2	0.0	0.0	0.0	0.0	1.2	0.004 ± 0.077
Sulfuritalea	0.0	0.0	0.0	0.0	0.0	1.2	0.0	0.0	0.0	0.0	0.0	0.8	0.004 ± 0.067
Exiguobacterium	2.8	0.0	0.0	0.0	0.0	0.0	4.4	0.0	0.0	0.0	0.0	2.4	0.004 ± 0.103
Candidatus Cloacamonas	0.0	0.0	0.0	25.0	66.7	16.0	34.1	3.8	11.1	7.7	8.3	7.6	0.004 ± 0.016
Kluyvera	2.8	33.3	0.0	50.0	0.0	17.3	3.3	34.6	0.0	0.0	0.0	9.2	0.004 ± 0.029
vadinBC27 wastewater-sludge group	33.3	0.0	0.0	0.0	0.0	32.1	24.2	7.7	0.0	3.3	12.5	0.2	0.004 ± 0.019
Christensenella	0.0	0.0	0.0	0.0	0.0	0.0	0.0	40.4	5.6	24.2	10.4	22.4	0.003 ± 0.012
Eremococcus	19.4	0.0	0.0	0.0	0.0	0.0	28.6	0.0	5.6	0.0	0.0	4.9	0.003 ± 0.029
Mycobacterium	2.8	0.0	0.0	0.0	0.0	0.0	35.2	1.9	0.0	0.0	0.0	1.0	0.003 ± 0.031
Defluviimonas	27.8	33.3	50.0	0.0	0.0	12.3	13.2	1.9	0.0	0.0	2.1	1.2	0.003 ± 0.033
Peptostreptococcus	2.8	0.0	0.0	75.0	0.0	16.0	13.2	15.4	0.0	2.2	2.1	11.6	0.003 ± 0.034
Caryophanon	0.0	0.0	0.0	0.0	0.0	0.0	8.8	0.0	0.0	0.0	0.0	2.5	0.003 ± 0.086
Finegoldia	0.0	0.0	0.0	0.0	0.0	2.5	3.3	15.4	5.6	0.0	0.0	3.9	0.003 ± 0.049
Fastidiosipila	8.3	0.0	0.0	25.0	0.0	7.4	14.3	9.6	0.0	2.2	10.4	9.8	0.003 ± 0.021
Rothia	25.0	100.0	0.0	100.0	0.0	21.0	19.8	30.8	0.0	3.3	2.1	11.8	0.003 ± 0.012
Gemella	11.1	100.0	50.0	100.0	0.0	27.2	25.3	46.2	5.6	1.1	0.0	2.5	0.003 ± 0.023
Neisseria	44.4	100.0	50.0	50.0	0.0	7.4	17.6	23.1	0.0	1.1	0.0	0.8	0.003 ± 0.023
Porphyromonas	22.2	33.3	0.0	0.0	0.0	3.7	7.7	3.8	0.0	0.0	0.0	3.3	0.003 ± 0.033
Facklamia	0.0	0.0	0.0	0.0	0.0	39.5	20.9	5.8	0.0	0.0	0.0	4.5	0.003 ± 0.022
Sedimentibacter	0.0	0.0	0.0	0.0	33.3	25.9	38.5	19.2	5.6	11.0	6.3	7.3	0.003 ± 0.012
Mucispirillum	0.0	0.0	0.0	0.0	0.0	0.0	0.0	7.7	0.0	19.8	12.5	20.0	0.003 ± 0.013
Methylobacterium	19.4	33.3	0.0	75.0	0.0	16.0	29.7	19.2	0.0	2.2	0.0	0.6	0.003 ± 0.022
Vibrio	0.0	33.3	0.0	0.0	0.0	1.2	0.0	1.9	0.0	0.0	0.0	0.6	0.003 ± 0.085
Rickettsiella	0.0	0.0	0.0	0.0	0.0	8.6	0.0	5.8	0.0	0.0	0.0	0.4	0.003 ± 0.079
Nitratireductor	19.4	0.0	0.0	0.0	0.0	17.3	11.0	0.0	0.0	0.0	2.1	1.2	0.003 ± 0.026
Moryella	0.0	0.0	0.0	0.0	0.0	2.5	0.0	28.8	11.1	23.1	4.2	23.9	0.003 ± 0.008
Woodsholea	0.0	0.0	0.0	0.0	0.0	0.0	0.0	0.0	0.0	0.0	0.0	0.8	0.003 ± 0.052
Nitrospira	0.0	0.0	0.0	0.0	0.0	9.9	0.0	1.9	0.0	0.0	0.0	1.2	0.003 ± 0.04
Thiobacillus	0.0	0.0	0.0	0.0	0.0	3.7	0.0	0.0	0.0	0.0	0.0	0.6	0.003 ± 0.054
Azoarcus	11.1	33.3	100.0	50.0	100.0	4.9	9.9	7.7	5.6	0.0	2.1	0.2	0.002 ± 0.028
Pediococcus	5.6	33.3	0.0	25.0	0.0	24.7	0.0	3.8	0.0	3.3	0.0	1.4	0.002 ± 0.022
Aerococcus	2.8	0.0	0.0	25.0	0.0	39.5	23.1	7.7	5.6	0.0	0.0	5.3	0.002 ± 0.013
Ralstonia	16.7	0.0	0.0	75.0	33.3	3.7	36.3	0.0	0.0	3.3	0.0	0.4	0.002 ± 0.018
Anaerobiospirillum	2.8	0.0	0.0	0.0	0.0	0.0	1.1	25.0	16.7	8.8	8.3	9.8	0.002 ± 0.014
Limnohabitans	0.0	0.0	0.0	0.0	0.0	11.1	0.0	11.5	0.0	0.0	0.0	2.9	0.002 ± 0.051
Altererythrobacter	2.8	0.0	100.0	0.0	33.3	9.9	12.1	3.8	0.0	0.0	0.0	1.2	0.002 ± 0.049
Uruburuella	2.8	33.3	0.0	25.0	0.0	1.2	2.2	0.0	0.0	0.0	0.0	0.0	0.002 ± 0.058
Caldibacillus	0.0	0.0	0.0	0.0	0.0	0.0	0.0	1.9	0.0	6.6	0.0	0.8	0.002 ± 0.041
Endozoicomonas	19.4	0.0	0.0	25.0	0.0	8.6	23.1	0.0	0.0	6.6	4.2	0.0	0.002 ± 0.017
Rhizobium	19.4	66.7	0.0	50.0	0.0	13.6	26.4	1.9	0.0	1.1	0.0	1.0	0.002 ± 0.015
Allobaculum	36.1	66.7	100.0	25.0	66.7	30.9	14.3	7.7	5.6	0.0	8.3	8.6	0.002 ± 0.008
Achromobacter	22.2	0.0	0.0	0.0	0.0	4.9	23.1	23.1	0.0	1.1	0.0	2.5	0.002 ± 0.02
Synergistes	0.0	0.0	0.0	0.0	0.0	0.0	0.0	25.0	0.0	1.1	0.0	15.9	0.002 ± 0.014
Slackia	13.9	0.0	0.0	0.0	0.0	0.0	9.9	11.5	0.0	23.1	6.3	9.6	0.002 ± 0.012
Zoogloea	0.0	0.0	0.0	0.0	0.0	3.7	0.0	3.8	0.0	0.0	0.0	1.0	0.002 ± 0.034

Flavobacterium	11.1	0.0	0.0	0.0	0.0	11.1	9.9	13.5	0.0	2.2	0.0	8.2	0.002 ± 0.015
Microbacterium	11.1	33.3	0.0	100.0	0.0	18.5	12.1	3.8	0.0	0.0	0.0	0.2	0.002 ± 0.017
Lysinibacillus	0.0	0.0	0.0	0.0	0.0	1.2	5.5	0.0	0.0	0.0	0.0	6.7	0.002 ± 0.019
Jannaschia	0.0	0.0	0.0	0.0	0.0	0.0	0.0	0.0	0.0	0.0	0.0	1.0	0.002 ± 0.03
Providencia	2.8	33.3	50.0	0.0	0.0	4.9	3.3	26.9	0.0	0.0	0.0	0.8	0.002 ± 0.024
Paenibacillus	25.0	0.0	0.0	25.0	0.0	7.4	16.5	5.8	11.1	7.7	4.2	2.0	0.002 ± 0.013
Ferritrophicum	0.0	0.0	0.0	0.0	0.0	0.0	0.0	0.0	0.0	0.0	0.0	0.2	0.002 ± 0.057
Simplicispira	2.8	0.0	0.0	0.0	0.0	13.6	22.0	3.8	0.0	0.0	2.1	2.0	0.002 ± 0.014
Snodgrassella	0.0	0.0	0.0	0.0	0.0	3.7	0.0	0.0	0.0	0.0	0.0	0.0	0.002 ± 0.056
Lachnoanaerobaculum	0.0	0.0	0.0	0.0	0.0	12.3	3.3	17.3	11.1	0.0	0.0	2.2	0.002 ± 0.02
Schwartzia	0.0	0.0	0.0	0.0	0.0	0.0	0.0	0.0	0.0	8.8	0.0	6.9	0.002 ± 0.02
Aquabacterium	2.8	0.0	0.0	25.0	0.0	11.1	7.7	9.6	0.0	0.0	0.0	1.2	0.002 ± 0.018
Hirschia	0.0	0.0	0.0	0.0	0.0	0.0	3.3	0.0	0.0	0.0	0.0	1.0	0.002 ± 0.035
Phenylobacterium	0.0	0.0	0.0	0.0	0.0	1.2	3.3	7.7	0.0	1.1	0.0	1.6	0.002 ± 0.024
Bdellovibrio	0.0	0.0	0.0	0.0	0.0	0.0	0.0	1.9	0.0	0.0	0.0	1.0	0.002 ± 0.028
Smithella	0.0	0.0	0.0	0.0	0.0	12.3	0.0	7.7	0.0	0.0	0.0	13.5	0.002 ± 0.009
Howardella	2.8	0.0	0.0	0.0	0.0	24.7	6.6	26.9	0.0	11.0	2.1	10.0	0.002 ± 0.008
Pelomonas	13.9	33.3	100.0	100.0	66.7	14.8	9.9	0.0	0.0	0.0	0.0	0.0	0.002 ± 0.016
Halocella	2.8	0.0	0.0	50.0	100.0	39.5	15.4	3.8	0.0	0.0	2.1	0.6	0.002 ± 0.009
Syntrophomonas	0.0	33.3	0.0	25.0	66.7	30.9	25.3	17.3	5.6	6.6	4.2	2.5	0.002 ± 0.009
Parvimonas	0.0	0.0	0.0	75.0	0.0	11.1	7.7	1.9	0.0	0.0	0.0	1.4	0.002 ± 0.029
Olsenella	0.0	0.0	0.0	0.0	0.0	7.4	0.0	5.8	0.0	7.7	0.0	2.9	0.002 ± 0.017
Anaerovorax	0.0	0.0	0.0	0.0	0.0	7.4	0.0	23.1	0.0	6.6	4.2	16.7	0.002 ± 0.006
Sphingopyxis	16.7	0.0	0.0	25.0	33.3	12.3	14.3	3.8	0.0	0.0	0.0	1.8	0.002 ± 0.01
Anaerococcus	0.0	0.0	0.0	25.0	33.3	17.3	12.1	3.8	0.0	0.0	0.0	3.7	0.002 ± 0.012
Nordella	2.8	0.0	0.0	0.0	0.0	0.0	17.6	0.0	0.0	0.0	0.0	1.0	0.002 ± 0.014
Barnesiella	5.6	0.0	0.0	0.0	0.0	2.5	3.3	15.4	0.0	13.2	0.0	0.2	0.001 ± 0.015
Gemmobacter	19.4	0.0	0.0	25.0	0.0	3.7	12.1	3.8	0.0	0.0	2.1	0.0	0.001 ± 0.012
Solitalea	0.0	0.0	0.0	0.0	0.0	0.0	0.0	0.0	0.0	0.0	0.0	1.0	0.001 ± 0.026
Peptoniphilus	5.6	0.0	0.0	0.0	0.0	1.2	7.7	7.7	0.0	1.1	0.0	6.1	0.001 ± 0.013
Caulobacter	0.0	0.0	0.0	0.0	33.3	12.3	5.5	0.0	0.0	0.0	0.0	1.0	0.001 ± 0.026
Erysipelothrix	2.8	0.0	0.0	0.0	0.0	13.6	24.2	1.9	16.7	0.0	0.0	5.7	0.001 ± 0.007
Undibacterium	16.7	33.3	0.0	0.0	0.0	7.4	12.1	1.9	0.0	5.5	0.0	0.8	0.001 ± 0.011
Petrimonas	0.0	0.0	0.0	0.0	66.7	19.8	18.7	7.7	5.6	2.2	4.2	4.3	0.001 ± 0.007
Arcobacter	0.0	33.3	0.0	0.0	0.0	1.2	8.8	9.6	0.0	0.0	0.0	2.7	0.001 ± 0.014
Bibersteinia	8.3	33.3	50.0	50.0	0.0	8.6	7.7	25.0	5.6	0.0	0.0	0.2	0.001 ± 0.013
Acetivibrio ethanolgignens	0.0	0.0	0.0	0.0	0.0	0.0	0.0	9.6	5.6	2.2	0.0	2.4	0.001 ± 0.024
Zymomonas	0.0	0.0	0.0	0.0	0.0	0.0	0.0	0.0	0.0	0.0	0.0	0.8	0.001 ± 0.023
Vagococcus	8.3	0.0	0.0	25.0	0.0	0.0	16.5	1.9	0.0	0.0	0.0	2.7	0.001 ± 0.013
Candidatus Liberibacter	0.0	0.0	0.0	0.0	0.0	0.0	0.0	0.0	0.0	0.0	0.0	0.8	0.001 ± 0.021
Emticicia	0.0	0.0	0.0	0.0	0.0	0.0	0.0	0.0	0.0	0.0	0.0	0.8	0.001 ± 0.023
Mobiluncus	0.0	0.0	0.0	25.0	0.0	0.0	0.0	0.0	0.0	0.0	0.0	3.7	0.001 ± 0.016
Chlorochromatium	2.8	0.0	0.0	0.0	0.0	11.1	2.2	1.9	0.0	4.4	0.0	6.5	0.001 ± 0.012
Orientia	19.4	0.0	0.0	0.0	0.0	0.0	11.0	0.0	0.0	0.0	4.2	0.0	0.001 ± 0.01
Enhydrobacter	13.9	33.3	50.0	25.0	0.0	8.6	4.4	17.3	0.0	0.0	0.0	0.8	0.001 ± 0.014
Rhodopseudomonas	0.0	0.0	0.0	0.0	0.0	1.2	22.0	0.0	0.0	0.0	0.0	0.0	0.001 ± 0.017
SM1A02	0.0	0.0	0.0	0.0	0.0	0.0	0.0	0.0	0.0	0.0	0.0	0.6	0.001 ± 0.024
Rhodobacter	0.0	0.0	0.0	0.0	0.0	1.2	0.0	0.0	0.0	0.0	0.0	1.6	0.001 ± 0.023
Carnobacterium	2.8	0.0	0.0	0.0	0.0	13.6	8.8	0.0	0.0	0.0	2.1	0.8	0.001 ± 0.015
Desulfosporosinus	0.0	0.0	0.0	0.0	0.0	2.5	0.0	3.8	0.0	0.0	0.0	0.4	0.001 ± 0.033
Alicycliphilus	36.1	0.0	0.0	0.0	0.0	1.2	22.0	0.0	0.0	0.0	2.1	0.2	0.001 ± 0.007
Tatumella	8.3	0.0	0.0	25.0	0.0	8.6	12.1	23.1	0.0	0.0	0.0	0.2	0.001 ± 0.011
Delftia	13.9	0.0	0.0	0.0	0.0	4.9	18.7	3.8	0.0	0.0	0.0	1.2	0.001 ± 0.008
Jeotgalicoccus	0.0	0.0	0.0	0.0	0.0	1.2	5.5	1.9	0.0	0.0	0.0	2.9	0.001 ± 0.016
Frischella	0.0	0.0	0.0	0.0	0.0	1.2	0.0	0.0	0.0	0.0	0.0	0.0	0.001 ± 0.032
Brevundimonas	8.3	66.7	0.0	0.0	0.0	14.8	14.3	5.8	0.0	0.0	0.0	1.4	0.001 ± 0.008
Sediminibacterium	0.0	0.0	0.0	0.0	0.0	7.4	15.4	1.9	0.0	0.0	0.0	1.6	0.001 ± 0.009
Mitsuaria	0.0	0.0	0.0	0.0	0.0	0.0	0.0	0.0	0.0	0.0	0.0	0.4	0.001 ± 0.024
Proteiniclasticum	0.0	0.0	0.0	25.0	0.0	0.0	6.6	1.9	0.0	0.0	0.0	5.1	0.001 ± 0.013
Macrococcus	5.6	0.0	0.0	0.0	0.0	3.7	7.7	7.7	0.0	0.0	2.1	0.8	0.001 ± 0.016
Pseudoflavonifractor	0.0	0.0	0.0	0.0	0.0	1.2	1.1	13.5	0.0	12.1	0.0	0.6	0.001 ± 0.01
Sphingobacterium	19.4	33.3	0.0	0.0	0.0	7.4	13.2	3.8	0.0	1.1	0.0	0.8	0.001 ± 0.009
Deinococcus	2.8	0.0	0.0	0.0	0.0	1.2	3.3	0.0	0.0	0.0	0.0	0.6	0.001 ± 0.025
OM43 clade	0.0	0.0	0.0	0.0	0.0	2.5	0.0	0.0	0.0	0.0	0.0	0.8	0.001 ± 0.022
Leptothrix	0.0	0.0	0.0	0.0	0.0	9.9	0.0	0.0	0.0	0.0	0.0	1.6	0.001 ± 0.012
Morganella	5.6	0.0	0.0	0.0	33.3	12.3	8.8	26.9	0.0	0.0	0.0	1.0	0.001 ± 0.006
Curtobacterium	27.8	33.3	0.0	50.0	0.0	2.5	2.2	1.9	0.0	3.3	0.0	0.4	0.001 ± 0.008
Succinatimonas	0.0	0.0	0.0	0.0	0.0	0.0	0.0	15.4	0.0	0.0	0.0	0.2	0.001 ± 0.015
Anaerofilum	0.0	0.0	0.0	0.0	0.0	1.2	0.0	34.6	0.0	6.6	0.0	7.5	0.001 ± 0.006
Desulfobulbus	2.8	0.0	0.0	0.0	0.0	2.5	4.4	0.0	0.0	0.0	0.0	0.4	0.001 ± 0.02
Atopostipes	2.8	0.0	0.0	0.0	0.0	0.0	11.0	1.9	0.0	0.0	0.0	1.8	0.001 ± 0.01
Micrococcus	2.8	0.0	0.0	0.0	0.0	4.9	17.6	1.9	0.0	1.1	0.0	0.0	0.001 ± 0.01

Streptomyces	8.3	0.0	0.0	0.0	0.0	9.9	9.9	21.2	0.0	0.0	0.0	4.1	0.001 ± 0.007
Tissierella	0.0	0.0	0.0	0.0	0.0	2.5	6.6	0.0	0.0	0.0	0.0	4.1	0.001 ± 0.011
Bryobacter	0.0	0.0	0.0	0.0	0.0	2.5	1.1	0.0	0.0	0.0	0.0	0.8	0.001 ± 0.013
Candidatus Planktophila	0.0	0.0	0.0	0.0	0.0	0.0	0.0	0.0	0.0	0.0	0.0	0.6	0.001 ± 0.021
Anaeromyxobacter	0.0	0.0	0.0	0.0	0.0	0.0	0.0	5.8	0.0	0.0	0.0	0.4	0.001 ± 0.023
Bergeyella	8.3	0.0	0.0	0.0	0.0	0.0	5.5	3.8	0.0	0.0	0.0	0.0	0.001 ± 0.019
Actinobaculum	0.0	0.0	0.0	0.0	0.0	2.5	6.6	0.0	0.0	0.0	0.0	2.0	0.001 ± 0.015
Sideroxydans	0.0	0.0	0.0	0.0	0.0	0.0	0.0	0.0	0.0	0.0	0.0	0.4	0.001 ± 0.022
C1-B045	0.0	0.0	0.0	0.0	0.0	0.0	0.0	0.0	0.0	0.0	0.0	0.4	0.001 ± 0.02
Blastocatella	0.0	33.3	0.0	0.0	0.0	7.4	0.0	0.0	0.0	0.0	0.0	0.6	0.001 ± 0.018
Williamsia	5.6	0.0	0.0	0.0	0.0	0.0	13.2	0.0	0.0	0.0	0.0	0.0	0.001 ± 0.012
Hyphomicrobium	0.0	0.0	0.0	0.0	0.0	6.2	1.1	1.9	0.0	0.0	0.0	0.8	0.001 ± 0.014
Sporosarcina	0.0	0.0	0.0	0.0	0.0	0.0	2.2	0.0	0.0	0.0	0.0	1.4	0.001 ± 0.013
Dokdonella	11.1	0.0	0.0	0.0	0.0	1.2	13.2	0.0	0.0	0.0	0.0	1.2	0.001 ± 0.005
Helcococcus	0.0	0.0	0.0	50.0	0.0	1.2	1.1	1.9	0.0	0.0	0.0	1.8	0.001 ± 0.012
Roseiflexus	0.0	0.0	0.0	0.0	0.0	1.2	0.0	0.0	0.0	0.0	0.0	0.6	0.001 ± 0.012
Burkholderia	22.2	0.0	0.0	25.0	0.0	7.4	8.8	7.7	0.0	0.0	0.0	0.2	0.001 ± 0.006
Rhizomicrobium	2.8	0.0	0.0	0.0	0.0	0.0	1.1	1.9	0.0	0.0	0.0	1.0	0.001 ± 0.013
Thauera	2.8	0.0	0.0	0.0	0.0	12.3	3.3	3.8	0.0	0.0	0.0	0.8	0.001 ± 0.007
Sporobacter	0.0	0.0	0.0	0.0	0.0	0.0	0.0	0.0	0.0	12.1	0.0	6.7	0.001 ± 0.004
Malikia	0.0	0.0	0.0	0.0	0.0	0.0	0.0	0.0	0.0	0.0	0.0	0.8	0.001 ± 0.017
Bosea	0.0	0.0	0.0	0.0	0.0	9.9	4.4	0.0	0.0	0.0	0.0	0.6	0.001 ± 0.007
Paracoccus	2.8	0.0	0.0	0.0	0.0	6.2	12.1	5.8	0.0	1.1	0.0	0.4	0.001 ± 0.006
Flavihumibacter	0.0	0.0	0.0	0.0	0.0	0.0	1.1	0.0	0.0	0.0	0.0	0.6	0.001 ± 0.011
Pelobacter	0.0	0.0	0.0	0.0	0.0	0.0	0.0	0.0	0.0	0.0	0.0	0.2	0.001 ± 0.018
Sorangium	0.0	0.0	0.0	0.0	0.0	1.2	1.1	1.9	0.0	0.0	0.0	1.2	0.001 ± 0.008
Nitrosomonas	0.0	0.0	0.0	0.0	0.0	7.4	0.0	1.9	0.0	0.0	0.0	1.0	0.001 ± 0.011
Vogesella	0.0	0.0	0.0	0.0	0.0	1.2	0.0	0.0	0.0	0.0	0.0	1.0	0.001 ± 0.009
Gemmatimonas	0.0	0.0	0.0	0.0	0.0	1.2	0.0	0.0	0.0	0.0	0.0	0.8	0.001 ± 0.012
Pseudoramibacter	5.6	0.0	0.0	0.0	0.0	7.4	0.0	5.8	0.0	3.3	0.0	4.7	0.001 ± 0.005
Nitrobacter	0.0	0.0	0.0	0.0	0.0	7.4	19.8	0.0	0.0	0.0	0.0	0.6	0.001 ± 0.005
Paenalcaligenes	0.0	0.0	0.0	0.0	0.0	0.0	1.1	0.0	0.0	0.0	0.0	2.9	0.001 ± 0.006
Mongoliicoccus	0.0	0.0	0.0	0.0	0.0	12.3	0.0	0.0	0.0	0.0	0.0	0.0	0.001 ± 0.007
Methylobacillus	11.1	0.0	0.0	0.0	0.0	0.0	3.3	0.0	0.0	0.0	0.0	0.0	0.001 ± 0.009
Conchiformibius	5.6	33.3	50.0	25.0	33.3	2.5	3.3	7.7	0.0	0.0	0.0	0.0	0.001 ± 0.008
Edwardsiella	2.8	0.0	0.0	0.0	0.0	6.2	3.3	23.1	0.0	1.1	0.0	1.2	0.001 ± 0.008
SP3-e08	0.0	0.0	0.0	0.0	0.0	0.0	1.1	13.5	5.6	7.7	2.1	3.1	0.001 ± 0.004
Piscinibacter	0.0	0.0	0.0	0.0	0.0	9.9	0.0	3.8	0.0	0.0	0.0	1.0	0.001 ± 0.008
Noviherbaspirillum	5.6	0.0	0.0	0.0	0.0	1.2	3.3	0.0	0.0	1.1	0.0	2.9	0.001 ± 0.004
Candidatus Alysiosphaera	0.0	0.0	0.0	0.0	0.0	0.0	0.0	0.0	0.0	0.0	0.0	1.4	0.001 ± 0.009
Afipia	0.0	0.0	50.0	0.0	0.0	3.7	7.7	0.0	0.0	0.0	0.0	0.6	0 ± 0.007
Candidatus Microthrix	0.0	0.0	0.0	0.0	0.0	0.0	1.1	1.9	0.0	0.0	0.0	0.6	0 ± 0.011
Sanguibacter	0.0	0.0	0.0	0.0	0.0	1.2	4.4	0.0	0.0	0.0	0.0	0.4	0 ± 0.009
Gilliamella	0.0	0.0	0.0	0.0	0.0	2.5	0.0	0.0	0.0	0.0	0.0	0.0	0 ± 0.014
Janthinobacterium	0.0	0.0	0.0	0.0	0.0	2.5	1.1	3.8	0.0	0.0	0.0	0.2	0 ± 0.014
Mannheimia	2.8	33.3	0.0	50.0	0.0	7.4	2.2	1.9	0.0	0.0	0.0	0.0	0 ± 0.007
Marinospirillum	0.0	0.0	0.0	0.0	0.0	0.0	0.0	0.0	0.0	0.0	0.0	1.0	0 ± 0.01
Lysinimonas	2.8	0.0	0.0	0.0	0.0	1.2	1.1	0.0	0.0	0.0	0.0	0.8	0 ± 0.006
Desulfotalea	0.0	0.0	0.0	0.0	0.0	0.0	0.0	0.0	0.0	0.0	0.0	0.2	0 ± 0.013
Aquicella	0.0	0.0	0.0	0.0	0.0	0.0	0.0	0.0	0.0	0.0	0.0	0.2	0 ± 0.013
Filimonas	0.0	0.0	0.0	0.0	0.0	0.0	0.0	0.0	0.0	0.0	0.0	0.6	0 ± 0.01
Rhodovulum	0.0	0.0	0.0	0.0	0.0	6.2	2.2	0.0	0.0	0.0	0.0	1.2	0 ± 0.006
Anaerofustis	0.0	0.0	0.0	0.0	0.0	0.0	0.0	3.8	0.0	5.5	0.0	8.6	0 ± 0.002
Thioclava	0.0	0.0	0.0	0.0	0.0	0.0	0.0	1.9	0.0	0.0	0.0	1.0	0 ± 0.007
Pirellula	0.0	0.0	0.0	0.0	0.0	2.5	0.0	0.0	0.0	0.0	0.0	0.8	0 ± 0.007
Candidatus Profftella	2.8	0.0	0.0	25.0	0.0	1.2	17.6	0.0	0.0	0.0	0.0	0.2	0 ± 0.004
Patulibacter	0.0	0.0	0.0	0.0	0.0	1.2	1.1	0.0	0.0	0.0	0.0	0.4	0 ± 0.01
Legionella	0.0	0.0	0.0	0.0	0.0	0.0	0.0	0.0	0.0	0.0	0.0	1.0	0 ± 0.006
Globicatella	2.8	0.0	0.0	50.0	0.0	3.7	4.4	5.8	0.0	0.0	0.0	2.2	0 ± 0.004
Methyloversatilis	0.0	0.0	0.0	0.0	0.0	11.1	0.0	1.9	0.0	0.0	0.0	0.4	0 ± 0.007
Gallicola	0.0	0.0	0.0	0.0	0.0	9.9	6.6	0.0	0.0	0.0	0.0	1.8	0 ± 0.004
Aquamicrobium	0.0	33.3	0.0	0.0	0.0	1.2	6.6	1.9	0.0	2.2	2.1	0.4	0 ± 0.006
Trueperella	0.0	0.0	0.0	0.0	0.0	1.2	3.3	5.8	0.0	0.0	0.0	3.9	0 ± 0.005
Candidatus Hepatincola	0.0	0.0	0.0	0.0	0.0	0.0	0.0	3.8	0.0	0.0	0.0	2.5	0 ± 0.004
Herbaspirillum	0.0	0.0	0.0	0.0	0.0	4.9	0.0	0.0	0.0	13.2	0.0	2.5	0 ± 0.004
Coprothermobacter	0.0	0.0	0.0	0.0	0.0	0.0	13.2	0.0	5.6	0.0	0.0	0.2	0 ± 0.004
Desulfatiferula	0.0	0.0	0.0	0.0	0.0	0.0	0.0	0.0	0.0	0.0	0.0	0.4	0 ± 0.01
Mesorhizobium	8.3	0.0	0.0	50.0	0.0	7.4	12.1	5.8	0.0	0.0	0.0	0.0	0 ± 0.003
Pseudoclavibacter	13.9	0.0	0.0	0.0	0.0	1.2	2.2	0.0	0.0	1.1	0.0	0.4	0 ± 0.004
Polaromonas	0.0	0.0	0.0	0.0	0.0	9.9	1.1	0.0	0.0	0.0	0.0	1.0	0 ± 0.004
Leucobacter	5.6	0.0	0.0	0.0	0.0	6.2	3.3	3.8	0.0	1.1	0.0	2.0	0 ± 0.004
Kocuria	5.6	0.0	0.0	75.0	33.3	1.2	8.8	17.3	0.0	0.0	0.0	0.4	0 ± 0.004

Parvibaculum	0.0	33.3	50.0	0.0	66.7	0.0	0.0	0.0	0.0	0.0	0.0	0.2	0 ± 0.006
Ochrobactrum	0.0	33.3	0.0	25.0	0.0	7.4	7.7	1.9	0.0	1.1	0.0	0.6	0 ± 0.003
Roseomonas	0.0	0.0	0.0	0.0	0.0	1.2	2.2	0.0	0.0	0.0	0.0	1.0	0 ± 0.005
Brevibacterium	5.6	0.0	0.0	0.0	0.0	11.1	4.4	5.8	0.0	1.1	0.0	1.0	0 ± 0.004
Macellibacteroides	0.0	0.0	0.0	0.0	0.0	0.0	0.0	3.8	0.0	0.0	0.0	0.2	0 ± 0.009
Brucella	0.0	0.0	0.0	25.0	0.0	0.0	8.8	0.0	0.0	0.0	0.0	0.0	0 ± 0.004
Methylotenera	0.0	0.0	0.0	0.0	0.0	0.0	0.0	0.0	0.0	0.0	0.0	0.6	0 ± 0.007
Saccharofermentans	8.3	0.0	0.0	0.0	0.0	0.0	0.0	7.7	0.0	0.0	8.3	1.4	0 ± 0.003
Rubrobacter	0.0	0.0	0.0	0.0	0.0	11.1	0.0	3.8	0.0	0.0	0.0	0.0	0 ± 0.004
Nevskia	0.0	0.0	0.0	0.0	0.0	7.4	0.0	0.0	0.0	0.0	0.0	0.4	0 ± 0.006
Terrimonas	0.0	0.0	0.0	0.0	0.0	0.0	0.0	0.0	0.0	0.0	0.0	0.6	0 ± 0.008
Otariodibacter	0.0	33.3	0.0	50.0	0.0	3.7	5.5	5.8	0.0	1.1	0.0	1.2	0 ± 0.003
Ornatilinea	2.8	0.0	0.0	0.0	0.0	2.5	2.2	0.0	0.0	0.0	0.0	0.2	0 ± 0.007
Sulfuricurvum	0.0	0.0	0.0	0.0	0.0	0.0	0.0	0.0	0.0	0.0	0.0	0.4	0 ± 0.008
Bartonella	0.0	0.0	0.0	0.0	0.0	1.2	0.0	0.0	0.0	0.0	0.0	0.0	0 ± 0.009
Pedobacter	11.1	0.0	0.0	0.0	0.0	4.9	1.1	1.9	0.0	1.1	0.0	0.4	0 ± 0.004
Luteococcus	2.8	33.3	0.0	75.0	33.3	0.0	6.6	1.9	0.0	0.0	0.0	0.0	0 ± 0.003
Rickettsia	0.0	0.0	0.0	0.0	0.0	1.2	0.0	1.9	0.0	0.0	0.0	0.6	0 ± 0.005
Synechococcus	0.0	0.0	0.0	0.0	0.0	1.2	0.0	0.0	0.0	0.0	0.0	2.5	0 ± 0.003
Alpinimonas	0.0	0.0	0.0	0.0	0.0	0.0	0.0	5.8	0.0	0.0	0.0	0.6	0 ± 0.007
Gallibacterium	0.0	0.0	0.0	0.0	0.0	11.1	0.0	15.4	0.0	0.0	0.0	0.2	0 ± 0.003
Acetatifactor	0.0	0.0	0.0	0.0	0.0	0.0	0.0	7.7	22.2	0.0	2.1	0.6	0 ± 0.003
Pedomicrobium	0.0	0.0	0.0	0.0	0.0	2.5	1.1	0.0	0.0	0.0	0.0	1.0	0 ± 0.005
Hymenobacter	2.8	0.0	0.0	0.0	0.0	2.5	1.1	0.0	0.0	0.0	0.0	0.2	0 ± 0.005
Aggregatibacter	0.0	0.0	0.0	0.0	0.0	6.2	1.1	0.0	0.0	1.1	0.0	2.9	0 ± 0.002
Azospira	0.0	0.0	0.0	0.0	0.0	6.2	0.0	1.9	0.0	0.0	2.1	0.8	0 ± 0.003
Eubacterium	0.0	0.0	0.0	0.0	0.0	0.0	0.0	17.3	0.0	2.2	0.0	2.4	0 ± 0.004
Ottowia	0.0	0.0	0.0	0.0	0.0	11.1	0.0	5.8	0.0	0.0	0.0	0.8	0 ± 0.003
Cedecea	0.0	0.0	0.0	0.0	0.0	1.2	2.2	26.9	0.0	1.1	0.0	0.0	0 ± 0.003
Methylophilus	0.0	0.0	0.0	0.0	0.0	2.5	0.0	0.0	0.0	0.0	0.0	0.6	0 ± 0.005
Cellvibrio	0.0	0.0	0.0	0.0	0.0	0.0	3.3	0.0	0.0	0.0	0.0	0.6	0 ± 0.006
Brachymonas	0.0	0.0	0.0	25.0	0.0	7.4	0.0	3.8	0.0	0.0	0.0	1.4	0 ± 0.003
Brachybacterium	8.3	0.0	0.0	0.0	33.3	3.7	5.5	1.9	0.0	2.2	0.0	0.2	0 ± 0.003
Thermincola	0.0	0.0	0.0	0.0	0.0	0.0	0.0	0.0	0.0	0.0	0.0	0.2	0 ± 0.007
Clostridiales bacterium 20-2a	0.0	0.0	0.0	0.0	0.0	0.0	0.0	7.7	0.0	0.0	0.0	2.2	0 ± 0.003
Murdochiella	0.0	0.0	0.0	25.0	0.0	0.0	0.0	1.9	0.0	0.0	0.0	3.3	0 ± 0.002
Frigoribacterium	5.6	0.0	0.0	25.0	0.0	0.0	1.1	0.0	0.0	0.0	0.0	0.0	0 ± 0.005
Stella	0.0	0.0	0.0	0.0	0.0	0.0	0.0	0.0	0.0	0.0	0.0	0.4	0 ± 0.005
Fonticella	5.6	0.0	0.0	0.0	0.0	0.0	7.7	5.8	5.6	0.0	0.0	0.4	0 ± 0.002
Candidatus Captivus	0.0	0.0	0.0	0.0	0.0	0.0	0.0	0.0	0.0	0.0	2.1	0.8	0 ± 0.004
Diaphorobacter	8.3	0.0	0.0	0.0	0.0	0.0	6.6	0.0	0.0	0.0	0.0	0.0	0 ± 0.002
Wautersiella	0.0	33.3	0.0	0.0	0.0	2.5	1.1	0.0	0.0	0.0	0.0	1.8	0 ± 0.002
Solibacillus	0.0	0.0	0.0	0.0	0.0	1.2	2.2	0.0	0.0	0.0	0.0	2.4	0 ± 0.003
Anoxybacillus	0.0	0.0	0.0	0.0	0.0	1.2	3.3	5.8	0.0	0.0	0.0	0.0	0 ± 0.004
Luteolibacter	0.0	0.0	0.0	0.0	0.0	0.0	0.0	0.0	0.0	0.0	0.0	0.8	0 ± 0.004
Dietzia	0.0	0.0	0.0	0.0	0.0	1.2	6.6	3.8	0.0	1.1	0.0	0.6	0 ± 0.003
Pelagibacterium	0.0	0.0	0.0	0.0	0.0	0.0	0.0	0.0	0.0	2.2	0.0	0.2	0 ± 0.005
GKS98 freshwater group	0.0	0.0	0.0	0.0	0.0	0.0	0.0	0.0	0.0	0.0	0.0	0.4	0 ± 0.005
Eikenella	5.6	0.0	0.0	0.0	0.0	0.0	0.0	0.0	0.0	0.0	0.0	0.0	0 ± 0.006
Fructobacillus	0.0	0.0	0.0	0.0	0.0	18.5	0.0	0.0	0.0	0.0	0.0	0.0	0 ± 0.002
Pseudospirillum	2.8	0.0	0.0	0.0	0.0	1.2	5.5	0.0	0.0	0.0	0.0	0.2	0 ± 0.003
Cohnella	0.0	0.0	0.0	0.0	0.0	0.0	1.1	0.0	0.0	0.0	0.0	0.0	0 ± 0.006
Robinsoniella	0.0	0.0	0.0	0.0	0.0	0.0	0.0	7.7	11.1	1.1	2.1	0.4	0 ± 0.003
Rhodobium	0.0	0.0	0.0	0.0	0.0	1.2	0.0	1.9	0.0	0.0	0.0	0.8	0 ± 0.003
Arenimonas	0.0	0.0	0.0	0.0	0.0	1.2	0.0	1.9	0.0	0.0	0.0	0.8	0 ± 0.003
Runella	0.0	0.0	0.0	0.0	0.0	0.0	0.0	0.0	0.0	0.0	0.0	0.6	0 ± 0.004
Erythrobacter	0.0	0.0	0.0	0.0	0.0	3.7	0.0	0.0	0.0	0.0	0.0	0.2	0 ± 0.005
Defluviicoccus	0.0	0.0	0.0	0.0	0.0	2.5	0.0	0.0	0.0	0.0	0.0	0.4	0 ± 0.004
Prochlorococcus	0.0	0.0	0.0	0.0	0.0	1.2	0.0	5.8	0.0	0.0	0.0	1.4	0 ± 0.003
Abiotrophia	0.0	0.0	0.0	0.0	0.0	0.0	0.0	1.9	0.0	8.8	0.0	0.0	0 ± 0.002
Methylobacter	0.0	0.0	0.0	0.0	0.0	0.0	0.0	0.0	0.0	0.0	0.0	0.2	0 ± 0.005
Acidiferrobacter	0.0	0.0	0.0	0.0	0.0	1.2	0.0	0.0	0.0	0.0	0.0	0.6	0 ± 0.003
Blastomonas	2.8	0.0	0.0	0.0	33.3	9.9	4.4	0.0	0.0	0.0	2.1	0.0	0 ± 0.002
Tolumonas	0.0	0.0	0.0	0.0	0.0	3.7	0.0	0.0	0.0	0.0	0.0	0.2	0 ± 0.005
Yersinia	0.0	0.0	0.0	0.0	0.0	2.5	6.6	0.0	0.0	0.0	0.0	0.0	0 ± 0.002
Nocardioides	0.0	0.0	0.0	0.0	0.0	2.5	6.6	5.8	0.0	0.0	0.0	0.8	0 ± 0.002
Cytophaga	0.0	0.0	0.0	0.0	0.0	0.0	0.0	0.0	0.0	0.0	0.0	0.6	0 ± 0.004
Hespellia	0.0	0.0	0.0	0.0	0.0	0.0	0.0	7.7	11.1	0.0	2.1	1.6	0 ± 0.002
Eggerthella	0.0	0.0	0.0	0.0	0.0	0.0	0.0	0.0	0.0	1.1	0.0	2.5	0 ± 0.001
Byssovorax	0.0	0.0	0.0	0.0	0.0	0.0	0.0	0.0	0.0	0.0	0.0	0.2	0 ± 0.005
Cupriavidus	2.8	0.0	0.0	0.0	0.0	7.4	2.2	1.9	0.0	0.0	0.0	0.4	0 ± 0.002
Virgibacillus	0.0	0.0	0.0	0.0	0.0	1.2	0.0	0.0	0.0	0.0	0.0	1.8	0 ± 0.004

Tardiphaga	0.0	0.0	0.0	0.0	0.0	0.0	5.5	0.0	0.0	0.0	0.0	0.4	0 ± 0.002
Salmonella	2.8	0.0	0.0	0.0	0.0	1.2	0.0	0.0	0.0	1.1	0.0	0.6	0 ± 0.003
Chthoniobacter	0.0	0.0	0.0	0.0	0.0	1.2	0.0	0.0	0.0	0.0	0.0	0.6	0 ± 0.004
Desulfatirhabdium	0.0	0.0	0.0	0.0	0.0	0.0	0.0	0.0	0.0	0.0	0.0	0.2	0 ± 0.005
Hafnia	5.6	0.0	0.0	0.0	0.0	6.2	4.4	0.0	0.0	0.0	0.0	0.0	0 ± 0.002
Lacticigenium	0.0	0.0	0.0	0.0	0.0	0.0	2.2	0.0	0.0	0.0	0.0	1.0	0 ± 0.002
Arcanobacterium	0.0	0.0	0.0	0.0	0.0	0.0	0.0	0.0	0.0	0.0	0.0	2.4	0 ± 0.002
Acidocella	0.0	0.0	0.0	0.0	0.0	1.2	0.0	0.0	0.0	0.0	0.0	0.4	0 ± 0.003
Candidatus Competibacter	0.0	0.0	0.0	0.0	0.0	2.5	0.0	0.0	0.0	0.0	0.0	1.4	0 ± 0.003
Azospirillum	0.0	0.0	0.0	0.0	0.0	1.2	1.1	0.0	0.0	0.0	0.0	0.2	0 ± 0.004
Roseateles	2.8	0.0	50.0	0.0	0.0	7.4	0.0	0.0	0.0	0.0	0.0	0.2	0 ± 0.002
Rathayibacter	8.3	0.0	0.0	0.0	0.0	1.2	0.0	0.0	0.0	0.0	0.0	0.2	0 ± 0.002
Rudaea	0.0	0.0	0.0	0.0	0.0	1.2	7.7	0.0	0.0	0.0	0.0	0.0	0 ± 0.002
Kineosporia	0.0	0.0	0.0	0.0	0.0	0.0	0.0	1.9	0.0	0.0	0.0	0.6	0 ± 0.003
Longilinea	0.0	0.0	0.0	0.0	0.0	0.0	0.0	0.0	0.0	0.0	0.0	0.2	0 ± 0.004
LD28 freshwater group	0.0	0.0	0.0	0.0	0.0	0.0	0.0	0.0	0.0	0.0	0.0	0.8	0 ± 0.003
Kitasatospora	0.0	0.0	0.0	0.0	0.0	3.7	2.2	0.0	0.0	0.0	2.1	0.2	0 ± 0.002
CL500-3	0.0	0.0	0.0	0.0	0.0	0.0	0.0	0.0	0.0	0.0	0.0	1.2	0 ± 0.002
Marinobacter	0.0	0.0	0.0	0.0	0.0	2.5	4.4	0.0	0.0	0.0	0.0	0.2	0 ± 0.002
Isoptericola	0.0	0.0	0.0	0.0	0.0	0.0	0.0	0.0	0.0	1.1	0.0	1.2	0 ± 0.003
Desulfopila	0.0	0.0	0.0	0.0	0.0	0.0	0.0	0.0	0.0	0.0	0.0	0.2	0 ± 0.004
Sandarakinorhabdus	0.0	0.0	0.0	0.0	0.0	0.0	0.0	0.0	0.0	0.0	0.0	0.8	0 ± 0.002
Denitratisoma	0.0	0.0	0.0	0.0	0.0	1.2	0.0	0.0	0.0	0.0	0.0	0.4	0 ± 0.003
Desulfuromonas	0.0	0.0	0.0	0.0	0.0	3.7	0.0	0.0	0.0	0.0	0.0	0.2	0 ± 0.004
Myroides	2.8	0.0	0.0	0.0	0.0	0.0	1.1	0.0	0.0	0.0	0.0	0.2	0 ± 0.003
Gelria	0.0	0.0	0.0	0.0	0.0	0.0	0.0	0.0	0.0	0.0	0.0	2.5	0 ± 0.001
Catellicoccus	0.0	0.0	0.0	0.0	0.0	0.0	0.0	0.0	0.0	0.0	0.0	1.8	0 ± 0.002
Spirosoma	0.0	0.0	0.0	0.0	0.0	0.0	2.2	0.0	0.0	0.0	0.0	0.0	0 ± 0.003
Gordonia	2.8	0.0	0.0	0.0	0.0	6.2	2.2	7.7	0.0	0.0	0.0	0.4	0 ± 0.002
Roseovarius	0.0	0.0	0.0	0.0	0.0	2.5	2.2	0.0	0.0	0.0	0.0	0.2	0 ± 0.002
Pseudarcicella	0.0	0.0	0.0	0.0	0.0	0.0	0.0	0.0	0.0	0.0	0.0	0.6	0 ± 0.003
Albidiferax	0.0	0.0	0.0	0.0	0.0	3.7	0.0	0.0	0.0	0.0	0.0	0.6	0 ± 0.002
Desulfotomaculum	0.0	0.0	0.0	0.0	0.0	0.0	0.0	3.8	0.0	0.0	0.0	0.2	0 ± 0.004
Deferrisoma	0.0	0.0	0.0	0.0	0.0	0.0	0.0	0.0	0.0	0.0	0.0	0.2	0 ± 0.004
Limnobacter	2.8	0.0	50.0	25.0	33.3	0.0	1.1	0.0	0.0	0.0	0.0	0.0	0 ± 0.002
Inquilinus	0.0	0.0	0.0	0.0	0.0	0.0	0.0	0.0	0.0	0.0	0.0	0.6	0 ± 0.002
Listeria	0.0	0.0	0.0	0.0	0.0	0.0	4.4	0.0	0.0	0.0	2.1	0.8	0 ± 0.001
Iamia	0.0	0.0	0.0	0.0	0.0	0.0	1.1	0.0	0.0	0.0	0.0	0.2	0 ± 0.004
Wolbachia	2.8	0.0	0.0	0.0	0.0	0.0	4.4	0.0	0.0	0.0	0.0	0.0	0 ± 0.002
Cellulomonas	0.0	0.0	0.0	0.0	0.0	0.0	1.1	1.9	0.0	0.0	0.0	0.8	0 ± 0.002
Curvibacter	0.0	0.0	0.0	0.0	0.0	8.6	0.0	1.9	0.0	0.0	0.0	0.6	0 ± 0.002
12up	0.0	0.0	0.0	0.0	0.0	0.0	0.0	0.0	0.0	0.0	0.0	0.4	0 ± 0.003
Isosphaera	0.0	0.0	0.0	0.0	0.0	1.2	0.0	0.0	0.0	0.0	0.0	0.8	0 ± 0.002
Paraeggerthella	0.0	0.0	0.0	0.0	0.0	1.2	0.0	3.8	0.0	0.0	0.0	0.0	0 ± 0.003
Epilithonimonas	5.6	0.0	0.0	0.0	0.0	0.0	1.1	0.0	0.0	0.0	0.0	0.0	0 ± 0.002
Nocardia	0.0	0.0	0.0	0.0	0.0	1.2	0.0	0.0	0.0	0.0	0.0	0.8	0 ± 0.002
Fusibacter	0.0	0.0	0.0	0.0	33.3	2.5	0.0	5.8	0.0	0.0	0.0	0.4	0 ± 0.001
Anaerobacillus	0.0	0.0	0.0	0.0	0.0	0.0	0.0	0.0	0.0	0.0	0.0	2.0	0 ± 0.001
Paucimonas	0.0	0.0	0.0	0.0	0.0	0.0	0.0	1.9	0.0	0.0	0.0	0.4	0 ± 0.002
Roseobacter clade CHAB-I-5 lineage	0.0	0.0	0.0	0.0	0.0	2.5	0.0	0.0	0.0	0.0	0.0	0.4	0 ± 0.002
Syntrophobacter	0.0	0.0	0.0	0.0	0.0	0.0	1.1	0.0	0.0	0.0	0.0	0.2	0 ± 0.002
Leptotrichia	2.8	0.0	0.0	25.0	0.0	1.2	2.2	13.5	0.0	0.0	0.0	0.4	0 ± 0.001
Candidatus Solibacter	0.0	0.0	0.0	0.0	0.0	3.7	0.0	0.0	0.0	0.0	0.0	0.4	0 ± 0.002
Acholeplasma	0.0	0.0	0.0	0.0	0.0	0.0	0.0	0.0	0.0	0.0	0.0	0.6	0 ± 0.002
Hydrogenophaga	0.0	0.0	0.0	0.0	0.0	7.4	0.0	5.8	0.0	0.0	0.0	0.6	0 ± 0.001
Candidatus Odyssella	0.0	0.0	0.0	0.0	0.0	0.0	0.0	0.0	0.0	0.0	0.0	0.4	0 ± 0.002
Aequorivita	0.0	0.0	0.0	0.0	0.0	0.0	0.0	0.0	0.0	0.0	0.0	0.4	0 ± 0.002
Beggiatoa	2.8	0.0	0.0	0.0	0.0	0.0	2.2	0.0	0.0	0.0	0.0	0.0	0 ± 0.002
Dechloromonas	0.0	0.0	0.0	0.0	0.0	3.7	0.0	0.0	0.0	0.0	0.0	0.2	0 ± 0.002
Coxiella	0.0	0.0	0.0	0.0	33.3	0.0	0.0	0.0	0.0	0.0	0.0	0.4	0 ± 0.002
Janibacter	0.0	0.0	0.0	0.0	0.0	8.6	1.1	1.9	0.0	1.1	0.0	0.2	0 ± 0.001
Pseudorhodoferax	0.0	0.0	0.0	0.0	0.0	2.5	0.0	0.0	0.0	0.0	0.0	0.4	0 ± 0.002
Pseudogulbenkiania	0.0	0.0	0.0	0.0	0.0	0.0	0.0	0.0	0.0	0.0	0.0	0.2	0 ± 0.003
Geobacillus	2.8	0.0	0.0	0.0	0.0	0.0	2.2	1.9	0.0	3.3	0.0	0.8	0 ± 0.001
Okibacterium	8.3	0.0	0.0	0.0	0.0	0.0	1.1	0.0	0.0	0.0	0.0	0.0	0 ± 0.002
Candidatus Metachlamydia	0.0	0.0	0.0	0.0	0.0	0.0	0.0	0.0	0.0	0.0	0.0	0.2	0 ± 0.003
Thermosinus	0.0	0.0	0.0	0.0	0.0	0.0	0.0	0.0	0.0	1.1	0.0	0.2	0 ± 0.002
Porphyrobacter	2.8	0.0	0.0	0.0	0.0	4.9	1.1	0.0	0.0	0.0	0.0	0.0	0 ± 0.002
Tahibacter	0.0	0.0	0.0	0.0	0.0	0.0	0.0	1.9	0.0	0.0	0.0	0.4	0 ± 0.002
Gaiella	0.0	0.0	0.0	0.0	0.0	2.5	2.2	0.0	0.0	0.0	0.0	0.2	0 ± 0.002
Olivibacter	0.0	0.0	0.0	0.0	0.0	1.2	1.1	0.0	0.0	0.0	0.0	0.2	0 ± 0.003
Pseudolabrys	0.0	0.0	0.0	0.0	0.0	0.0	1.1	1.9	0.0	0.0	0.0	0.2	0 ± 0.002

Dongia	2.8	0.0	0.0	0.0	0.0	2.5	1.1	1.9	0.0	0.0	0.0	0.2	0 ± 0.002
Zhihengliuella	0.0	0.0	0.0	0.0	0.0	0.0	0.0	0.0	0.0	0.0	0.0	0.4	0 ± 0.002
Prosthecobacter	0.0	0.0	0.0	0.0	0.0	1.2	0.0	0.0	0.0	0.0	0.0	0.4	0 ± 0.002
Microcystis	2.8	0.0	0.0	0.0	0.0	0.0	2.2	0.0	0.0	0.0	0.0	0.6	0 ± 0.001
Sulfuricella	0.0	0.0	0.0	0.0	0.0	0.0	0.0	0.0	0.0	0.0	0.0	0.2	0 ± 0.003
Bacteriovorax	0.0	0.0	0.0	0.0	0.0	0.0	0.0	0.0	0.0	0.0	0.0	0.2	0 ± 0.003
Marmoricola	0.0	0.0	0.0	0.0	0.0	0.0	2.2	1.9	0.0	0.0	0.0	0.4	0 ± 0.002
Wohlfahrtiimonas	0.0	0.0	0.0	0.0	0.0	0.0	0.0	0.0	0.0	0.0	0.0	2.4	0 ± 0.001
Tetrasphaera	0.0	0.0	0.0	0.0	0.0	1.2	5.5	11.5	0.0	0.0	0.0	0.0	0 ± 0.001
Blastococcus	2.8	0.0	0.0	0.0	33.3	0.0	1.1	0.0	0.0	0.0	0.0	0.2	0 ± 0.001
Cryobacterium	0.0	0.0	0.0	0.0	0.0	0.0	0.0	0.0	0.0	0.0	0.0	0.6	0 ± 0.002
Azohydromonas	0.0	0.0	0.0	0.0	0.0	0.0	0.0	0.0	0.0	0.0	0.0	0.2	0 ± 0.002
Ornithinibacter	0.0	0.0	0.0	0.0	0.0	0.0	0.0	1.9	0.0	0.0	0.0	0.4	0 ± 0.002
Agromyces	0.0	0.0	0.0	0.0	0.0	4.9	1.1	0.0	0.0	0.0	0.0	0.4	0 ± 0.001
Pseudochrobactrum	2.8	0.0	0.0	0.0	0.0	0.0	0.0	0.0	0.0	0.0	0.0	0.2	0 ± 0.002
Alloiococcus	0.0	0.0	0.0	0.0	0.0	1.2	0.0	0.0	0.0	0.0	0.0	1.4	0 ± 0.001
Rahnella	0.0	0.0	0.0	0.0	0.0	1.2	0.0	5.8	0.0	1.1	0.0	0.2	0 ± 0.002
Alkalibacter	0.0	0.0	0.0	0.0	0.0	0.0	0.0	0.0	0.0	0.0	0.0	1.0	0 ± 0.001
Microvirgula	0.0	0.0	0.0	0.0	0.0	8.6	0.0	7.7	0.0	0.0	0.0	0.0	0 ± 0.001
Alkanindiges	0.0	0.0	0.0	0.0	0.0	0.0	0.0	0.0	0.0	0.0	0.0	0.4	0 ± 0.002
Clavibacter	2.8	0.0	0.0	50.0	0.0	0.0	0.0	1.9	0.0	0.0	0.0	0.2	0 ± 0.001
Renibacterium	0.0	0.0	0.0	0.0	0.0	9.9	0.0	1.9	0.0	0.0	0.0	0.0	0 ± 0.001
Acetanaerobacterium	0.0	0.0	0.0	0.0	0.0	0.0	0.0	17.3	5.6	1.1	0.0	0.4	0 ± 0.001
Oligella	0.0	0.0	0.0	0.0	0.0	0.0	0.0	0.0	0.0	0.0	0.0	1.0	0 ± 0.002
Flavisolibacter	2.8	0.0	0.0	0.0	0.0	3.7	0.0	0.0	0.0	0.0	0.0	0.2	0 ± 0.001
Pseudoalteromonas	0.0	0.0	0.0	0.0	0.0	8.6	1.1	3.8	0.0	0.0	0.0	0.0	0 ± 0.001
Johnsonella	0.0	0.0	0.0	0.0	0.0	1.2	0.0	5.8	0.0	0.0	0.0	0.8	0 ± 0.001
Tepidimonas	2.8	0.0	0.0	0.0	0.0	0.0	2.2	0.0	0.0	0.0	0.0	0.2	0 ± 0.001
Dyadobacter	5.6	0.0	0.0	0.0	0.0	0.0	0.0	0.0	0.0	0.0	0.0	0.2	0 ± 0.001
Azovibrio	0.0	0.0	0.0	0.0	0.0	0.0	0.0	0.0	0.0	0.0	0.0	0.2	0 ± 0.002
Shimwellia	0.0	0.0	0.0	0.0	0.0	9.9	0.0	0.0	0.0	0.0	0.0	0.2	0 ± 0.001
Tepidiphilus	0.0	0.0	0.0	0.0	0.0	0.0	0.0	0.0	0.0	0.0	0.0	0.4	0 ± 0.002
Desemzia	0.0	0.0	0.0	0.0	0.0	0.0	5.5	0.0	0.0	0.0	0.0	0.0	0 ± 0.001
Pusillimonas	0.0	0.0	0.0	0.0	0.0	0.0	0.0	0.0	0.0	0.0	0.0	0.8	0 ± 0.001
Sphingosinicella	0.0	0.0	0.0	0.0	0.0	7.4	1.1	3.8	0.0	0.0	0.0	0.2	0 ± 0.001
Leptolinea	0.0	0.0	0.0	0.0	0.0	0.0	0.0	0.0	0.0	0.0	0.0	0.2	0 ± 0.002
Desulfurivibrio	0.0	0.0	0.0	0.0	0.0	0.0	0.0	0.0	0.0	0.0	0.0	0.2	0 ± 0.002
Ureibacillus	0.0	0.0	0.0	0.0	0.0	0.0	3.3	0.0	0.0	0.0	0.0	0.0	0 ± 0.001
Chitinibacter	0.0	0.0	0.0	0.0	0.0	0.0	0.0	0.0	0.0	0.0	0.0	0.2	0 ± 0.002
Thermus	0.0	0.0	0.0	0.0	0.0	1.2	0.0	0.0	0.0	0.0	0.0	0.6	0 ± 0.001
Candidatus Methylacidiphilum	0.0	0.0	0.0	0.0	0.0	0.0	0.0	0.0	0.0	0.0	0.0	0.4	0 ± 0.001
Tessaracoccus	0.0	0.0	0.0	0.0	0.0	2.5	3.3	7.7	0.0	0.0	0.0	0.0	0 ± 0.001
Fluviicola	0.0	0.0	0.0	0.0	0.0	1.2	0.0	3.8	0.0	0.0	0.0	0.8	0 ± 0.001
Rhodococcus sp. BBCT 63	0.0	0.0	0.0	0.0	0.0	0.0	1.1	0.0	0.0	0.0	0.0	0.0	0 ± 0.002
Salinicoccus	0.0	0.0	0.0	0.0	0.0	0.0	0.0	0.0	0.0	1.1	0.0	0.0	0 ± 0.002
Saccharopolyspora	0.0	0.0	0.0	0.0	0.0	9.9	1.1	0.0	0.0	0.0	0.0	0.0	0 ± 0.001
Ensifer	0.0	0.0	0.0	0.0	0.0	0.0	1.1	0.0	0.0	0.0	0.0	0.4	0 ± 0.001
Wolinella	0.0	0.0	0.0	0.0	0.0	0.0	0.0	11.5	0.0	0.0	0.0	0.0	0 ± 0.001
Nitrosococcus	2.8	0.0	0.0	0.0	0.0	0.0	0.0	0.0	0.0	0.0	0.0	0.4	0 ± 0.001
Nosocomiicoccus	0.0	0.0	0.0	0.0	0.0	4.9	0.0	0.0	0.0	1.1	0.0	0.8	0 ± 0.001
Georgenia	0.0	0.0	0.0	0.0	0.0	0.0	1.1	0.0	0.0	0.0	0.0	0.0	0 ± 0.002
Armatimonas	0.0	0.0	0.0	0.0	0.0	0.0	0.0	0.0	0.0	0.0	0.0	0.4	0 ± 0.001
Plesiomonas	0.0	0.0	0.0	0.0	0.0	4.9	0.0	0.0	0.0	0.0	0.0	0.6	0 ± 0.001
Acetobacterium	0.0	0.0	0.0	0.0	0.0	0.0	0.0	0.0	0.0	0.0	0.0	0.2	0 ± 0.002
Candidatus Nitrotoga	0.0	0.0	0.0	0.0	0.0	0.0	0.0	0.0	0.0	0.0	0.0	0.2	0 ± 0.002
Desulfarculus	0.0	0.0	0.0	0.0	0.0	0.0	0.0	0.0	0.0	0.0	0.0	0.2	0 ± 0.002
Tabrizicola	0.0	0.0	0.0	0.0	0.0	0.0	0.0	1.9	0.0	0.0	0.0	0.6	0 ± 0.001
Trabulsiella	0.0	0.0	0.0	25.0	0.0	1.2	1.1	9.6	0.0	0.0	0.0	0.2	0 ± 0.001
Tsukamurella	0.0	0.0	0.0	0.0	0.0	11.1	0.0	1.9	0.0	0.0	0.0	0.0	0 ± 0.001
Buttiauxella	2.8	0.0	0.0	0.0	0.0	8.6	0.0	5.8	0.0	0.0	0.0	0.0	0 ± 0.001
Adlercreutzia	0.0	0.0	0.0	0.0	0.0	0.0	0.0	0.0	0.0	0.0	0.0	1.2	0 ± 0.001
Alkaliphilus	0.0	0.0	0.0	0.0	0.0	0.0	1.1	0.0	0.0	0.0	0.0	0.8	0 ± 0.001
Labrys	0.0	0.0	0.0	0.0	0.0	2.5	0.0	0.0	0.0	0.0	0.0	0.4	0 ± 0.001
Microvirga	0.0	0.0	0.0	0.0	0.0	0.0	1.1	1.9	0.0	0.0	0.0	0.2	0 ± 0.001
Sandaracinus	0.0	0.0	0.0	0.0	0.0	0.0	0.0	0.0	0.0	0.0	0.0	0.2	0 ± 0.001
Thermodesulfovibrio	0.0	0.0	0.0	0.0	0.0	0.0	0.0	0.0	0.0	0.0	0.0	0.2	0 ± 0.001
Lampropedia	0.0	0.0	0.0	0.0	0.0	1.2	0.0	0.0	0.0	0.0	0.0	0.6	0 ± 0.001
Lishizhenia	0.0	0.0	0.0	0.0	0.0	6.2	0.0	3.8	0.0	0.0	0.0	0.0	0 ± 0.001
Alkanibacter	0.0	0.0	0.0	0.0	0.0	0.0	0.0	0.0	0.0	0.0	0.0	0.2	0 ± 0.001
Stomatobaculum	0.0	0.0	0.0	0.0	0.0	0.0	0.0	1.9	0.0	1.1	0.0	0.4	0 ± 0.001
Ramlibacter	0.0	0.0	0.0	0.0	0.0	4.9	0.0	0.0	0.0	0.0	0.0	0.4	0 ± 0.001
Turicella	0.0	0.0	0.0	0.0	0.0	0.0	0.0	0.0	0.0	1.1	0.0	0.0	0 ± 0.001

Sphingomicrobium	0.0	0.0	0.0	0.0	0.0	1.2	0.0	1.9	0.0	0.0	0.0	0.4	0 ± 0.001
Candidatus Accumulibacter	0.0	0.0	0.0	0.0	0.0	0.0	0.0	0.0	0.0	0.0	0.0	0.4	0 ± 0.001
Brachyspira	0.0	0.0	0.0	0.0	0.0	0.0	0.0	0.0	5.6	0.0	2.1	0.0	0 ± 0.001
Xanthobacter	0.0	0.0	0.0	0.0	0.0	1.2	0.0	0.0	0.0	0.0	0.0	0.2	0 ± 0.001
Solirubrobacter	0.0	0.0	0.0	0.0	0.0	6.2	1.1	0.0	0.0	0.0	0.0	0.0	0 ± 0.001
Aureimonas	0.0	0.0	0.0	0.0	0.0	3.7	1.1	0.0	0.0	0.0	0.0	0.0	0 ± 0.001
Lautropia	0.0	0.0	0.0	0.0	0.0	0.0	0.0	1.9	0.0	0.0	0.0	0.2	0 ± 0.001
Acidiphilium	0.0	0.0	0.0	0.0	0.0	6.2	0.0	0.0	0.0	0.0	0.0	0.0	0 ± 0.001
Commensalibacter	0.0	0.0	0.0	0.0	0.0	1.2	0.0	0.0	0.0	0.0	0.0	0.0	0 ± 0.001
Rhodanobacter	0.0	0.0	0.0	0.0	0.0	4.9	0.0	0.0	0.0	0.0	0.0	0.2	0 ± 0.001
GAL15	0.0	0.0	0.0	0.0	33.3	0.0	0.0	0.0	0.0	0.0	0.0	0.2	0 ± 0.001
Cellulosimicrobium sp. 137-9	2.8	0.0	0.0	0.0	0.0	0.0	0.0	0.0	0.0	0.0	0.0	0.0	0 ± 0.001
Lysobacter	0.0	0.0	0.0	0.0	0.0	3.7	0.0	3.8	0.0	0.0	0.0	0.2	0 ± 0.001
Rhodoplanes	0.0	0.0	0.0	0.0	0.0	4.9	2.2	0.0	0.0	0.0	0.0	0.2	0 ± 0
Subtercola	0.0	33.3	0.0	0.0	0.0	0.0	0.0	0.0	0.0	0.0	0.0	0.2	0 ± 0.001
Candidatus Rhabdochlamydia	0.0	0.0	0.0	0.0	0.0	1.2	0.0	0.0	0.0	0.0	0.0	0.2	0 ± 0.001
Orbus	0.0	0.0	0.0	0.0	0.0	1.2	0.0	0.0	0.0	0.0	0.0	0.0	0 ± 0.001
Planococcus	0.0	0.0	0.0	0.0	0.0	0.0	0.0	0.0	0.0	0.0	0.0	0.8	0 ± 0.001
Chitinimonas	0.0	0.0	0.0	0.0	0.0	0.0	1.1	0.0	0.0	0.0	0.0	0.0	0 ± 0.001
Rhizobacter	0.0	0.0	0.0	0.0	0.0	0.0	1.1	0.0	0.0	0.0	0.0	0.2	0 ± 0.001
Fodinicola	0.0	0.0	0.0	0.0	0.0	0.0	0.0	0.0	0.0	0.0	0.0	0.2	0 ± 0.001
Pseudokineococcus	0.0	0.0	0.0	0.0	0.0	0.0	0.0	0.0	0.0	0.0	0.0	0.2	0 ± 0.001
Neochlamydia	0.0	0.0	0.0	0.0	0.0	0.0	0.0	0.0	0.0	0.0	0.0	0.2	0 ± 0.001
Gallionella	0.0	0.0	0.0	0.0	0.0	0.0	0.0	0.0	0.0	0.0	0.0	0.2	0 ± 0.001
Rubritalea	0.0	0.0	0.0	0.0	0.0	3.7	0.0	5.8	0.0	0.0	0.0	0.0	0 ± 0.001
MWH-Ta3	0.0	0.0	0.0	0.0	0.0	0.0	0.0	0.0	0.0	0.0	0.0	0.4	0 ± 0.001
Pseudonocardia	0.0	0.0	0.0	0.0	0.0	0.0	3.3	3.8	0.0	0.0	0.0	0.0	0 ± 0.001
Chryseolinea	0.0	0.0	0.0	0.0	0.0	8.6	1.1	0.0	0.0	0.0	0.0	0.0	0 ± 0.001
Phormidium	0.0	0.0	0.0	0.0	0.0	0.0	1.1	0.0	0.0	0.0	0.0	0.0	0 ± 0.001
Desulfitobacterium	0.0	0.0	0.0	0.0	0.0	0.0	0.0	1.9	0.0	0.0	0.0	0.2	0 ± 0.001
Rhodoglobus	0.0	0.0	0.0	0.0	0.0	3.7	0.0	0.0	0.0	0.0	0.0	0.0	0 ± 0.001
Caldicellulosiruptor	0.0	0.0	0.0	0.0	0.0	0.0	0.0	0.0	0.0	1.1	0.0	0.2	0 ± 0.001
Proteocatella	0.0	0.0	0.0	0.0	0.0	0.0	0.0	1.9	0.0	0.0	0.0	0.2	0 ± 0.001
Sporichthya	0.0	0.0	0.0	0.0	0.0	0.0	0.0	1.9	0.0	0.0	0.0	0.2	0 ± 0.001
Brumimicrobium	0.0	0.0	0.0	0.0	0.0	0.0	0.0	0.0	0.0	0.0	0.0	0.2	0 ± 0.001
Chitinivorax	0.0	0.0	0.0	0.0	0.0	0.0	0.0	0.0	0.0	0.0	0.0	0.2	0 ± 0.001
Holdemania	0.0	0.0	0.0	0.0	0.0	0.0	0.0	15.4	0.0	0.0	0.0	0.4	0 ± 0
Phocoenobacter	0.0	0.0	0.0	0.0	0.0	1.2	0.0	0.0	0.0	0.0	0.0	0.0	0 ± 0.001
Rheinheimera	0.0	0.0	0.0	0.0	0.0	1.2	1.1	3.8	0.0	0.0	0.0	0.2	0 ± 0.001
OM60(NOR5) clade	0.0	0.0	0.0	0.0	0.0	1.2	0.0	0.0	0.0	0.0	0.0	0.2	0 ± 0.001
Parvibacter	0.0	0.0	0.0	0.0	0.0	1.2	0.0	0.0	0.0	0.0	0.0	0.2	0 ± 0.001
Shinella	0.0	0.0	0.0	25.0	0.0	1.2	0.0	0.0	0.0	0.0	0.0	0.2	0 ± 0.001
Octadecabacter	0.0	0.0	0.0	0.0	0.0	0.0	0.0	0.0	0.0	0.0	0.0	0.2	0 ± 0.001
Bordetella	2.8	0.0	0.0	0.0	0.0	0.0	0.0	1.9	0.0	0.0	0.0	0.0	0 ± 0.001
Yaniella	0.0	0.0	0.0	0.0	0.0	0.0	0.0	0.0	0.0	0.0	0.0	0.8	0 ± 0
Synechococcus sp. MA0607D	0.0	0.0	0.0	0.0	0.0	0.0	0.0	0.0	0.0	0.0	0.0	0.8	0 ± 0.001
Quadrisphaera	5.6	0.0	0.0	0.0	0.0	0.0	0.0	0.0	0.0	0.0	0.0	0.0	0 ± 0.001
Diplorickettsia	0.0	0.0	0.0	0.0	0.0	0.0	0.0	0.0	0.0	0.0	0.0	0.2	0 ± 0.001
Schlegelella	0.0	0.0	0.0	0.0	0.0	3.7	1.1	3.8	0.0	0.0	0.0	0.0	0 ± 0.001
Nesterenkonia	0.0	0.0	0.0	0.0	0.0	1.2	0.0	0.0	0.0	0.0	0.0	0.6	0 ± 0
Phocaeicola	0.0	0.0	0.0	0.0	0.0	0.0	0.0	0.0	0.0	0.0	0.0	0.6	0 ± 0.001
Catonella	2.8	0.0	0.0	0.0	0.0	0.0	0.0	7.7	0.0	0.0	0.0	0.4	0 ± 0.001
Xanthomonas	0.0	0.0	0.0	0.0	0.0	1.2	0.0	0.0	0.0	0.0	0.0	0.2	0 ± 0.001
Tepidimicrobium	0.0	0.0	0.0	0.0	0.0	0.0	0.0	0.0	0.0	0.0	0.0	0.6	0 ± 0
Dysgonomonas	0.0	0.0	0.0	0.0	0.0	1.2	0.0	1.9	0.0	0.0	0.0	0.6	0 ± 0
Actinotalea	0.0	0.0	0.0	0.0	0.0	1.2	1.1	1.9	0.0	0.0	0.0	0.0	0 ± 0.001
Anaerobacter	0.0	0.0	0.0	0.0	0.0	2.5	0.0	0.0	0.0	0.0	0.0	0.0	0 ± 0.001
Rikenella	0.0	0.0	0.0	0.0	0.0	0.0	0.0	0.0	0.0	0.0	0.0	0.6	0 ± 0
Bauldia	0.0	0.0	0.0	0.0	0.0	1.2	0.0	0.0	0.0	0.0	0.0	0.2	0 ± 0.001
Singulisphaera	0.0	0.0	0.0	0.0	0.0	1.2	0.0	0.0	0.0	0.0	0.0	0.2	0 ± 0.001
Filifactor	0.0	0.0	0.0	25.0	0.0	1.2	0.0	0.0	0.0	0.0	0.0	0.0	0 ± 0.001
Propionivibrio	0.0	0.0	0.0	0.0	0.0	1.2	0.0	5.8	0.0	0.0	0.0	0.2	0 ± 0.001
Phycicoccus	0.0	0.0	0.0	0.0	0.0	0.0	0.0	1.9	0.0	0.0	0.0	0.2	0 ± 0.001
Marinicella	0.0	0.0	0.0	0.0	0.0	6.2	0.0	0.0	0.0	0.0	0.0	0.0	0 ± 0
Herbiconiux	0.0	0.0	0.0	0.0	0.0	0.0	0.0	0.0	0.0	0.0	0.0	0.2	0 ± 0.001
Galbibacter	0.0	0.0	0.0	0.0	0.0	0.0	0.0	0.0	0.0	0.0	0.0	0.2	0 ± 0.001
Soonwooa	0.0	0.0	0.0	0.0	0.0	0.0	0.0	0.0	0.0	0.0	0.0	0.2	0 ± 0.001
Kaistia	0.0	0.0	0.0	0.0	0.0	0.0	0.0	0.0	0.0	0.0	0.0	0.2	0 ± 0.001
Natranaerovirga	0.0	0.0	0.0	0.0	0.0	0.0	0.0	11.5	0.0	0.0	0.0	0.2	0 ± 0
Edaphobacter	0.0	0.0	0.0	0.0	0.0	0.0	0.0	0.0	0.0	0.0	0.0	0.2	0 ± 0.001
Chroococcidiopsis	0.0	0.0	0.0	0.0	0.0	0.0	0.0	0.0	0.0	0.0	0.0	0.2	0 ± 0.001
Actibacterium	0.0	0.0	0.0	0.0	0.0	0.0	0.0	0.0	0.0	0.0	0.0	0.2	0 ± 0.001

Methylocaldum	0.0	0.0	0.0	0.0	0.0	0.0	0.0	0.0	0.0	0.0	0.0	0.2	0 ± 0.001
Turneriella	0.0	0.0	0.0	0.0	0.0	0.0	0.0	0.0	0.0	0.0	0.0	0.2	0 ± 0.001
Salinarimonas	0.0	0.0	0.0	0.0	0.0	2.5	0.0	0.0	0.0	0.0	0.0	0.0	0 ± 0.001
Rhodopila	0.0	0.0	0.0	0.0	0.0	0.0	0.0	0.0	0.0	0.0	0.0	0.2	0 ± 0.001
Macromonas	0.0	0.0	0.0	0.0	0.0	0.0	0.0	0.0	0.0	0.0	0.0	0.2	0 ± 0.001
Tepidicella	0.0	0.0	0.0	0.0	0.0	0.0	0.0	0.0	0.0	0.0	0.0	0.2	0 ± 0.001
Ferribacterium	0.0	0.0	0.0	0.0	0.0	0.0	0.0	0.0	0.0	0.0	0.0	0.2	0 ± 0.001
Candidatus Entotheonella	0.0	0.0	0.0	0.0	0.0	0.0	0.0	0.0	0.0	0.0	0.0	0.2	0 ± 0.001
Timonella senegalensis JC301	0.0	0.0	0.0	0.0	0.0	0.0	1.1	0.0	0.0	0.0	0.0	0.0	0 ± 0.001
Oryzihumus	0.0	0.0	0.0	0.0	0.0	1.2	1.1	11.5	0.0	0.0	0.0	0.0	0 ± 0
Nakamurella	2.8	0.0	0.0	0.0	0.0	2.5	1.1	0.0	0.0	0.0	0.0	0.0	0 ± 0
Chelatococcus	0.0	0.0	0.0	0.0	0.0	0.0	1.1	0.0	0.0	0.0	0.0	0.0	0 ± 0.001
Cellulosimicrobium	0.0	0.0	0.0	0.0	0.0	0.0	0.0	3.8	0.0	0.0	0.0	0.4	0 ± 0
Pleomorphomonas	0.0	0.0	0.0	0.0	0.0	0.0	0.0	0.0	0.0	0.0	0.0	0.2	0 ± 0.001
Propionimicrobium	0.0	0.0	0.0	0.0	0.0	0.0	2.2	0.0	0.0	0.0	0.0	0.0	0 ± 0
Dermatophilus	0.0	0.0	0.0	0.0	0.0	2.5	0.0	1.9	0.0	0.0	0.0	0.2	0 ± 0
Tomitella	0.0	0.0	0.0	0.0	0.0	0.0	1.1	0.0	0.0	0.0	0.0	0.0	0 ± 0.001
Gulbenkiania	0.0	0.0	0.0	0.0	0.0	0.0	1.1	0.0	0.0	0.0	0.0	0.0	0 ± 0.001
Candidatus Actinomarina	0.0	0.0	0.0	0.0	0.0	1.2	0.0	0.0	0.0	0.0	0.0	0.0	0 ± 0.001
Alcaligenes	2.8	0.0	0.0	0.0	0.0	0.0	0.0	0.0	0.0	0.0	0.0	0.0	0 ± 0.001
Candidatus Phytoplasma	2.8	0.0	0.0	0.0	0.0	0.0	0.0	0.0	0.0	0.0	0.0	0.0	0 ± 0.001
Denitrobacterium	0.0	0.0	0.0	0.0	0.0	0.0	0.0	0.0	0.0	0.0	2.1	0.0	0 ± 0.001
Halanaerobium	0.0	0.0	0.0	0.0	0.0	1.2	0.0	1.9	0.0	0.0	0.0	0.0	0 ± 0
Nicoletella	0.0	0.0	0.0	0.0	0.0	3.7	0.0	17.3	0.0	0.0	0.0	0.0	0 ± 0
Saccharibacillus	2.8	0.0	0.0	0.0	0.0	0.0	0.0	0.0	0.0	0.0	0.0	0.0	0 ± 0.001
Herminiimonas	0.0	0.0	0.0	0.0	0.0	4.9	0.0	1.9	0.0	0.0	0.0	0.0	0 ± 0
Rhodocyclus	0.0	0.0	0.0	0.0	0.0	7.4	0.0	0.0	0.0	0.0	0.0	0.0	0 ± 0
Magnetospirillum	2.8	0.0	0.0	0.0	0.0	0.0	0.0	0.0	0.0	0.0	0.0	0.0	0 ± 0.001
Citricoccus	0.0	0.0	0.0	0.0	0.0	0.0	0.0	0.0	0.0	0.0	0.0	0.2	0 ± 0.001
Gelidibacter	0.0	0.0	0.0	0.0	0.0	0.0	0.0	0.0	0.0	0.0	0.0	0.2	0 ± 0.001
Empedobacter	0.0	0.0	0.0	0.0	0.0	1.2	1.1	0.0	0.0	0.0	0.0	0.0	0 ± 0
Micropruina	0.0	0.0	0.0	0.0	0.0	2.5	0.0	0.0	0.0	0.0	0.0	0.0	0 ± 0
Alysiella	0.0	0.0	0.0	0.0	0.0	0.0	0.0	1.9	0.0	0.0	0.0	0.2	0 ± 0.001
Sphaerotilus	2.8	0.0	0.0	0.0	0.0	0.0	0.0	0.0	0.0	0.0	0.0	0.0	0 ± 0.001
Tropheryma	0.0	0.0	0.0	0.0	0.0	0.0	1.1	0.0	0.0	0.0	0.0	0.0	0 ± 0.001
Sporomusa	0.0	0.0	0.0	0.0	0.0	0.0	1.1	0.0	0.0	0.0	0.0	0.0	0 ± 0
Persicitalea	0.0	0.0	0.0	0.0	0.0	0.0	0.0	0.0	0.0	0.0	0.0	0.2	0 ± 0
Oceanobacter	0.0	0.0	0.0	0.0	0.0	0.0	0.0	0.0	0.0	0.0	0.0	0.2	0 ± 0
Prauserella	0.0	0.0	0.0	0.0	0.0	6.2	0.0	0.0	0.0	0.0	0.0	0.2	0 ± 0
Sodalis glossinidius	0.0	0.0	0.0	0.0	0.0	0.0	1.1	0.0	0.0	0.0	0.0	0.0	0 ± 0
Vitreoscilla	0.0	0.0	0.0	0.0	0.0	1.2	2.2	0.0	0.0	0.0	0.0	0.0	0 ± 0
Propioniciclava	2.8	0.0	0.0	0.0	0.0	0.0	1.1	3.8	0.0	0.0	0.0	0.0	0 ± 0
Dickeya	2.8	0.0	0.0	0.0	0.0	1.2	0.0	1.9	0.0	0.0	0.0	0.0	0 ± 0
Pseudoxanthomonas	0.0	0.0	0.0	0.0	0.0	1.2	0.0	1.9	0.0	0.0	0.0	0.2	0 ± 0
Nesiotobacter	2.8	0.0	0.0	0.0	0.0	0.0	1.1	0.0	0.0	0.0	0.0	0.0	0 ± 0
Aeriscardovia	0.0	0.0	0.0	0.0	0.0	0.0	0.0	0.0	0.0	1.1	0.0	0.0	0 ± 0
Nocardiopsis	0.0	0.0	0.0	0.0	0.0	0.0	0.0	0.0	0.0	0.0	0.0	0.2	0 ± 0
Frateuria	0.0	0.0	0.0	0.0	0.0	0.0	1.1	0.0	0.0	0.0	0.0	0.0	0 ± 0
Plantibacter	2.8	0.0	0.0	0.0	0.0	0.0	0.0	0.0	0.0	0.0	0.0	0.0	0 ± 0
Leifsonia	0.0	0.0	0.0	25.0	0.0	1.2	0.0	0.0	0.0	0.0	0.0	0.0	0 ± 0
Amphibacillus	0.0	0.0	0.0	0.0	0.0	0.0	0.0	0.0	0.0	0.0	0.0	0.4	0 ± 0
BAL58 marine group	0.0	0.0	0.0	0.0	0.0	1.2	0.0	0.0	0.0	0.0	0.0	0.2	0 ± 0
Paucibacter	0.0	0.0	0.0	0.0	0.0	1.2	0.0	0.0	0.0	0.0	0.0	0.2	0 ± 0
Niastella	0.0	0.0	0.0	0.0	0.0	1.2	0.0	0.0	0.0	0.0	0.0	0.2	0 ± 0
Methylocella	0.0	0.0	0.0	0.0	0.0	6.2	0.0	0.0	0.0	0.0	0.0	0.0	0 ± 0
Chlamydia	0.0	0.0	0.0	0.0	0.0	0.0	0.0	0.0	0.0	0.0	0.0	0.4	0 ± 0
Enteractinococcus	0.0	0.0	0.0	0.0	0.0	0.0	0.0	0.0	0.0	0.0	0.0	0.4	0 ± 0
Marinilactibacillus	0.0	0.0	0.0	0.0	0.0	0.0	0.0	0.0	0.0	0.0	0.0	0.6	0 ± 0
Meganema	0.0	0.0	0.0	0.0	0.0	0.0	2.2	0.0	0.0	0.0	0.0	0.0	0 ± 0
Parasegetibacter	0.0	0.0	0.0	0.0	0.0	1.2	0.0	0.0	0.0	0.0	0.0	0.0	0 ± 0
Meiothermus	0.0	0.0	0.0	0.0	0.0	1.2	0.0	0.0	0.0	1.1	0.0	0.0	0 ± 0
Ornithinicoccus	0.0	0.0	0.0	0.0	0.0	1.2	1.1	0.0	0.0	0.0	0.0	0.0	0 ± 0
Loktanella	0.0	0.0	0.0	0.0	0.0	0.0	1.1	0.0	0.0	0.0	0.0	0.0	0 ± 0
Rhodoblastus	0.0	0.0	0.0	0.0	0.0	2.5	0.0	0.0	0.0	0.0	0.0	0.0	0 ± 0
Planifilum	0.0	0.0	0.0	0.0	0.0	4.9	0.0	0.0	0.0	0.0	0.0	0.0	0 ± 0
Candidatus Rhodoluna	0.0	0.0	0.0	0.0	0.0	0.0	0.0	1.9	0.0	0.0	0.0	0.2	0 ± 0
Silanimonas	0.0	0.0	0.0	0.0	0.0	4.9	0.0	1.9	0.0	0.0	0.0	0.0	0 ± 0
Pelotomaculum	0.0	0.0	0.0	0.0	0.0	0.0	0.0	1.9	0.0	0.0	0.0	0.2	0 ± 0
Gordonibacter	0.0	0.0	0.0	0.0	0.0	0.0	0.0	3.8	0.0	0.0	0.0	0.2	0 ± 0
Terracoccus	0.0	0.0	0.0	0.0	0.0	0.0	0.0	0.0	0.0	0.0	0.0	0.2	0 ± 0
Chitinophaga	0.0	0.0	0.0	0.0	0.0	0.0	0.0	0.0	0.0	0.0	0.0	0.2	0 ± 0
Segetibacter	0.0	0.0	0.0	0.0	0.0	0.0	0.0	0.0	0.0	0.0	0.0	0.2	0 ± 0

Candidatus Aquirestis	0.0	0.0	0.0	0.0	0.0	0.0	0.0	0.0	0.0	0.0	0.0	0.2	0 ± 0
Mucilaginibacter	0.0	0.0	0.0	0.0	0.0	0.0	0.0	0.0	0.0	0.0	0.0	0.2	0 ± 0
Levilinea	0.0	0.0	0.0	0.0	0.0	0.0	0.0	0.0	0.0	0.0	0.0	0.2	0 ± 0
Fictibacillus	0.0	0.0	0.0	0.0	0.0	0.0	0.0	0.0	0.0	0.0	0.0	0.2	0 ± 0
Candidatus Methylomirabilis	0.0	0.0	0.0	0.0	0.0	0.0	0.0	0.0	0.0	0.0	0.0	0.2	0 ± 0
Oceaniovalibus	0.0	0.0	0.0	0.0	0.0	0.0	0.0	0.0	0.0	0.0	0.0	0.2	0 ± 0
Rhodovastum	0.0	0.0	0.0	0.0	0.0	0.0	0.0	0.0	0.0	0.0	0.0	0.2	0 ± 0
Ferriphaselus	0.0	0.0	0.0	0.0	0.0	0.0	0.0	0.0	0.0	0.0	0.0	0.2	0 ± 0
Candidatus Branchiomonas	0.0	0.0	0.0	0.0	0.0	0.0	0.0	0.0	0.0	0.0	0.0	0.2	0 ± 0
Desulforegula	0.0	0.0	0.0	0.0	0.0	0.0	0.0	0.0	0.0	0.0	0.0	0.2	0 ± 0
SEEP-SRB4	0.0	0.0	0.0	0.0	0.0	0.0	0.0	0.0	0.0	0.0	0.0	0.2	0 ± 0
Geothermobacter	0.0	0.0	0.0	0.0	0.0	0.0	0.0	0.0	0.0	0.0	0.0	0.2	0 ± 0
Syntrophus	0.0	0.0	0.0	0.0	0.0	0.0	0.0	0.0	0.0	0.0	0.0	0.2	0 ± 0
Croceicoccus	0.0	0.0	0.0	0.0	0.0	0.0	1.1	0.0	0.0	0.0	0.0	0.0	0 ± 0
Pontibacter	0.0	0.0	0.0	0.0	0.0	1.2	0.0	0.0	0.0	1.1	0.0	0.0	0 ± 0
Advenella	0.0	0.0	0.0	0.0	0.0	0.0	0.0	0.0	0.0	0.0	0.0	0.4	0 ± 0
Gulosibacter	0.0	0.0	0.0	0.0	0.0	0.0	1.1	0.0	0.0	0.0	0.0	0.0	0 ± 0
Algoriphagus	0.0	0.0	0.0	0.0	0.0	0.0	1.1	0.0	0.0	0.0	0.0	0.0	0 ± 0
Piscicoccus	0.0	0.0	0.0	0.0	0.0	2.5	0.0	1.9	0.0	0.0	0.0	0.0	0 ± 0
Thermoanaerobacter	0.0	0.0	0.0	0.0	0.0	3.7	0.0	1.9	0.0	0.0	0.0	0.0	0 ± 0
Chromohalobacter	2.8	0.0	0.0	0.0	0.0	0.0	0.0	0.0	0.0	0.0	0.0	0.0	0 ± 0
Pannonibacter	0.0	0.0	0.0	0.0	0.0	4.9	0.0	0.0	0.0	0.0	0.0	0.0	0 ± 0
Ornithinimicrobium	0.0	0.0	0.0	0.0	0.0	0.0	0.0	1.9	0.0	0.0	0.0	0.2	0 ± 0
Rubellimicrobium	0.0	0.0	0.0	0.0	0.0	2.5	0.0	3.8	0.0	0.0	0.0	0.0	0 ± 0
Streptacidiphilus	0.0	0.0	0.0	0.0	0.0	0.0	0.0	0.0	0.0	0.0	0.0	0.2	0 ± 0
Brevifollis	0.0	0.0	0.0	0.0	0.0	0.0	0.0	0.0	0.0	0.0	0.0	0.2	0 ± 0
Acetobacter	0.0	0.0	0.0	0.0	0.0	2.5	0.0	1.9	0.0	2.2	0.0	0.0	0 ± 0
Marinobacterium	0.0	0.0	0.0	0.0	0.0	3.7	0.0	0.0	0.0	0.0	0.0	0.0	0 ± 0
Peredibacter	0.0	0.0	0.0	0.0	0.0	0.0	0.0	1.9	0.0	0.0	0.0	0.0	0 ± 0
Actinomadura	0.0	0.0	0.0	0.0	0.0	1.2	0.0	5.8	0.0	0.0	0.0	0.0	0 ± 0
Granulicatella	0.0	0.0	0.0	0.0	0.0	0.0	0.0	0.0	0.0	0.0	0.0	0.2	0 ± 0
Methylophaga	0.0	0.0	0.0	0.0	0.0	0.0	0.0	1.9	0.0	0.0	0.0	0.2	0 ± 0
Pilimelia	0.0	0.0	0.0	0.0	0.0	0.0	1.1	3.8	0.0	0.0	0.0	0.0	0 ± 0
Acidisoma	0.0	0.0	0.0	0.0	0.0	2.5	0.0	0.0	0.0	0.0	0.0	0.0	0 ± 0
Litoribacillus	0.0	0.0	0.0	0.0	0.0	1.2	0.0	1.9	0.0	0.0	0.0	0.0	0 ± 0
Truepera	0.0	0.0	0.0	0.0	0.0	1.2	0.0	0.0	0.0	0.0	0.0	0.2	0 ± 0
Tetragenococcus	0.0	0.0	0.0	0.0	0.0	0.0	0.0	0.0	0.0	0.0	0.0	0.2	0 ± 0
Mesoplasma	0.0	0.0	0.0	0.0	0.0	1.2	0.0	0.0	0.0	0.0	0.0	0.0	0 ± 0
Helcobacillus	0.0	0.0	0.0	0.0	0.0	0.0	0.0	0.0	0.0	0.0	0.0	0.2	0 ± 0
Dolosicoccus	0.0	0.0	0.0	0.0	0.0	1.2	0.0	0.0	0.0	0.0	0.0	0.0	0 ± 0
Leminorella	0.0	0.0	0.0	0.0	0.0	0.0	1.1	0.0	0.0	0.0	0.0	0.0	0 ± 0
Demequina	0.0	0.0	0.0	0.0	0.0	0.0	1.1	0.0	0.0	0.0	0.0	0.0	0 ± 0
Steroidobacter	0.0	0.0	0.0	0.0	0.0	0.0	1.1	0.0	0.0	0.0	0.0	0.0	0 ± 0
Verrucomicrobium	0.0	0.0	0.0	0.0	0.0	0.0	1.1	0.0	0.0	0.0	0.0	0.0	0 ± 0
Thermoactinomyces	0.0	0.0	0.0	0.0	0.0	0.0	0.0	0.0	0.0	0.0	0.0	0.2	0 ± 0
Rhodobacter sp. JA431	0.0	0.0	0.0	0.0	0.0	0.0	1.1	0.0	0.0	0.0	0.0	0.0	0 ± 0
Aquimonas	0.0	0.0	0.0	0.0	0.0	0.0	0.0	0.0	0.0	0.0	0.0	0.2	0 ± 0
Asticcacaulis	0.0	0.0	0.0	0.0	0.0	0.0	1.1	0.0	0.0	0.0	0.0	0.0	0 ± 0
Parapedobacter	0.0	0.0	0.0	0.0	0.0	3.7	0.0	0.0	0.0	0.0	0.0	0.0	0 ± 0
Luminiphilus	0.0	0.0	0.0	0.0	0.0	1.2	0.0	0.0	0.0	0.0	0.0	0.0	0 ± 0
Owenweeksia	0.0	0.0	0.0	0.0	0.0	3.7	0.0	0.0	0.0	0.0	0.0	0.0	0 ± 0
Mesonia	0.0	0.0	0.0	0.0	0.0	1.2	0.0	0.0	0.0	0.0	0.0	0.0	0 ± 0
Roseobacter clade NAC11-7 lineage	0.0	0.0	0.0	0.0	0.0	1.2	0.0	0.0	0.0	0.0	0.0	0.0	0 ± 0
Desulfotignum	0.0	0.0	0.0	0.0	0.0	1.2	0.0	0.0	0.0	0.0	0.0	0.0	0 ± 0
Dasania	0.0	0.0	0.0	0.0	0.0	2.5	0.0	0.0	0.0	0.0	0.0	0.0	0 ± 0
Castellaniella	0.0	0.0	0.0	0.0	0.0	2.5	0.0	0.0	0.0	0.0	0.0	0.0	0 ± 0
Idiomarina	0.0	0.0	0.0	0.0	0.0	2.5	0.0	0.0	0.0	0.0	0.0	0.0	0 ± 0
Saccharomonospora	0.0	0.0	0.0	0.0	0.0	2.5	0.0	0.0	0.0	0.0	0.0	0.0	0 ± 0
Niabella	0.0	0.0	0.0	0.0	0.0	0.0	0.0	1.9	0.0	0.0	0.0	0.0	0 ± 0
Thalassolituus	0.0	0.0	0.0	0.0	0.0	0.0	0.0	1.9	0.0	0.0	0.0	0.0	0 ± 0
Photobacterium	0.0	0.0	0.0	0.0	0.0	0.0	0.0	1.9	0.0	0.0	0.0	0.0	0 ± 0
NS3a marine group	0.0	0.0	0.0	0.0	0.0	0.0	0.0	1.9	0.0	0.0	0.0	0.0	0 ± 0
Sphaerobacter	0.0	0.0	0.0	0.0	0.0	0.0	0.0	1.9	0.0	0.0	0.0	0.0	0 ± 0
Thermaerobacter	0.0	0.0	0.0	0.0	0.0	0.0	0.0	1.9	0.0	0.0	0.0	0.0	0 ± 0
Ohtaekwangia	0.0	0.0	0.0	0.0	0.0	2.5	0.0	0.0	0.0	0.0	0.0	0.0	0 ± 0
Dolosigranulum	0.0	0.0	0.0	0.0	0.0	2.5	0.0	0.0	0.0	0.0	0.0	0.0	0 ± 0
Marinococcus	0.0	0.0	0.0	0.0	0.0	1.2	0.0	0.0	0.0	0.0	0.0	0.0	0 ± 0
Oerskovia	0.0	0.0	0.0	0.0	0.0	0.0	0.0	0.0	0.0	0.0	0.0	0.2	0 ± 0
Jonesia	0.0	0.0	0.0	0.0	0.0	0.0	0.0	0.0	0.0	0.0	0.0	0.2	0 ± 0
Plantactinospora	0.0	0.0	0.0	0.0	0.0	0.0	0.0	0.0	0.0	0.0	0.0	0.2	0 ± 0
Atopococcus	0.0	0.0	0.0	0.0	0.0	0.0	0.0	0.0	0.0	0.0	0.0	0.2	0 ± 0
Pseudofulvimonas	0.0	0.0	0.0	0.0	0.0	0.0	0.0	0.0	0.0	0.0	0.0	0.2	0 ± 0

Aeribacillus	0.0	0.0	0.0	0.0	0.0	2.5	0.0	0.0	0.0	0.0	0.0	0.0	0 ± 0
Telmatobacter	0.0	0.0	0.0	0.0	0.0	1.2	0.0	0.0	0.0	0.0	0.0	0.0	0 ± 0
Oxalophagus	0.0	0.0	0.0	0.0	0.0	1.2	0.0	0.0	0.0	0.0	0.0	0.0	0 ± 0
Filomicrobium	0.0	0.0	0.0	0.0	0.0	1.2	0.0	0.0	0.0	0.0	0.0	0.0	0 ± 0
Leisingera	0.0	0.0	0.0	0.0	0.0	1.2	0.0	0.0	0.0	0.0	0.0	0.0	0 ± 0
Saccharibacter	0.0	0.0	0.0	0.0	0.0	1.2	0.0	0.0	0.0	0.0	0.0	0.0	0 ± 0
Caldimonas	0.0	0.0	0.0	0.0	0.0	1.2	0.0	0.0	0.0	0.0	0.0	0.0	0 ± 0
Phaselicystis	0.0	0.0	0.0	0.0	0.0	1.2	0.0	0.0	0.0	0.0	0.0	0.0	0 ± 0
Kiloniella	0.0	0.0	0.0	0.0	0.0	2.5	0.0	0.0	0.0	0.0	0.0	0.0	0 ± 0
Photorhabdus luminescens	0.0	0.0	0.0	0.0	0.0	2.5	0.0	0.0	0.0	0.0	0.0	0.0	0 ± 0
Oceanobacillus	0.0	0.0	0.0	0.0	0.0	0.0	0.0	0.0	0.0	0.0	0.0	0.2	0 ± 0
Coprobacter	0.0	0.0	0.0	0.0	0.0	1.2	0.0	1.9	0.0	0.0	0.0	0.0	0 ± 0
Yonghaparkia	0.0	0.0	0.0	0.0	0.0	1.2	0.0	0.0	0.0	0.0	0.0	0.0	0 ± 0
Fontibacter	0.0	0.0	0.0	0.0	0.0	1.2	0.0	0.0	0.0	0.0	0.0	0.0	0 ± 0
Extensimonas	0.0	0.0	0.0	0.0	0.0	1.2	0.0	0.0	0.0	0.0	0.0	0.0	0 ± 0
Alteromonas	0.0	0.0	0.0	0.0	0.0	1.2	0.0	0.0	0.0	0.0	0.0	0.0	0 ± 0
Bisgaardia	0.0	0.0	0.0	0.0	0.0	1.2	0.0	0.0	0.0	0.0	0.0	0.0	0 ± 0
Bergeriella	0.0	0.0	0.0	0.0	0.0	1.2	0.0	0.0	0.0	0.0	0.0	0.0	0 ± 0
Brevibacillus	0.0	0.0	0.0	0.0	0.0	2.5	0.0	0.0	0.0	0.0	0.0	0.0	0 ± 0
Falsirhodobacter	0.0	0.0	0.0	0.0	0.0	0.0	0.0	1.9	0.0	0.0	0.0	0.0	0 ± 0
Iodobacter	0.0	0.0	0.0	0.0	0.0	0.0	0.0	0.0	0.0	0.0	0.0	0.2	0 ± 0
Camelimonas	0.0	0.0	0.0	0.0	0.0	1.2	0.0	0.0	0.0	0.0	0.0	0.0	0 ± 0
Amaricoccus	0.0	0.0	0.0	0.0	0.0	1.2	0.0	0.0	0.0	0.0	0.0	0.0	0 ± 0
Catenulispora	0.0	0.0	0.0	0.0	0.0	1.2	0.0	0.0	0.0	0.0	0.0	0.0	0 ± 0
Bogoriella	0.0	0.0	0.0	0.0	0.0	1.2	0.0	0.0	0.0	0.0	0.0	0.0	0 ± 0
Branchiibius	0.0	0.0	0.0	0.0	0.0	1.2	0.0	0.0	0.0	0.0	0.0	0.0	0 ± 0
Serinicoccus	0.0	0.0	0.0	0.0	0.0	1.2	0.0	0.0	0.0	0.0	0.0	0.0	0 ± 0
Albibacter	0.0	0.0	0.0	0.0	0.0	1.2	0.0	0.0	0.0	0.0	0.0	0.0	0 ± 0
Uliginosibacterium	0.0	0.0	0.0	0.0	0.0	1.2	0.0	0.0	0.0	0.0	0.0	0.0	0 ± 0
Chungangia	0.0	0.0	0.0	0.0	0.0	0.0	0.0	0.0	0.0	0.0	0.0	0.2	0 ± 0
Jonquetella	0.0	0.0	0.0	0.0	0.0	0.0	0.0	5.8	0.0	0.0	0.0	0.0	0 ± 0
Aquitalea	0.0	0.0	0.0	0.0	0.0	1.2	0.0	1.9	0.0	0.0	0.0	0.0	0 ± 0
Modestobacter	0.0	0.0	0.0	0.0	0.0	1.2	0.0	0.0	0.0	0.0	0.0	0.0	0 ± 0
Crocinitomix	0.0	0.0	0.0	0.0	0.0	1.2	0.0	0.0	0.0	0.0	0.0	0.0	0 ± 0
Caldithrix	0.0	0.0	0.0	0.0	0.0	1.2	0.0	0.0	0.0	0.0	0.0	0.0	0 ± 0
Aliihoeflea	0.0	0.0	0.0	0.0	0.0	1.2	0.0	0.0	0.0	0.0	0.0	0.0	0 ± 0
Propionigenium	0.0	0.0	0.0	0.0	0.0	0.0	0.0	3.8	0.0	0.0	0.0	0.0	0 ± 0
Salana	0.0	0.0	0.0	0.0	0.0	1.2	0.0	0.0	0.0	0.0	0.0	0.0	0 ± 0
Seohaeicola	0.0	0.0	0.0	0.0	0.0	1.2	0.0	0.0	0.0	0.0	0.0	0.0	0 ± 0
Pseudacidovorax	0.0	0.0	0.0	0.0	0.0	1.2	0.0	0.0	0.0	0.0	0.0	0.0	0 ± 0
Plasticicumulans	0.0	0.0	0.0	0.0	0.0	1.2	0.0	0.0	0.0	0.0	0.0	0.0	0 ± 0
Kineosphaera	0.0	0.0	0.0	0.0	0.0	1.2	0.0	0.0	0.0	0.0	0.0	0.0	0 ± 0
Euzebya	0.0	0.0	0.0	0.0	0.0	1.2	0.0	0.0	0.0	0.0	0.0	0.0	0 ± 0
Salinibacter	0.0	0.0	0.0	0.0	0.0	1.2	0.0	0.0	0.0	0.0	0.0	0.0	0 ± 0
NS4 marine group	0.0	0.0	0.0	0.0	0.0	1.2	0.0	0.0	0.0	0.0	0.0	0.0	0 ± 0
NS5 marine group	0.0	0.0	0.0	0.0	0.0	1.2	0.0	0.0	0.0	0.0	0.0	0.0	0 ± 0
Pir4 lineage	0.0	0.0	0.0	0.0	0.0	1.2	0.0	0.0	0.0	0.0	0.0	0.0	0 ± 0
Martelella	0.0	0.0	0.0	0.0	0.0	1.2	0.0	0.0	0.0	0.0	0.0	0.0	0 ± 0
Daeguia	0.0	0.0	0.0	0.0	0.0	1.2	0.0	0.0	0.0	0.0	0.0	0.0	0 ± 0
Labrenzia	0.0	0.0	0.0	0.0	0.0	1.2	0.0	0.0	0.0	0.0	0.0	0.0	0 ± 0
Pseudorhodobacter	0.0	0.0	0.0	0.0	0.0	1.2	0.0	0.0	0.0	0.0	0.0	0.0	0 ± 0
Roseinatronobacter	0.0	0.0	0.0	0.0	0.0	1.2	0.0	0.0	0.0	0.0	0.0	0.0	0 ± 0
Rubritepida	0.0	0.0	0.0	0.0	0.0	1.2	0.0	0.0	0.0	0.0	0.0	0.0	0 ± 0
AEGEAN-169 marine group	0.0	0.0	0.0	0.0	0.0	1.2	0.0	0.0	0.0	0.0	0.0	0.0	0 ± 0
OM27 clade	0.0	0.0	0.0	0.0	0.0	1.2	0.0	0.0	0.0	0.0	0.0	0.0	0 ± 0
Sva0081 sediment group	0.0	0.0	0.0	0.0	0.0	1.2	0.0	0.0	0.0	0.0	0.0	0.0	0 ± 0
Haliea	0.0	0.0	0.0	0.0	0.0	1.2	0.0	0.0	0.0	0.0	0.0	0.0	0 ± 0
SAR92 clade	0.0	0.0	0.0	0.0	0.0	1.2	0.0	0.0	0.0	0.0	0.0	0.0	0 ± 0
Candidatus Hamiltonella	0.0	0.0	0.0	0.0	0.0	1.2	0.0	0.0	0.0	0.0	0.0	0.0	0 ± 0
Balneatrix	0.0	0.0	0.0	0.0	0.0	1.2	0.0	0.0	0.0	0.0	0.0	0.0	0 ± 0
Neptuniibacter	0.0	0.0	0.0	0.0	0.0	1.2	0.0	0.0	0.0	0.0	0.0	0.0	0 ± 0
Mariprofundus	0.0	0.0	0.0	0.0	0.0	1.2	0.0	0.0	0.0	0.0	0.0	0.0	0 ± 0
Micromonospora	0.0	0.0	0.0	0.0	0.0	0.0	0.0	5.8	0.0	0.0	0.0	0.0	0 ± 0
Thermicanus	0.0	0.0	0.0	0.0	0.0	1.2	0.0	0.0	0.0	0.0	0.0	0.0	0 ± 0
AKYG587	0.0	0.0	0.0	0.0	0.0	1.2	0.0	0.0	0.0	0.0	0.0	0.0	0 ± 0
Enhygromyxa	0.0	0.0	0.0	0.0	0.0	1.2	0.0	0.0	0.0	0.0	0.0	0.0	0 ± 0
Catenococcus	0.0	0.0	0.0	0.0	0.0	1.2	0.0	0.0	0.0	0.0	0.0	0.0	0 ± 0
Amycolatopsis	0.0	0.0	0.0	0.0	0.0	0.0	0.0	7.7	0.0	0.0	0.0	0.0	0 ± 0
Actinoplanes	0.0	0.0	0.0	0.0	0.0	0.0	0.0	5.8	0.0	0.0	0.0	0.0	0 ± 0
Dactylosporangium	0.0	0.0	0.0	0.0	0.0	1.2	0.0	0.0	0.0	0.0	0.0	0.0	0 ± 0
Thermobifida	0.0	0.0	0.0	0.0	0.0	1.2	0.0	0.0	0.0	0.0	0.0	0.0	0 ± 0
Candidatus Xiphinematobacter	0.0	0.0	0.0	0.0	0.0	0.0	0.0	0.0	0.0	1.1	0.0	0.0	0 ± 0

Acetivibrio	0.0	0.0	0.0	0.0	0.0	0.0	0.0	5.8	0.0	0.0	0.0	0.0	0 ± 0
Sulfurospirillum	0.0	0.0	0.0	0.0	0.0	0.0	0.0	3.8	0.0	0.0	0.0	0.0	0 ± 0
Defluviitalea	0.0	0.0	0.0	0.0	0.0	0.0	0.0	5.8	0.0	0.0	0.0	0.0	0 ± 0
Dehalococcoides	0.0	0.0	0.0	0.0	0.0	0.0	0.0	1.9	0.0	0.0	0.0	0.0	0 ± 0
Aeromicrobium	0.0	0.0	0.0	0.0	0.0	0.0	0.0	3.8	0.0	0.0	0.0	0.0	0 ± 0
Actinokineospora	0.0	0.0	0.0	0.0	0.0	0.0	0.0	3.8	0.0	0.0	0.0	0.0	0 ± 0
Aestuariimicrobium	0.0	0.0	0.0	0.0	0.0	0.0	0.0	3.8	0.0	0.0	0.0	0.0	0 ± 0
Lutispora	0.0	0.0	0.0	0.0	0.0	0.0	0.0	3.8	0.0	0.0	0.0	0.0	0 ± 0
Oxobacter	0.0	0.0	0.0	0.0	0.0	0.0	0.0	3.8	0.0	0.0	0.0	0.0	0 ± 0
Incertae Sedis	0.0	0.0	0.0	0.0	0.0	0.0	0.0	3.8	0.0	0.0	0.0	0.0	0 ± 0
Dermabacter	0.0	0.0	0.0	0.0	0.0	0.0	0.0	1.9	0.0	0.0	0.0	0.0	0 ± 0
Chthonomonas	0.0	0.0	0.0	0.0	0.0	0.0	0.0	1.9	0.0	0.0	0.0	0.0	0 ± 0
Serinibacter	0.0	0.0	0.0	0.0	0.0	0.0	0.0	1.9	0.0	0.0	0.0	0.0	0 ± 0
Nonomuraea	0.0	0.0	0.0	0.0	0.0	0.0	0.0	1.9	0.0	0.0	0.0	0.0	0 ± 0
Sporacetigenium	0.0	0.0	0.0	0.0	0.0	0.0	0.0	1.9	0.0	0.0	0.0	0.0	0 ± 0
Sulfurovum	0.0	0.0	0.0	0.0	0.0	0.0	0.0	1.9	0.0	0.0	0.0	0.0	0 ± 0
Dickeya zeae	0.0	0.0	0.0	0.0	0.0	0.0	0.0	1.9	0.0	0.0	0.0	0.0	0 ± 0
Kibdelosporangium	0.0	0.0	0.0	0.0	0.0	0.0	0.0	1.9	0.0	0.0	0.0	0.0	0 ± 0
Ethanoligenens	0.0	0.0	0.0	0.0	0.0	0.0	0.0	1.9	0.0	0.0	0.0	0.0	0 ± 0
Dethiosulfovibrio	0.0	0.0	0.0	0.0	0.0	0.0	0.0	1.9	0.0	0.0	0.0	0.0	0 ± 0
Arsenicicoccus	0.0	0.0	0.0	0.0	0.0	0.0	0.0	1.9	0.0	0.0	0.0	0.0	0 ± 0
Promicromonospora	0.0	0.0	0.0	0.0	0.0	0.0	0.0	1.9	0.0	0.0	0.0	0.0	0 ± 0
Anaerosphaera	0.0	0.0	0.0	0.0	0.0	0.0	0.0	1.9	0.0	0.0	0.0	0.0	0 ± 0
Xylophilus	0.0	0.0	0.0	0.0	0.0	0.0	0.0	1.9	0.0	0.0	0.0	0.0	0 ± 0
Acidothermus	0.0	0.0	0.0	0.0	0.0	0.0	0.0	1.9	0.0	0.0	0.0	0.0	0 ± 0
Geodermatophilus	0.0	0.0	0.0	0.0	0.0	0.0	0.0	1.9	0.0	0.0	0.0	0.0	0 ± 0
Motilibacter	0.0	0.0	0.0	0.0	0.0	0.0	0.0	1.9	0.0	0.0	0.0	0.0	0 ± 0
Ammoniphilus	0.0	0.0	0.0	0.0	0.0	0.0	0.0	1.9	0.0	0.0	0.0	0.0	0 ± 0
Avibacterium	0.0	0.0	0.0	0.0	0.0	0.0	0.0	1.9	0.0	0.0	0.0	0.0	0 ± 0
Soehngenia	0.0	0.0	0.0	0.0	0.0	0.0	0.0	1.9	0.0	0.0	0.0	0.0	0 ± 0
