# Supplementary figures and images for: Meta-analysis To Define a Core Microbiota in the Swine Gut
Source: mSystems. 2017 May 23;2(3):e00004-17. doi: 10.1128/mSystems.00004-17 (PMC5443231; doi:10.1128/mSystems.00004-17)

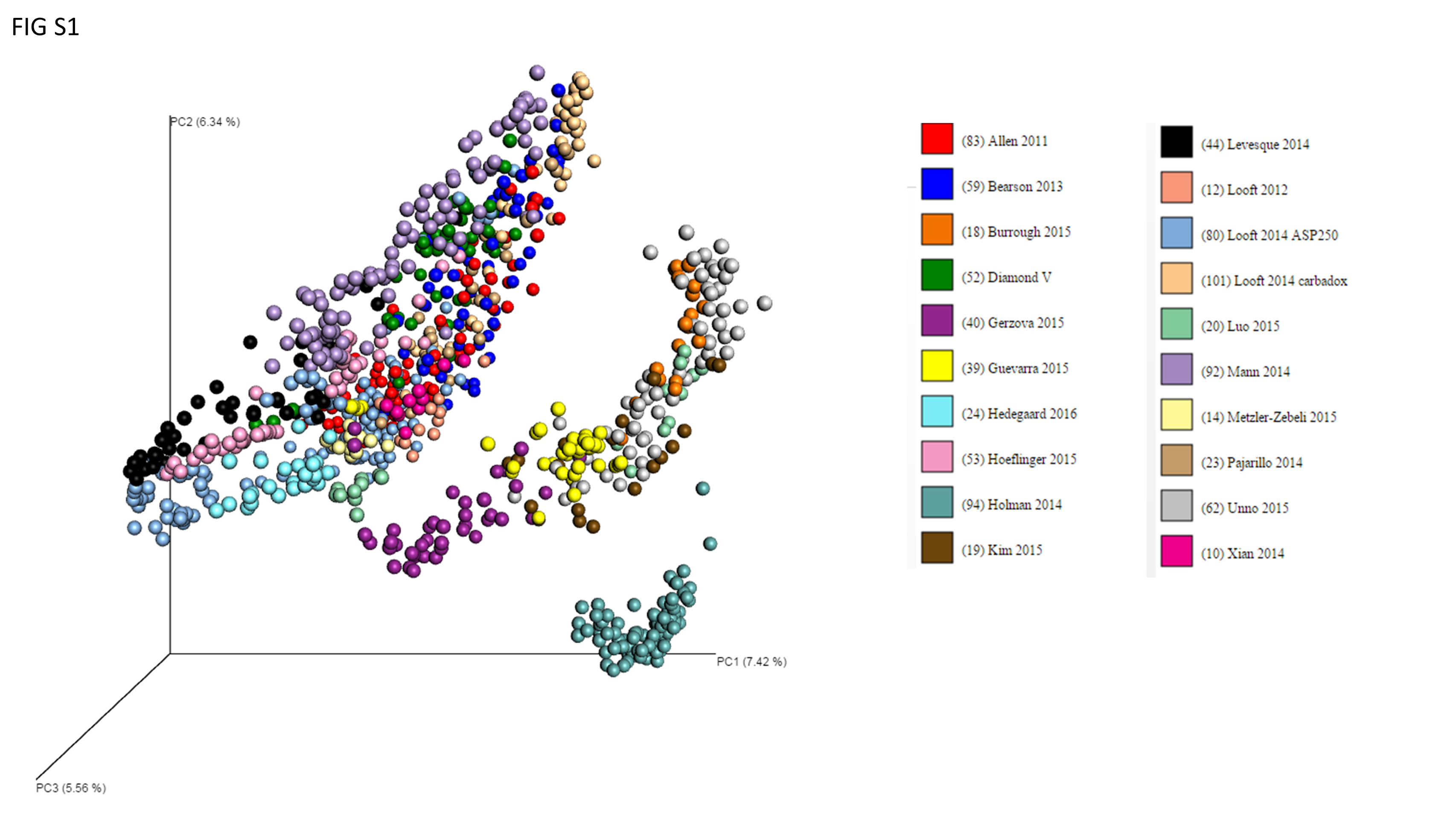

Supplement: FIG S1 [file sys003172103sf1.tif]

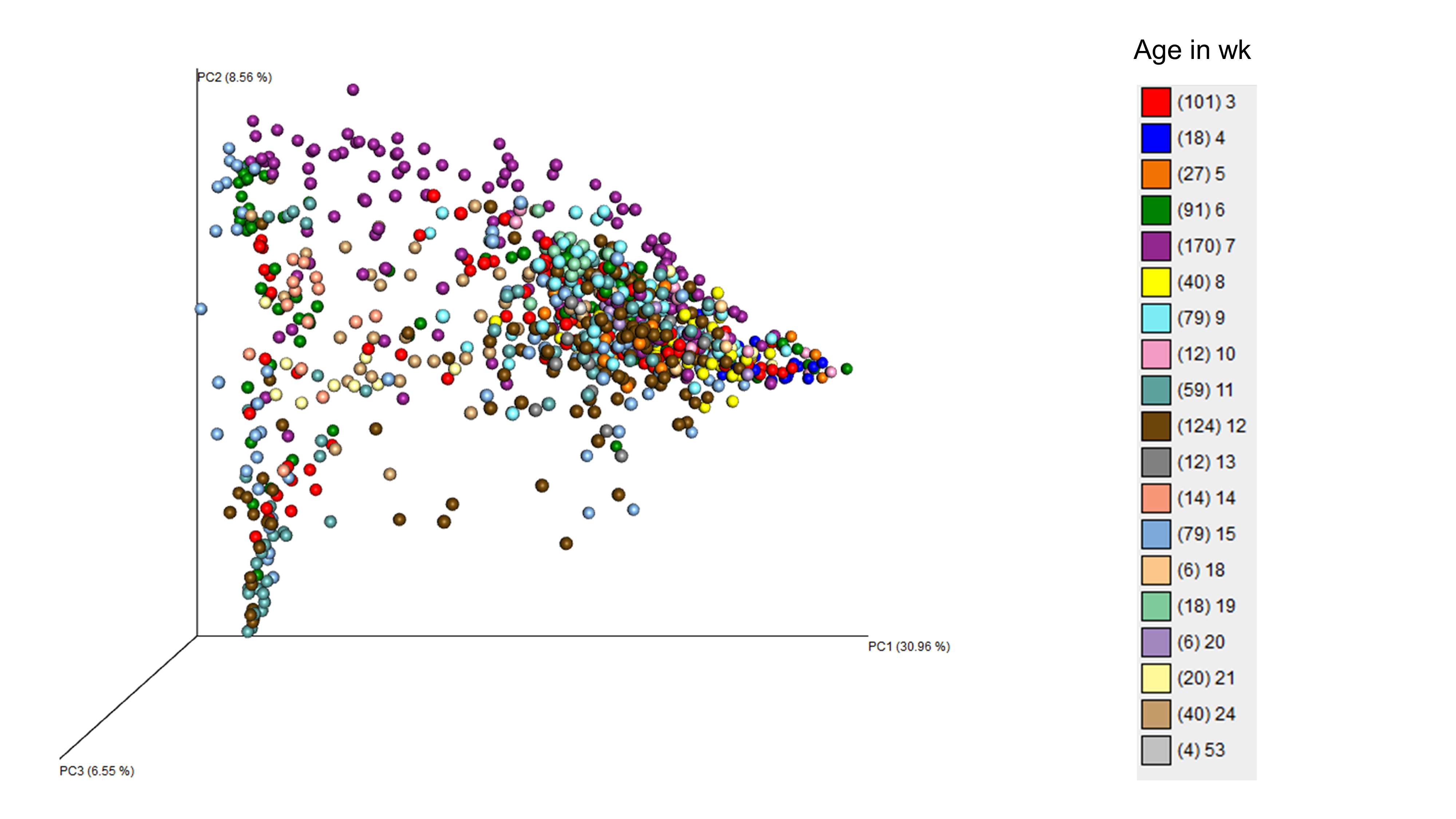

Supplement: FIG S2 [file sys003172103sf2.tif]

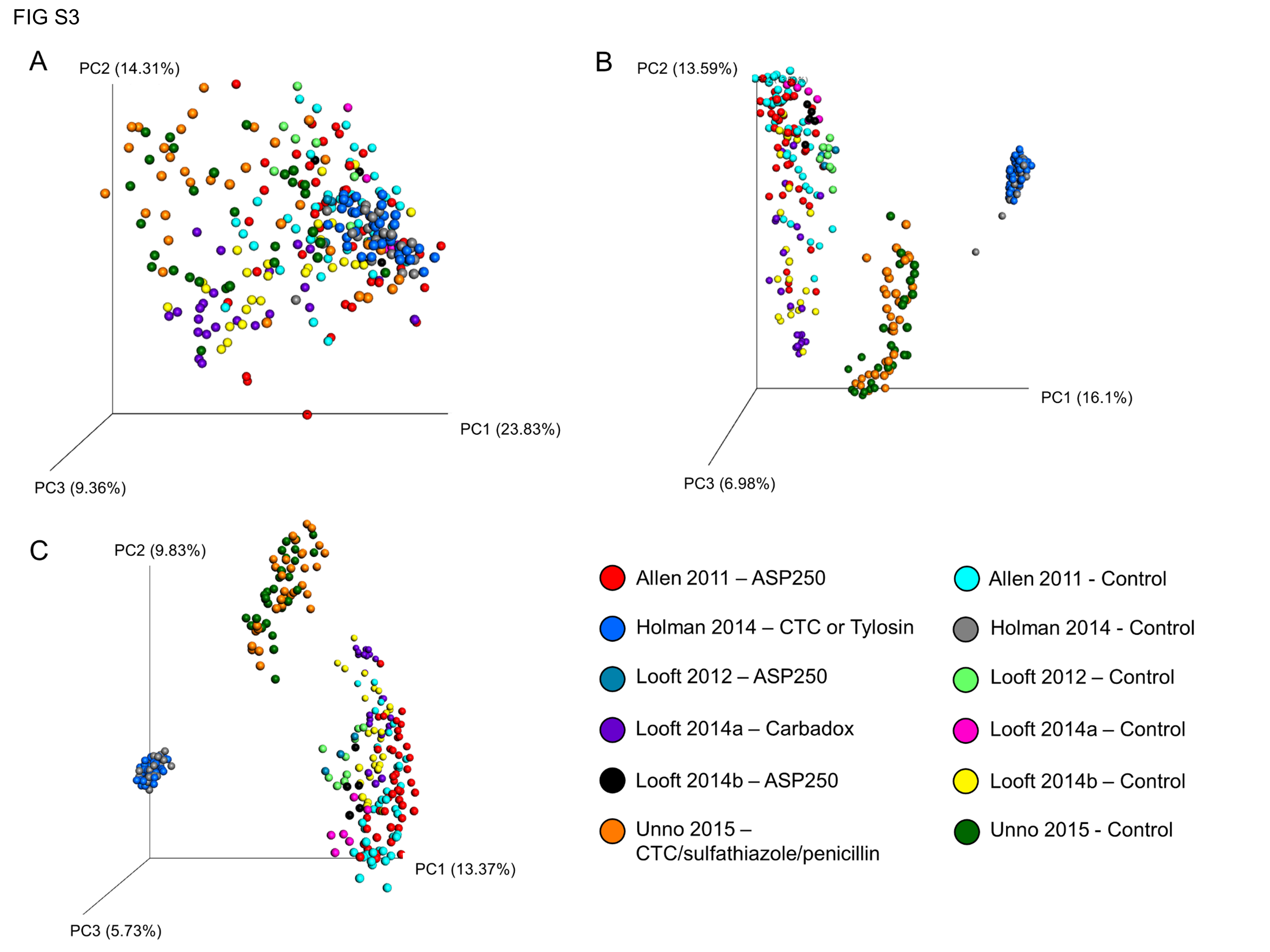

Supplement: FIG S3 [file sys003172103sf3.tif]
