# Supplementary material for: Meta-analysis To Define a Core Microbiota in the Swine Gut
Source: mSystems. 2017 May 23;2(3):e00004-17. doi: 10.1128/mSystems.00004-17 (PMC5443231; doi:10.1128/mSystems.00004-17)
Supplement: TABLE S2 [file sys003172103st5.pdf]

TABLE S2. The percentage of fecal samples grouped by specific 16S rRNA gene hypervariable region sequenced that had at least one 16S rRNA gene sequence from each of the individual genera identified. Genera are listed in descending order by overall relative abundance.
The number of fecal samples for each specific 16S rRNA gene hypervariable region sequenced is indicated in parentheses.
The overall relative abundance among all fecal samples is listed as the percent mean ± standard deviation.

Genus	V1 to V3 (n = 270)	V3 (n = 12)	V3 to V4 (n = 14)	V4 (n = 214)	Overall percent relative abundance
Prevotella	100.0	100.0	92.9	99.5	2.331 ± 2.216
Treponema	96.3	100.0	0.0	96.7	2.185 ± 1.945
Succinivibrio	95.9	100.0	78.6	93.9	1.73 ± 2.026
Clostridium	98.5	100.0	100.0	100.0	1.164 ± 0.892
Lactobacillus	92.6	66.7	100.0	97.7	1.05 ± 2.071
RC9 gut group	100.0	100.0	100.0	97.7	1.011 ± 0.984
Blautia	100.0	100.0	92.9	97.7	0.907 ± 1.595
Alloprevotella	100.0	100.0	100.0	98.1	0.883 ± 0.972
Ruminococcus	100.0	100.0	100.0	97.7	0.854 ± 1.91
Faecalibacterium	95.2	100.0	92.9	97.2	0.792 ± 1.778
Phascolarctobacterium	99.6	100.0	92.9	94.9	0.719 ± 0.826
Roseburia	100.0	100.0	92.9	95.8	0.699 ± 2.099
Parabacteroides	99.3	100.0	64.3	95.3	0.64 ± 1.544
Megasphaera	74.8	8.3	92.9	93.9	0.582 ± 0.774
Streptococcus	87.8	100.0	100.0	75.7	0.524 ± 0.538
Pseudobutyrivibrio	98.5	100.0	92.9	97.2	0.51 ± 0.566
Bacteroides	83.0	75.0	57.1	92.1	0.443 ± 2.543
Catenibacterium	83.0	41.7	0.0	59.8	0.437 ± 1.012
Anaerovibrio	93.3	100.0	78.6	95.8	0.429 ± 0.544
Subdoligranulum	99.3	91.7	85.7	96.3	0.428 ± 1.27
Coprococcus	99.6	100.0	92.9	94.9	0.347 ± 0.957
Escherichia-Shigella	48.5	100.0	71.4	79.4	0.325 ± 0.567
Spirochaeta	91.5	100.0	0.0	12.1	0.298 ± 1.385
Oscillibacter	98.1	100.0	92.9	95.3	0.291 ± 0.857
Campylobacter	60.0	58.3	71.4	86.9	0.278 ± 0.478
Acetitomaculum	84.8	66.7	21.4	65.4	0.252 ± 0.396
Sarcina	94.8	100.0	71.4	92.5	0.252 ± 0.427
Selenomonas	68.9	75.0	92.9	86.9	0.234 ± 0.273
Oribacterium	90.0	91.7	57.1	67.3	0.225 ± 0.982
dgA-11 gut group	67.4	100.0	71.4	90.2	0.196 ± 0.396
Anaerotruncus	95.9	83.3	92.9	93.0	0.188 ± 0.352
Solobacterium	96.3	66.7	0.0	88.3	0.18 ± 0.382
Marvinbryantia	96.7	83.3	78.6	92.5	0.177 ± 0.299
Dialister	57.0	100.0	78.6	76.2	0.164 ± 0.437
Desulfovibrio	51.9	50.0	78.6	91.6	0.158 ± 3.404
Oscillospira	64.8	83.3	85.7	96.7	0.158 ± 0.965
Fibrobacter	50.7	16.7	42.9	57.9	0.145 ± 0.181
Anaerostipes	84.8	91.7	7.1	75.2	0.141 ± 0.341
Desulfitibacter	77.8	41.7	7.1	0.5	0.124 ± 0.481
Psychrobacter	0.0	0.0	50.0	3.3	0.114 ± 0.376
Candidatus Saccharimonas	60.4	41.7	71.4	0.9	0.112 ± 0.353
Sutterella	88.1	25.0	0.0	94.9	0.111 ± 0.616
p-1088-a5 gut group	47.4	100.0	0.0	60.3	0.11 ± 0.691
Butyricimonas	3.3	0.0	0.0	51.4	0.109 ± 0.204
Elusimicrobium	2.6	25.0	7.1	61.2	0.108 ± 0.201
Turicibacter	51.1	83.3	100.0	52.3	0.106 ± 0.349
Asteroleplasma	28.9	8.3	0.0	9.3	0.084 ± 0.185
Anaeroplasma	32.6	8.3	7.1	50.9	0.082 ± 0.463
Collinsella	85.2	33.3	92.9	93.0	0.076 ± 0.233
Incertae Sedis	84.1	58.3	7.1	76.6	0.072 ± 0.251
Mitsuokella	55.6	16.7	64.3	51.4	0.071 ± 0.106
Mogibacterium	64.1	16.7	100.0	84.1	0.07 ± 0.271
Pseudomonas	60.7	83.3	78.6	18.7	0.069 ± 0.26
Alistipes	18.9	25.0	7.1	66.4	0.067 ± 0.236
Acidaminococcus	40.0	0.0	57.1	20.6	0.064 ± 1.185
Dorea	63.3	83.3	42.9	91.1	0.059 ± 0.942
Thalassospira	31.1	75.0	21.4	52.8	0.059 ± 0.27
Bifidobacterium	0.4	0.0	92.9	74.3	0.057 ± 1.009
Akkermansia	20.4	66.7	0.0	45.8	0.055 ± 0.099
Haliangium	0.0	0.0	0.0	2.8	0.051 ± 0.157
Nannocystis	0.0	0.0	0.0	3.7	0.05 ± 0.993
Ruminobacter	15.9	41.7	0.0	55.6	0.05 ± 0.261
Acinetobacter	4.4	0.0	42.9	40.2	0.048 ± 0.178
Lachnospira	43.3	41.7	57.1	85.5	0.046 ± 0.105
Enterorhabdus	58.9	0.0	57.1	64.0	0.043 ± 0.251
Hydrogenophilus	0.0	0.0	92.9	0.0	0.042 ± 0.086
Cloacibacillus	5.6	0.0	7.1	47.7	0.039 ± 0.173
Arthrobacter	46.3	41.7	14.3	8.9	0.035 ± 0.087
Butyrivibrio	44.4	16.7	57.1	83.6	0.03 ± 0.139
Enterococcus	7.4	0.0	28.6	55.1	0.03 ± 0.049
Helicobacter	47.8	8.3	7.1	74.3	0.029 ± 0.042
Fusobacterium	44.4	41.7	0.0	46.3	0.028 ± 0.073
Victivallis	35.2	0.0	0.0	63.6	0.028 ± 0.475
Bilophila	0.4	0.0	0.0	51.4	0.026 ± 0.085
Peptococcus	42.2	33.3	57.1	77.1	0.025 ± 0.283
Pyramidobacter	46.3	50.0	0.0	59.8	0.024 ± 0.052
Anaerosporobacter	27.0	83.3	35.7	68.7	0.023 ± 0.041

Thermomonas	0.0	0.0	7.1	2.3	0.021 ± 0.209
Enterobacter	29.3	25.0	7.1	52.3	0.016 ± 0.044
Reyranella	0.0	0.0	0.0	2.8	0.015 ± 0.089
Oxalobacter	54.4	41.7	14.3	42.5	0.015 ± 0.202
Shuttleworthia	45.2	8.3	35.7	65.4	0.014 ± 0.211
Proteiniphilum	0.0	0.0	35.7	56.1	0.014 ± 0.229
Intestinimonas	4.1	8.3	14.3	73.4	0.014 ± 0.079
Syntrophococcus	12.2	25.0	50.0	18.2	0.014 ± 0.042
Planctomyces	0.0	0.0	0.0	3.7	0.013 ± 0.052
Gemmata	0.0	0.0	0.0	2.8	0.013 ± 0.063
Devosia	0.0	0.0	7.1	3.7	0.012 ± 0.057
Rhodococcus	25.9	25.0	14.3	8.9	0.012 ± 0.147
Flavonifractor	34.8	16.7	57.1	33.6	0.011 ± 0.042
Cellulosilyticum	12.6	58.3	14.3	42.1	0.011 ± 0.069
Leeia	7.0	16.7	7.1	43.5	0.01 ± 0.09
Planomicrobium	4.1	0.0	14.3	50.5	0.009 ± 0.096
Ferruginibacter	0.0	0.0	0.0	2.8	0.009 ± 0.047
Caldicoprobacter	20.7	91.7	0.0	29.9	0.009 ± 0.203
Gardnerella	13.3	0.0	0.0	2.8	0.009 ± 0.196
Allisonella	17.0	0.0	35.7	18.2	0.009 ± 0.025
Actinobacillus	7.0	0.0	0.0	33.6	0.009 ± 0.03
Paludibacter	20.4	8.3	0.0	11.2	0.008 ± 0.027
Geobacter	0.0	0.0	0.0	0.5	0.008 ± 0.081
Geothrix	0.0	0.0	0.0	0.9	0.008 ± 0.095
Papillibacter	35.9	8.3	0.0	12.6	0.008 ± 0.101
Paraprevotella	1.1	0.0	0.0	41.6	0.008 ± 0.162
Quinella	5.6	75.0	7.1	40.2	0.007 ± 0.03
Ignavigranum	0.0	0.0	35.7	12.1	0.007 ± 0.138
Megamonas	16.3	0.0	0.0	10.3	0.007 ± 0.131
Acidovorax	0.0	0.0	7.1	5.1	0.007 ± 0.157
Perlucidibaca	0.0	0.0	0.0	0.9	0.007 ± 0.104
Massilia	29.6	33.3	0.0	7.9	0.007 ± 0.081
Prosthecomicrobium	0.0	0.0	0.0	1.4	0.007 ± 0.09
Polynucleobacter	0.0	0.0	0.0	3.7	0.007 ± 0.031
Desulfocapsa	0.0	0.0	0.0	0.9	0.007 ± 0.023
Opitutus	0.0	0.0	0.0	2.8	0.007 ± 0.033
Variovorax	0.0	0.0	14.3	5.6	0.006 ± 0.061
Sulfuritalea	0.0	0.0	0.0	1.9	0.006 ± 0.139
Parasutterella	6.3	0.0	28.6	19.6	0.006 ± 0.026
Hydrogenoanaerobacterium	2.2	8.3	0.0	41.6	0.006 ± 0.124
Odoribacter	0.0	0.0	0.0	37.9	0.006 ± 0.066
Trichococcus	0.4	0.0	42.9	15.9	0.006 ± 0.097
Exiguobacterium	0.0	0.0	0.0	5.6	0.005 ± 0.117
Corynebacterium	4.1	0.0	64.3	47.2	0.005 ± 0.12
Halomonas	0.0	0.0	0.0	1.4	0.005 ± 0.116
Finegoldia	0.4	0.0	0.0	8.9	0.005 ± 0.07
Ignatzschineria	0.0	0.0	21.4	7.5	0.005 ± 0.107
Caryophanon	0.0	0.0	0.0	6.1	0.005 ± 0.039
Cetobacterium	1.9	0.0	0.0	1.4	0.005 ± 0.028
Vibrio	0.0	0.0	0.0	1.4	0.005 ± 0.022
Woodsholea	0.0	0.0	0.0	1.9	0.005 ± 0.035
Rickettsiella	0.0	0.0	0.0	0.9	0.005 ± 0.073
Actinomyces	0.4	0.0	0.0	34.6	0.005 ± 0.055
Weissella	10.4	25.0	14.3	6.1	0.004 ± 0.012
Bacillus	4.1	0.0	7.1	34.6	0.004 ± 0.041
Sharpea	9.6	0.0	0.0	7.5	0.004 ± 0.069
Thiobacillus	0.0	0.0	0.0	1.4	0.004 ± 0.019
Nitrospira	0.0	0.0	0.0	2.8	0.004 ± 0.017
Christensenella	6.7	25.0	28.6	41.6	0.004 ± 0.027
Novosphingobium	0.0	0.0	0.0	2.8	0.004 ± 0.041
Limnohabitans	0.0	0.0	0.0	7.0	0.004 ± 0.01
Candidatus Cloacamonas	14.4	0.0	0.0	0.0	0.004 ± 0.073
Butyricicoccus	16.7	58.3	0.0	6.1	0.004 ± 0.04
Fastidiosipila	11.1	33.3	14.3	6.5	0.004 ± 0.019
Bradyrhizobium	0.4	0.0	0.0	3.3	0.004 ± 0.046
Moryella	10.7	16.7	0.0	42.5	0.004 ± 0.041
Chryseobacterium	0.4	8.3	14.3	2.8	0.004 ± 0.013
Porphyromonas	0.0	0.0	14.3	7.0	0.004 ± 0.023
Synergistes	2.2	0.0	0.0	35.0	0.003 ± 0.026
Zoogloea	0.0	0.0	0.0	2.3	0.003 ± 0.066
Jannaschia	0.0	0.0	0.0	2.3	0.003 ± 0.078
Mucispirillum	0.4	0.0	7.1	46.7	0.003 ± 0.033
Sphingomonas	6.7	16.7	0.0	3.7	0.003 ± 0.038
Atopobium	1.5	0.0	35.7	26.2	0.003 ± 0.035
Altererythrobacter	0.0	0.0	7.1	2.3	0.003 ± 0.047
Ferritrophicum	0.0	0.0	0.0	0.5	0.003 ± 0.02
Kluyvera	0.7	0.0	0.0	21.0	0.003 ± 0.054
Bdellovibrio	0.0	0.0	0.0	2.3	0.003 ± 0.032
Comamonas	0.0	0.0	7.1	7.5	0.003 ± 0.029
Hirschia	0.0	0.0	0.0	2.3	0.003 ± 0.034
Flavobacterium	0.4	0.0	14.3	18.2	0.003 ± 0.023
Phyllobacterium	0.0	8.3	7.1	9.3	0.003 ± 0.017
Phenylobacterium	0.0	0.0	0.0	3.7	0.003 ± 0.026
Kurthia	0.0	0.0	7.1	15.0	0.003 ± 0.023

Nitratireductor	0.0	0.0	0.0	2.8	0.003 ± 0.026
Lysinibacillus	0.0	0.0	7.1	15.4	0.003 ± 0.011
Anaerobiospirillum	8.1	58.3	0.0	9.8	0.003 ± 0.015
Lachnoanaerobaculum	0.4	83.3	0.0	0.0	0.003 ± 0.031
Haemophilus	0.4	0.0	14.3	18.2	0.003 ± 0.04
Schwartzia	7.8	25.0	0.0	5.1	0.003 ± 0.035
Smithella	0.0	0.0	0.0	32.2	0.003 ± 0.008
Veillonella	5.6	0.0	0.0	21.0	0.002 ± 0.016
Mycobacterium	0.0	0.0	0.0	2.3	0.002 ± 0.01
Aeromonas	0.4	0.0	14.3	5.6	0.002 ± 0.031
Solitalea	0.0	0.0	0.0	2.3	0.002 ± 0.049
Anaerovorax	0.4	0.0	0.0	39.3	0.002 ± 0.029
Peptostreptococcus	1.9	0.0	21.4	23.8	0.002 ± 0.036
Rothia	3.0	0.0	7.1	23.8	0.002 ± 0.031
Zymomonas	0.0	0.0	0.0	1.9	0.002 ± 0.036
Caldibacillus	0.0	0.0	28.6	0.0	0.002 ± 0.033
Candidatus Liberibacter	0.0	0.0	0.0	1.9	0.002 ± 0.022
Caulobacter	0.0	0.0	0.0	2.3	0.002 ± 0.01
Emticicia	0.0	0.0	0.0	1.9	0.002 ± 0.02
Sphingobium	0.0	0.0	7.1	2.3	0.002 ± 0.033
Acetivibrio ethanolgignens	0.7	0.0	0.0	4.7	0.002 ± 0.031
Mobiluncus	0.0	0.0	0.0	8.9	0.002 ± 0.044
Lactococcus	11.9	0.0	0.0	3.7	0.002 ± 0.013
Staphylococcus	2.6	0.0	35.7	15.4	0.002 ± 0.033
SM1A02	0.0	0.0	0.0	1.4	0.002 ± 0.01
Rhodobacter	0.0	0.0	0.0	3.7	0.002 ± 0.016
Desulfosporosinus	0.0	0.0	0.0	0.9	0.002 ± 0.032
Epulopiscium	7.4	0.0	0.0	6.1	0.002 ± 0.016
Mitsuaria	0.0	0.0	0.0	0.9	0.002 ± 0.016
Sedimentibacter	7.0	0.0	7.1	7.9	0.002 ± 0.03
Pasteurella	1.9	0.0	0.0	4.2	0.002 ± 0.007
Defluviimonas	0.4	0.0	0.0	2.3	0.002 ± 0.016
Simplicispira	0.0	0.0	0.0	4.7	0.002 ± 0.033
Chlorochromatium	0.0	0.0	0.0	15.4	0.002 ± 0.02
OM43 clade	0.0	0.0	7.1	1.4	0.002 ± 0.013
Slackia	13.7	8.3	14.3	4.2	0.001 ± 0.028
Leptothrix	0.0	0.0	0.0	3.7	0.001 ± 0.031
Deinococcus	0.0	0.0	0.0	1.4	0.001 ± 0.007
Achromobacter	0.0	0.0	0.0	6.1	0.001 ± 0.018
Anaerococcus	0.0	0.0	0.0	8.9	0.001 ± 0.017
Candidatus Planktophila	0.0	0.0	0.0	1.4	0.001 ± 0.009
Anaeromyxobacter	0.0	0.0	0.0	0.9	0.001 ± 0.017
Serratia	0.0	0.0	0.0	20.6	0.001 ± 0.027
Bryobacter	0.0	0.0	0.0	1.9	0.001 ± 0.031
Proteiniclasticum	0.0	0.0	7.1	11.7	0.001 ± 0.027
Facklamia	0.0	0.0	21.4	9.3	0.001 ± 0.011
Aquabacterium	0.0	0.0	0.0	2.8	0.001 ± 0.008
Desulfobulbus	0.0	0.0	7.1	0.5	0.001 ± 0.015
Sideroxydans	0.0	0.0	0.0	0.9	0.001 ± 0.024
C1-B045	0.0	0.0	0.0	0.9	0.001 ± 0.019
Peptoniphilus	0.7	0.0	14.3	12.6	0.001 ± 0.007
Petrimonas	3.7	8.3	14.3	4.2	0.001 ± 0.009
Tissierella	0.0	0.0	21.4	8.4	0.001 ± 0.017
Blastocatella	0.0	0.0	0.0	1.4	0.001 ± 0.011
Hyphomicrobium	0.0	0.0	0.0	1.9	0.001 ± 0.013
Howardella	5.2	0.0	21.4	15.9	0.001 ± 0.006
Eremococcus	0.0	0.0	7.1	11.2	0.001 ± 0.024
Roseiflexus	0.0	0.0	0.0	1.4	0.001 ± 0.017
Sphingopyxis	0.0	0.0	0.0	4.2	0.001 ± 0.025
Nordella	0.0	0.0	0.0	2.3	0.001 ± 0.018
Erysipelothrix	3.3	8.3	0.0	8.9	0.001 ± 0.015
Malikia	0.0	0.0	0.0	1.9	0.001 ± 0.018
Rhizomicrobium	0.0	0.0	0.0	2.3	0.001 ± 0.005
Pelobacter	0.0	0.0	0.0	0.5	0.001 ± 0.012
Sporosarcina	0.0	0.0	7.1	2.8	0.001 ± 0.007
Flavihumibacter	0.0	0.0	0.0	1.4	0.001 ± 0.016
Jeotgalicoccus	0.0	0.0	7.1	6.5	0.001 ± 0.012
Sporobacter	0.0	0.0	0.0	15.9	0.001 ± 0.011
Vogesella	0.0	0.0	0.0	2.3	0.001 ± 0.015
Aerococcus	0.7	0.0	7.1	11.2	0.001 ± 0.004
Gemmatimonas	0.0	0.0	0.0	1.9	0.001 ± 0.012
Arcobacter	0.4	0.0	7.1	5.6	0.001 ± 0.011
Sorangium	0.0	0.0	0.0	2.8	0.001 ± 0.015
Nitrosomonas	0.0	0.0	0.0	2.3	0.001 ± 0.005
Allobaculum	7.4	0.0	0.0	11.2	0.001 ± 0.007
Candidatus Alysiosphaera	0.0	0.0	0.0	3.3	0.001 ± 0.013
Piscinibacter	0.0	0.0	0.0	2.3	0.001 ± 0.019
Candidatus Microthrix	0.0	0.0	0.0	1.4	0.001 ± 0.01
Anaerofilum	5.6	16.7	0.0	9.8	0.001 ± 0.018
Paenalcaligenes	0.0	0.0	7.1	6.5	0.001 ± 0.018
Marinospirillum	0.0	0.0	0.0	2.3	0.001 ± 0.013
Janthinobacterium	0.0	0.0	0.0	0.5	0.001 ± 0.016
Citrobacter	4.8	0.0	0.0	0.9	0.001 ± 0.008
Desulfotalea	0.0	0.0	0.0	0.5	0.001 ± 0.009

Aquicella	0.0	0.0	0.0	0.5	0.001 ± 0.005
Filimonas	0.0	0.0	0.0	1.4	0.001 ± 0.01
Carnobacterium	0.0	0.0	21.4	0.5	0.001 ± 0.008
Syntrophomonas	4.4	0.0	0.0	0.5	0.001 ± 0.008
Thioclava	0.0	0.0	7.1	1.9	0.001 ± 0.014
Stenotrophomonas	0.4	0.0	21.4	7.9	0.001 ± 0.008
Pirellula	0.0	0.0	0.0	1.9	0.001 ± 0.003
Sediminibacterium	0.0	0.0	0.0	3.7	0.001 ± 0.007
Legionella	0.0	0.0	0.0	2.3	0.001 ± 0.005
Patulibacter	0.0	0.0	0.0	0.9	0.001 ± 0.004
Rhodovulum	0.0	0.0	0.0	2.8	0.001 ± 0.013
Anaerofustis	3.3	0.0	0.0	16.4	0.001 ± 0.006
Pantoea	1.9	0.0	0.0	5.6	0.001 ± 0.005
Pseudoramibacter	1.5	0.0	0.0	9.3	0.001 ± 0.006
Noviherbaspirillum	0.0	0.0	7.1	6.5	0.001 ± 0.013
Desulfatiferula	0.0	0.0	0.0	0.9	0.001 ± 0.004
Trueperella	0.0	0.0	0.0	9.3	0.001 ± 0.013
Olsenella	4.4	0.0	14.3	0.5	0.001 ± 0.01
Candidatus Hepatincola	3.3	0.0	0.0	1.9	0.001 ± 0.004
Azoarcus	0.0	0.0	0.0	0.5	0.001 ± 0.01
Leuconostoc	0.4	0.0	0.0	9.3	0.001 ± 0.007
Macellibacteroides	0.0	0.0	0.0	0.5	0.001 ± 0.009
Methylotenera	0.0	0.0	0.0	1.4	0.001 ± 0.011
SP3-e08	3.0	58.3	0.0	0.5	0.001 ± 0.008
Terrimonas	0.0	0.0	0.0	1.4	0 ± 0.005
Roseomonas	0.0	0.0	7.1	1.9	0 ± 0.011
Proteus	0.0	0.0	21.4	2.8	0 ± 0.006
Sulfuricurvum	0.0	0.0	0.0	0.9	0 ± 0.005
Nevskia	0.0	0.0	0.0	0.9	0 ± 0.009
Gemella	0.0	0.0	7.1	5.6	0 ± 0.005
Sanguibacter	0.0	0.0	7.1	0.5	0 ± 0.01
Polaromonas	0.0	0.0	7.1	1.9	0 ± 0.01
Synechococcus	0.0	0.0	0.0	6.1	0 ± 0.006
Alpinimonas	0.0	0.0	0.0	1.4	0 ± 0.006
Herbaspirillum	0.0	0.0	0.0	6.1	0 ± 0.005
Methyloversatilis	0.4	0.0	0.0	0.5	0 ± 0.01
Ralstonia	0.4	0.0	0.0	0.5	0 ± 0.006
Rickettsia	0.0	0.0	0.0	1.4	0 ± 0.004
Pedomicrobium	0.0	0.0	0.0	2.3	0 ± 0.01
Dokdonella	0.0	0.0	0.0	2.8	0 ± 0.004
Thermincola	0.0	0.0	0.0	0.5	0 ± 0.004
Lysinimonas	0.0	0.0	0.0	1.9	0 ± 0.007
Clostridiales bacterium 20-2a	0.4	0.0	0.0	4.7	0 ± 0.005
Ornatilinea	0.0	0.0	0.0	0.5	0 ± 0.004
Vagococcus	0.0	0.0	0.0	6.5	0 ± 0.008
Atopostipes	0.0	0.0	21.4	2.8	0 ± 0.006
Stella	0.0	0.0	0.0	0.9	0 ± 0.007
Luteimonas	0.0	0.0	0.0	4.7	0 ± 0.007
Cronobacter	0.7	0.0	0.0	4.7	0 ± 0.004
Cellvibrio	0.0	0.0	0.0	1.4	0 ± 0.005
Methylophilus	0.0	0.0	0.0	1.4	0 ± 0.005
Undibacterium	0.0	8.3	0.0	1.4	0 ± 0.006
Hydrotalea	0.0	0.0	0.0	1.4	0 ± 0.008
Actinobaculum	0.0	0.0	0.0	4.7	0 ± 0.003
Nitrobacter	0.0	0.0	0.0	1.4	0 ± 0.002
Candidatus Captivus	0.0	0.0	0.0	1.9	0 ± 0.005
Luteolibacter	0.0	0.0	0.0	1.9	0 ± 0.007
Shewanella	0.0	0.0	0.0	1.4	0 ± 0.004
Murdochiella	0.0	0.0	0.0	7.9	0 ± 0.005
Streptomyces	0.0	0.0	0.0	9.8	0 ± 0.005
Ottowia	0.0	0.0	0.0	1.9	0 ± 0.006
GKS98 freshwater group	0.0	0.0	0.0	0.9	0 ± 0.002
Rhodobium	0.0	0.0	0.0	1.9	0 ± 0.006
Arenimonas	0.0	0.0	0.0	1.9	0 ± 0.007
Runella	0.0	0.0	0.0	1.4	0 ± 0.003
Enhydrobacter	0.0	0.0	7.1	1.4	0 ± 0.004
Aggregatibacter	0.0	0.0	0.0	7.0	0 ± 0.006
Bosea	0.0	0.0	0.0	1.4	0 ± 0.004
Methylobacter	0.0	0.0	0.0	0.5	0 ± 0.004
Paenibacillus	2.6	0.0	7.1	0.9	0 ± 0.005
Acidiferrobacter	0.0	0.0	0.0	1.4	0 ± 0.004
Defluviicoccus	0.0	0.0	0.0	0.9	0 ± 0.007
Azospira	0.0	0.0	0.0	1.9	0 ± 0.007
Moraxella	0.7	0.0	0.0	3.7	0 ± 0.003
Cytophaga	0.0	0.0	0.0	1.4	0 ± 0.005
Prochlorococcus	0.0	0.0	0.0	3.3	0 ± 0.007
Erythrobacter	0.0	0.0	0.0	0.5	0 ± 0.005
Byssovorax	0.0	0.0	0.0	0.5	0 ± 0.002
Solibacillus	0.0	0.0	0.0	5.6	0 ± 0.004
Virgibacillus	0.0	0.0	0.0	4.2	0 ± 0.004
Desulfatirhabdium	0.0	0.0	0.0	0.5	0 ± 0.003
Chthoniobacter	0.0	0.0	0.0	1.4	0 ± 0.006
Arcanobacterium	0.0	0.0	0.0	5.6	0 ± 0.003
Brevundimonas	0.0	0.0	0.0	3.3	0 ± 0.004

Acidocella	0.0	0.0	0.0	0.9	0 ± 0.002
Brachymonas	0.0	0.0	0.0	3.3	0 ± 0.006
Bibersteinia	0.4	0.0	0.0	0.0	0 ± 0.006
Succiniclasticum	0.4	0.0	0.0	4.7	0 ± 0.003
Kineosporia	0.0	0.0	0.0	1.4	0 ± 0.004
Eggerthella	0.4	0.0	0.0	5.6	0 ± 0.003
Tolumonas	0.0	0.0	0.0	0.5	0 ± 0.003
Longilinea	0.0	0.0	0.0	0.5	0 ± 0.006
Candidatus Competibacter	0.0	0.0	0.0	3.3	0 ± 0.006
LD28 freshwater group	0.0	0.0	0.0	1.9	0 ± 0.003
CL500-3	0.0	0.0	0.0	2.8	0 ± 0.002
Thauera	0.0	0.0	0.0	1.9	0 ± 0.002
Azospirillum	0.0	0.0	0.0	0.5	0 ± 0.002
Desulfopila	0.0	0.0	0.0	0.5	0 ± 0.004
Sandarakinorhabdus	0.0	0.0	0.0	1.9	0 ± 0.003
Gelria	0.0	0.0	0.0	6.1	0 ± 0.003
Catellicoccus	0.0	0.0	0.0	4.2	0 ± 0.005
Wautersiella	0.0	0.0	0.0	4.2	0 ± 0.005
Denitratisoma	0.0	0.0	0.0	0.9	0 ± 0.005
Pseudarcicella	0.0	0.0	0.0	1.4	0 ± 0.003
Lacticigenium	0.0	0.0	7.1	1.9	0 ± 0.003
Desulfuromonas	0.0	0.0	0.0	0.5	0 ± 0.004
Desulfotomaculum	0.0	0.0	0.0	0.5	0 ± 0.003
Deferrisoma	0.0	0.0	0.0	0.5	0 ± 0.002
Delftia	0.0	0.0	7.1	2.3	0 ± 0.002
Inquilinus	0.0	0.0	0.0	1.4	0 ± 0.003
Salmonella	1.1	0.0	0.0	0.0	0 ± 0.005
Pediococcus	0.4	0.0	7.1	2.3	0 ± 0.004
Globicatella	0.0	0.0	7.1	4.7	0 ± 0.003
Helcococcus	0.0	0.0	0.0	4.2	0 ± 0.002
Albidiferax	0.0	0.0	0.0	1.4	0 ± 0.002
Iamia	0.0	0.0	0.0	0.5	0 ± 0.002
12up	0.0	0.0	0.0	0.9	0 ± 0.001
Pseudoflavonifractor	0.7	0.0	0.0	0.5	0 ± 0.003
Raoultella	0.0	0.0	0.0	4.2	0 ± 0.003
Isosphaera	0.0	0.0	0.0	1.9	0 ± 0.003
Nocardia	0.0	0.0	0.0	1.9	0 ± 0.003
Anaerobacillus	0.0	0.0	0.0	4.7	0 ± 0.002
Paucimonas	0.0	0.0	0.0	0.9	0 ± 0.003
Cellulomonas	0.0	0.0	0.0	1.9	0 ± 0.001
Neisseria	0.0	0.0	14.3	0.9	0 ± 0.003
Acholeplasma	0.0	0.0	7.1	0.9	0 ± 0.003
Leucobacter	0.0	0.0	0.0	4.7	0 ± 0.003
Candidatus Odyssella	0.0	0.0	0.0	0.9	0 ± 0.004
Eubacterium	2.2	0.0	0.0	2.8	0 ± 0.001
Roseobacter clade CHAB-I-5 lineage	0.0	0.0	0.0	0.9	0 ± 0.004
Aequorivita	0.0	0.0	0.0	0.9	0 ± 0.002
Candidatus Solibacter	0.0	0.0	0.0	0.9	0 ± 0.002
Pseudogulbenkiania	0.0	0.0	0.0	0.5	0 ± 0.002
Gallicola	0.0	0.0	0.0	4.2	0 ± 0.003
Candidatus Metachlamydia	0.0	0.0	0.0	0.5	0 ± 0.002
Macrococcus	0.4	0.0	14.3	0.5	0 ± 0.003
Candidatus Arthromitus	0.4	0.0	0.0	2.3	0 ± 0.002
Brevibacterium	0.0	0.0	14.3	1.4	0 ± 0.003
Coxiella	0.0	0.0	0.0	0.9	0 ± 0.003
Saccharofermentans	2.6	0.0	0.0	0.0	0 ± 0.003
Gordonia	0.0	0.0	7.1	0.5	0 ± 0.003
Curvibacter	0.0	0.0	0.0	1.4	0 ± 0.003
Tahibacter	0.0	0.0	0.0	0.9	0 ± 0.002
Zhihengliuella	0.0	0.0	0.0	0.9	0 ± 0.001
Pseudorhodoferax	0.0	0.0	0.0	0.9	0 ± 0.003
Sulfuricella	0.0	0.0	0.0	0.5	0 ± 0.002
Bacteriovorax	0.0	0.0	0.0	0.5	0 ± 0.002
Prosthecobacter	0.0	0.0	0.0	0.9	0 ± 0.002
Wohlfahrtiimonas	0.0	0.0	0.0	5.6	0 ± 0.003
Propionibacterium	0.0	0.0	7.1	0.0	0 ± 0.002
Nocardioides	0.0	0.0	7.1	1.4	0 ± 0.001
Afipia	0.0	0.0	0.0	1.4	0 ± 0.002
Cryobacterium	0.0	0.0	7.1	0.9	0 ± 0.003
Azohydromonas	0.0	0.0	0.0	0.5	0 ± 0.002
Ornithinibacter	0.0	0.0	0.0	0.9	0 ± 0.001
Parvimonas	0.7	0.0	0.0	2.3	0 ± 0.002
Hydrogenophaga	0.0	0.0	0.0	1.4	0 ± 0.001
Dechloromonas	0.0	0.0	0.0	0.5	0 ± 0.001
Alkalibacter	0.0	0.0	0.0	2.3	0 ± 0.002
Alloiococcus	0.0	0.0	0.0	3.3	0 ± 0.003
Alkanindiges	0.0	0.0	0.0	0.9	0 ± 0.001
Rhizobium	0.0	0.0	14.3	1.4	0 ± 0.002
Hespellia	0.0	0.0	0.0	3.7	0 ± 0.002
Oligella	0.0	0.0	0.0	2.3	0 ± 0.003
Hymenobacter	0.0	0.0	0.0	0.5	0 ± 0.003
Edwardsiella	0.0	0.0	0.0	2.8	0 ± 0.002
Marmoricola	0.0	0.0	0.0	0.9	0 ± 0.001
Fonticella	0.0	0.0	0.0	0.9	0 ± 0.002

Azovibrio	0.0	0.0	0.0	0.5	0 ± 0.002
Myroides	0.0	0.0	7.1	0.0	0 ± 0.002
Tepidiphilus	0.0	0.0	14.3	0.0	0 ± 0.003
Morganella	0.0	0.0	0.0	2.3	0 ± 0.003
Pusillimonas	0.0	0.0	0.0	1.9	0 ± 0.001
Methylobacterium	0.0	0.0	14.3	0.5	0 ± 0.003
Mycoplasma	0.7	0.0	0.0	0.5	0 ± 0.002
Leptolinea	0.0	0.0	0.0	0.5	0 ± 0.001
Desulfurivibrio	0.0	0.0	0.0	0.5	0 ± 0.001
Johnsonella	1.1	8.3	0.0	0.0	0 ± 0.002
Chitinibacter	0.0	0.0	0.0	0.5	0 ± 0.001
Candidatus Methylacidiphilum	0.0	0.0	0.0	0.9	0 ± 0.002
Sphingobacterium	0.0	0.0	7.1	1.4	0 ± 0.002
Fluviicola	0.0	0.0	0.0	1.9	0 ± 0.002
Agromyces	0.0	0.0	0.0	0.9	0 ± 0.002
Otariodibacter	0.7	0.0	0.0	1.9	0 ± 0.002
Armatimonas	0.0	0.0	0.0	0.9	0 ± 0.002
Roseovarius	0.0	0.0	7.1	0.0	0 ± 0.002
Syntrophobacter	0.0	0.0	0.0	0.5	0 ± 0.002
Gaiella	0.0	0.0	0.0	0.5	0 ± 0.001
Acetobacterium	0.0	0.0	0.0	0.5	0 ± 0.001
Candidatus Nitrotoga	0.0	0.0	0.0	0.5	0 ± 0.001
Desulfarculus	0.0	0.0	0.0	0.5	0 ± 0.002
Paracoccus	0.4	0.0	7.1	0.0	0 ± 0.001
Listeria	0.0	0.0	0.0	1.9	0 ± 0.002
Tabrizicola	0.0	0.0	0.0	1.4	0 ± 0.001
Adlercreutzia	0.0	0.0	0.0	2.8	0 ± 0.001
Klebsiella	0.0	8.3	0.0	0.5	0 ± 0.002
Halocella	1.1	0.0	0.0	0.0	0 ± 0.002
Sandaracinus	0.0	0.0	0.0	0.5	0 ± 0.002
Curtobacterium	0.0	0.0	0.0	0.9	0 ± 0.001
Ensifer	0.0	0.0	0.0	0.9	0 ± 0.001
Thermodesulfovibrio	0.0	0.0	7.1	0.0	0 ± 0.001
Dongia	0.0	0.0	0.0	0.5	0 ± 0.001
Alkanibacter	0.0	0.0	0.0	0.5	0 ± 0.001
Nitrosococcus	0.0	0.0	0.0	0.9	0 ± 0.002
Labrys	0.0	0.0	0.0	0.9	0 ± 0.002
Lampropedia	0.0	0.0	0.0	1.4	0 ± 0.001
Candidatus Accumulibacter	0.0	0.0	0.0	0.9	0 ± 0.002
Fusibacter	0.7	0.0	0.0	0.0	0 ± 0.002
Flavisolibacter	0.0	0.0	0.0	0.5	0 ± 0.001
Lautropia	0.0	0.0	0.0	0.5	0 ± 0.001
Sphingomicrobium	0.0	0.0	0.0	0.9	0 ± 0.001
Xanthobacter	0.0	0.0	0.0	0.5	0 ± 0.001
Succinatimonas	0.4	0.0	0.0	0.0	0 ± 0.001
Ochrobactrum	0.0	0.0	0.0	1.4	0 ± 0.001
Cupriavidus	0.0	0.0	0.0	0.9	0 ± 0.001
Nosocomiicoccus	0.0	0.0	0.0	1.9	0 ± 0.001
Alkaliphilus	0.0	0.0	0.0	1.9	0 ± 0.001
Plesiomonas	0.0	0.0	0.0	1.4	0 ± 0.001
Barnesiella	0.4	0.0	0.0	0.0	0 ± 0.001
Thermus	0.0	0.0	0.0	1.4	0 ± 0.001
Aquamicrobium	0.0	0.0	0.0	0.9	0 ± 0.001
Stomatobaculum	0.4	8.3	0.0	0.0	0 ± 0.001
Planococcus	0.0	0.0	0.0	1.9	0 ± 0.001
Tardiphaga	0.0	0.0	0.0	0.9	0 ± 0.001
Roseateles	0.0	0.0	0.0	0.5	0 ± 0.001
Sphingosinicella	0.0	0.0	0.0	0.5	0 ± 0.001
Candidatus Rhabdochlamydia	0.0	0.0	0.0	0.5	0 ± 0.001
Fodinicola	0.0	0.0	0.0	0.5	0 ± 0.001
Pseudokineococcus	0.0	0.0	0.0	0.5	0 ± 0.001
Neochlamydia	0.0	0.0	0.0	0.5	0 ± 0.001
Gallionella	0.0	0.0	0.0	0.5	0 ± 0.001
Ramlibacter	0.0	0.0	0.0	0.9	0 ± 0.001
MWH-Ta3	0.0	0.0	0.0	0.9	0 ± 0.001
Geobacillus	0.4	0.0	0.0	1.4	0 ± 0.001
Dietzia	0.0	0.0	7.1	0.9	0 ± 0.001
Acetatifactor	1.1	0.0	0.0	0.0	0 ± 0.001
GAL15	0.0	0.0	0.0	0.5	0 ± 0.001
Desulfitobacterium	0.0	0.0	0.0	0.5	0 ± 0.001
Microvirga	0.0	0.0	0.0	0.5	0 ± 0.001
Proteocatella	0.0	0.0	0.0	0.5	0 ± 0.001
Sporichthya	0.0	0.0	0.0	0.5	0 ± 0.001
Brumimicrobium	0.0	0.0	0.0	0.5	0 ± 0.001
Chitinivorax	0.0	0.0	0.0	0.5	0 ± 0.001
Microcystis	0.0	0.0	0.0	1.4	0 ± 0.001
Isoptericola	0.0	0.0	0.0	2.8	0 ± 0.001
Microbacterium	0.0	0.0	0.0	0.5	0 ± 0.001
Octadecabacter	0.0	0.0	0.0	0.5	0 ± 0.001
Parvibacter	0.4	0.0	0.0	0.0	0 ± 0.001
Yaniella	0.0	0.0	0.0	1.9	0 ± 0.001
Acetanaerobacterium	0.7	0.0	0.0	0.0	0 ± 0.001
Providencia	0.0	0.0	0.0	1.9	0 ± 0.001
Synechococcus sp. MA0607D	0.0	0.0	0.0	1.9	0 ± 0.001

Diplorickettsia	0.0	0.0	0.0	0.5	0 ± 0.001
Phocaeicola	0.0	0.0	0.0	1.4	0 ± 0.001
Pedobacter	0.0	0.0	7.1	0.5	0 ± 0.001
Tepidimicrobium	0.0	0.0	0.0	1.4	0 ± 0.001
Kocuria	0.0	0.0	0.0	0.9	0 ± 0.001
Rikenella	0.0	0.0	0.0	1.4	0 ± 0.001
Nesterenkonia	0.0	0.0	0.0	1.4	0 ± 0.001
Dysgonomonas	0.0	0.0	0.0	1.4	0 ± 0.001
Coprothermobacter	0.4	0.0	0.0	0.0	0 ± 0.001
Rathayibacter	0.0	0.0	0.0	0.5	0 ± 0.001
Rahnella	0.0	0.0	0.0	0.5	0 ± 0.001
Clavibacter	0.0	0.0	0.0	0.5	0 ± 0.001
Dyadobacter	0.0	0.0	0.0	0.5	0 ± 0.001
Rhodanobacter	0.0	0.0	0.0	0.5	0 ± 0.001
Xanthomonas	0.0	0.0	0.0	0.5	0 ± 0.001
Bauldia	0.0	0.0	0.0	0.5	0 ± 0.001
Phycicoccus	0.0	0.0	0.0	0.5	0 ± 0.001
Herbiconiux	0.0	0.0	0.0	0.5	0 ± 0.001
Galbibacter	0.0	0.0	0.0	0.5	0 ± 0.001
Soonwooa	0.0	0.0	0.0	0.5	0 ± 0.001
Kaistia	0.0	0.0	0.0	0.5	0 ± 0.001
Pseudolabrys	0.0	0.0	0.0	0.5	0 ± 0.001
Subtercola	0.0	0.0	0.0	0.5	0 ± 0.001
Singulisphaera	0.0	0.0	0.0	0.5	0 ± 0.001
Edaphobacter	0.0	0.0	0.0	0.5	0 ± 0.001
Chroococcidiopsis	0.0	0.0	0.0	0.5	0 ± 0.001
Actibacterium	0.0	0.0	0.0	0.5	0 ± 0.001
Methylocaldum	0.0	0.0	0.0	0.5	0 ± 0.001
Turneriella	0.0	0.0	0.0	0.5	0 ± 0.001
Alicycliphilus	0.0	0.0	0.0	0.5	0 ± 0.001
Pseudospirillum	0.0	0.0	0.0	0.5	0 ± 0.001
Lysobacter	0.0	0.0	0.0	0.5	0 ± 0.001
Propionivibrio	0.0	0.0	0.0	0.5	0 ± 0.001
Rhodopila	0.0	0.0	0.0	0.5	0 ± 0.001
Macromonas	0.0	0.0	0.0	0.5	0 ± 0.001
Tepidicella	0.0	0.0	0.0	0.5	0 ± 0.001
Ferribacterium	0.0	0.0	0.0	0.5	0 ± 0.001
Candidatus Entotheonella	0.0	0.0	0.0	0.5	0 ± 0.001
Thermosinus	0.0	0.0	7.1	0.0	0 ± 0.001
Tepidimonas	0.0	0.0	7.1	0.0	0 ± 0.001
Caldicellulosiruptor	0.0	0.0	7.1	0.0	0 ± 0.001
Shinella	0.0	0.0	7.1	0.0	0 ± 0.001
Pleomorphomonas	0.0	0.0	7.1	0.0	0 ± 0.001
Basfia	0.7	0.0	0.0	0.0	0 ± 0.001
Cellulosimicrobium	0.0	0.0	0.0	0.9	0 ± 0.001
Pelagibacterium	0.0	0.0	7.1	0.0	0 ± 0.001
Janibacter	0.0	0.0	7.1	0.0	0 ± 0.001
Pseudochrobactrum	0.0	0.0	7.1	0.0	0 ± 0.001
Citricoccus	0.0	0.0	7.1	0.0	0 ± 0.001
Gelidibacter	0.0	0.0	7.1	0.0	0 ± 0.001
Holdemania	0.0	0.0	0.0	0.9	0 ± 0
Alysiella	0.0	0.0	0.0	0.5	0 ± 0.001
Persicitalea	0.0	0.0	0.0	0.5	0 ± 0
Oceanobacter	0.0	0.0	0.0	0.5	0 ± 0
Trabulsiella	0.4	0.0	0.0	0.0	0 ± 0
Pseudoclavibacter	0.0	0.0	0.0	0.9	0 ± 0
Nocardiopsis	0.0	0.0	0.0	0.5	0 ± 0
Leptotrichia	0.0	0.0	0.0	0.9	0 ± 0
Amphibacillus	0.0	0.0	0.0	0.9	0 ± 0
Robinsoniella	0.4	0.0	0.0	0.5	0 ± 0
Chlamydia	0.0	0.0	0.0	0.9	0 ± 0
Enteractinococcus	0.0	0.0	0.0	0.9	0 ± 0
Pseudoxanthomonas	0.0	0.0	0.0	0.5	0 ± 0
Blastococcus	0.0	0.0	7.1	0.0	0 ± 0
Marinilactibacillus	0.0	0.0	0.0	1.4	0 ± 0
Candidatus Profftella	0.0	0.0	0.0	0.5	0 ± 0
Parvibaculum	0.0	0.0	0.0	0.5	0 ± 0
Rhodoplanes	0.0	0.0	0.0	0.5	0 ± 0
Rhizobacter	0.0	0.0	0.0	0.5	0 ± 0
OM60(NOR5) clade	0.0	0.0	0.0	0.5	0 ± 0
BAL58 marine group	0.0	0.0	0.0	0.5	0 ± 0
Paucibacter	0.0	0.0	0.0	0.5	0 ± 0
Niastella	0.0	0.0	0.0	0.5	0 ± 0
Candidatus Rhodoluna	0.0	0.0	0.0	0.5	0 ± 0
Pelotomaculum	0.0	0.0	0.0	0.5	0 ± 0
Terracoccus	0.0	0.0	0.0	0.5	0 ± 0
Chitinophaga	0.0	0.0	0.0	0.5	0 ± 0
Segetibacter	0.0	0.0	0.0	0.5	0 ± 0
Candidatus Aquirestis	0.0	0.0	0.0	0.5	0 ± 0
Mucilaginibacter	0.0	0.0	0.0	0.5	0 ± 0
Levilinea	0.0	0.0	0.0	0.5	0 ± 0
Fictibacillus	0.0	0.0	0.0	0.5	0 ± 0
Candidatus Methylomirabilis	0.0	0.0	0.0	0.5	0 ± 0
Oceaniovalibus	0.0	0.0	0.0	0.5	0 ± 0

Rhodovastum	0.0	0.0	0.0	0.5	0 ± 0
Ferriphaselus	0.0	0.0	0.0	0.5	0 ± 0
Candidatus Branchiomonas	0.0	0.0	0.0	0.5	0 ± 0
Desulforegula	0.0	0.0	0.0	0.5	0 ± 0
SEEP-SRB4	0.0	0.0	0.0	0.5	0 ± 0
Geothermobacter	0.0	0.0	0.0	0.5	0 ± 0
Syntrophus	0.0	0.0	0.0	0.5	0 ± 0
Natranaerovirga	0.4	0.0	0.0	0.0	0 ± 0
Gordonibacter	0.4	0.0	0.0	0.0	0 ± 0
Catonella	0.7	0.0	0.0	0.0	0 ± 0
Advenella	0.0	0.0	0.0	0.9	0 ± 0
vadinBC27 wastewater-sludge group	0.0	0.0	0.0	0.5	0 ± 0
Tatumella	0.0	0.0	0.0	0.5	0 ± 0
Burkholderia	0.0	0.0	0.0	0.5	0 ± 0
Shimwellia	0.0	0.0	0.0	0.5	0 ± 0
Rheinheimera	0.0	0.0	0.0	0.5	0 ± 0
Ornithinimicrobium	0.0	0.0	0.0	0.5	0 ± 0
Streptacidiphilus	0.0	0.0	0.0	0.5	0 ± 0
Brevifollis	0.0	0.0	0.0	0.5	0 ± 0
Granulicatella	0.0	0.0	0.0	0.5	0 ± 0
Methylophaga	0.0	0.0	0.0	0.5	0 ± 0
Brachybacterium	0.0	0.0	0.0	0.5	0 ± 0
Tetragenococcus	0.0	0.0	0.0	0.5	0 ± 0
Helcobacillus	0.0	0.0	0.0	0.5	0 ± 0
Kitasatospora	0.0	0.0	0.0	0.5	0 ± 0
Thermoactinomyces	0.0	0.0	0.0	0.5	0 ± 0
Truepera	0.0	0.0	0.0	0.5	0 ± 0
Aquimonas	0.0	0.0	0.0	0.5	0 ± 0
Marinobacter	0.0	0.0	0.0	0.5	0 ± 0
Olivibacter	0.0	0.0	0.0	0.5	0 ± 0
Dermatophilus	0.0	0.0	0.0	0.5	0 ± 0
Oerskovia	0.0	0.0	0.0	0.5	0 ± 0
Jonesia	0.0	0.0	0.0	0.5	0 ± 0
Plantactinospora	0.0	0.0	0.0	0.5	0 ± 0
Atopococcus	0.0	0.0	0.0	0.5	0 ± 0
Pseudofulvimonas	0.0	0.0	0.0	0.5	0 ± 0
Oceanobacillus	0.0	0.0	0.0	0.5	0 ± 0
Prauserella	0.0	0.0	0.0	0.5	0 ± 0
Iodobacter	0.0	0.0	0.0	0.5	0 ± 0
Chungangia	0.0	0.0	0.0	0.5	0 ± 0
Gallibacterium	0.0	0.0	0.0	0.5	0 ± 0
